# Supplementary material for: Diblock dialternating terpolymers by one-step/one-pot highly selective organocatalytic multimonomer polymerization
Source: Nat Commun. 2021 Dec 8;12:7124. doi: 10.1038/s41467-021-27377-3 (PMC8655074; doi:10.1038/s41467-021-27377-3)
Supplement: Supplementary file 1 — Supplementary Information [file 41467_2021_27377_MOESM1_ESM.pdf]

Diblock dialternating terpolymers by one-step/one-pot highly selective organocatalytic multimonomer polymerization

Jiaxi Xu, Xin Wang, Nikos Hadjichristidis*

King Abdullah University of Science and Technology (KAUST), Physical Sciences and Engineering Division, KAUST Catalysis Center, Polymer Synthesis Laboratory, Thuwal 23955, Saudi Arabia

Corresponding author email: nikolaos.hadjichristidis@kaust.edu.sa

Inventory of Supporting Information

Supplementary methods 1

Supplementary Note 1. Terpolymerizations of TAz, PA, and PO 3

Supplementary Note 2. Terpolymerizations of TAz, PA, and EO 8

Supplementary Note 3. Terpolymerizations of TAz, PA, and BO 12

Supplementary Note 4. Terpolymerizations of TAz, PA, and PO initiated by 1,4-benzenedimethanol 14

Supplementary Note 5. Terpolymerizations of TAz, PA, and PO initiated by polyethylene glycol-8000 17

Supplementary Note 6. Terpolymerizations of TAz, PA, and other epoxides 20

Supplementary Note 7. Terpolymerizations of PA, and PO with other *N*-sulfonyl aziridines 24

Supplementary Note 8. Terpolymerizations of TAz, PA, and PO with different bases catalysts 28

Supplementary Note 9. Terpolymerizations of TAz, PA, and PO with different monomer feed ratios 31

Supplementary Note 10. Multiple switches 33

Supplementary Note 11. Kinetic studies 36

Supplementary Note 12. Mechanism 39

Supplementary Note 13. Thermal analysis 48

Supplementary Note 14. Reactivity ratio calculation 51

Supplementary methods

Synthesis of *N*-brosylaziridine (BAz)

Ethanolamine (1.52 g, 25 mmol, 1.0 equiv) in 40 mL pyridine was added in a flamed-dried roundbottomed flask. After the mixture was cooled to -40°C, 4-bromobenzenesulfonyl chloride (13.29 g, 52 mmol, 2.2 equiv) in 20 mL pyridine was added dropwise into the mixture at -40°C with stirring for 30 min. The reaction mixture was stirred at room temperature for 5 h. A mixture of water/ice was added to the mixture. The residue was filtered off and kept aside. The filtrate was extracted with CH_2Cl_2 (2 x 40 mL). The combined organic layer was dried over Na_2SO_4, filtered, and concentrated under vacuum. The crude product [2-((4-bromophenyl)sulfonamido)ethyl 4-bromobenzenesulfonate] was purified on a silica gel column chromatography (R_f = 0.24, EtOAc/petroleum ether = 1/3) as a white solid (8.75 g, 70 %).

The previous product [2-((4-bromophenyl)sulfonamido)ethyl 4-bromobenzenesulfonate] (8.75 g, 17.5 mmol, 1.0 equiv) was dissolved in toluene (100 mL). A 20 wt. % solution of KOH (3.34 g in 25 mL, 60 mmol, 3.4 equiv) was added slowly. After 1 h, the organic phase was separated and washed with water until a neutral pH of the aqueous phase. The organic phase was dried over anhydrous Na_2SO_4, filtered, and concentrated under vacuum. The crude product was purified by column chromatography (R_f = 0.4, EtOAc/petroleum ether = 1/3) to afford the desired compound as a white solid (4.36 g, 95%). ^{1}H NMR ($CDCl_3$): 7.82 (d, *J* = 8.6 Hz, 2H), 7.70 (d, *J* = 8.6 Hz, 2H), 2.41 (s, 4H). ^{13}C NMR ($CDCl_3$): 137.2, 132.6, 129.6, 129.0, 27.2.

Synthesis of *N*-(4-nitrobenzenesulfonyl)aziridine (NAz)

Ethanolamine (2.68 g, 44 mmol, 1.0 equid) and Et_3N (2.0 mL, 13.84 mmol, 0.32 equid) in 40 mL anhydrous CH_2Cl_2 were added in a flamed-dried round-bottomed flask. After the mixture was cooled to 0°C, 4-nitrobenzenesulfonylchloride (9.76 g, 44 mmol, 1.0 equid) in anhydrous CH_2Cl_2 (10 mL) were added into the mixture. The reaction mixture was stirred at room temperature for 18 h. The solvent was removed under reduced pressure before adding EtOAc (130 mL). The organic layer was washed by 1M aqueous NaOH (40 mL), saturated aqueous $NaHCO_3$ (40 mL), brine (40 mL), and dried over anhydrous Na_2SO_4, filtered and concentrated. The crude product *N*-(2-hydroxyethyl)-4-nitrobenzenesulfonamide was purified on a silica gel column chromatography (R_f = 0.22, EtOAc/petroleum ether = 1/1) as pale yellow solid (8.51 g, 79%). ^{1}H NMR (DMSO-d_6): 8.41 (d, *J* = 8.8 Hz, 2H), 8.05 (d, *J* = 8.8 Hz, 2H), 4.73 (t, *J* = 5.5 Hz, 1H), 3.35 (m, 2H), 2.86 (q, *J* = 6.1 Hz, 2H).

The previous product *N*-(2-hydroxyethyl)-4-nitrobenzenesulfonamide (8.51 g, 34.56 mmol, 1.0 equid) and Et_3N (6.02 mL, 41.47 mmol, 1.2 equid) in 85 mL anhydrous CH_2Cl_2 were added in a flamed-dried round-bottomed flask. After 5 min of stirring, ethylsulfonyl chloride (4.43 g, 34.56 mmol, 1.0 equid) in 10 mL anhydrous CH_2Cl_2 was added dropwise into the mixture at 0^oC with stirring for 30 min. The reaction mixture was stirred at room temperature for 18 h. The solvent was removed under reduced pressure before adding EtOAc (420 mL). The organic layer was washed by saturated aqueous $NaHCO_3$ (90 mL), brine (90 mL), and dried over anhydrous Na_2SO_4, filtered, and concentrated. The crude product 2-((4-nitrophenyl)sulfonamido)ethyl ethanesulfonate was purified on a silica gel column chromatography (R_f = 0.23, EtOAc/petroleum ether = 2/3) as pale yellow solid (4.18 g, 36%). ^1H NMR (CDCl3): 8.38 (d, *J* = 8.8 Hz, 2H), 8.07 (d, *J* = 8.8 Hz, 2H), 5.22 (t, *J* = 6.0 Hz, 1H), 4.29 (t, *J* = 5.1 Hz. 2H), 3.43-3.39 (m, 2H), 3.16 (q, *J* = 7.4 Hz, 2H), 1.42 (t, *J* = 7.5 Hz, 3H).

The previous product 2-((4-nitrophenyl)sulfonamido)ethyl ethanesulfonate (4.18 g, 12.35 mmol, 1.0 equid) was dissolved in 85 mL benzene. A solution of KOH (4.09 g in 22 mL H_2O, 7.14 mmol, 0.58 equiv) was added slowly. After 1 h, the solvent was removed under reduced pressure before adding EtOAc (250 mL). The organic phase was washed with water (60 mL), brine (60 mL), and dried over anhydrous Na_2SO_4, filtered, and concentrated under vacuum. The crude product was purified by column chromatography (R_f = 0.27, EtOAc/petroleum ether = 1/3,) to afford the desired compound as a pale yellow solid (2.54 g, 90%). ^1H NMR ($CDCl_3$): 8.41 (d, *J* = 8.8 Hz, 2H), 8.17 (d, *J* = 8.8 Hz, 2H), 2.49 (s, 4H).

Supplementary Note 1. Terpolymerizations of TAz, PA, and PO

Supplementary Table 1. Terpolymerizations of TAz, PA, and PO using *t*-BuP$_1$ as catalyst in THF[a]

Entry	Time	Conv. (TAz)[b]/%	Conv.(PA)[b] /%	$M_{n,theo}$[c]/kg mol^{-1}	$M_{n,NMR}$[b]/kg mol^{-1}	*Đ*[d]
1	15 min	51	17	5.54	5.96	1.07
2	30 min	83	28	8.86	9.39	1.06
3	45 min	91	30	9.69	10.2	1.06
4	1.5 h	99	33	10.5	11.2	1.05
5	3 h	99	33	10.5	11.2	1.05
6	4 h	99	33	10.5	11.2	1.06
7	5 h	99	35	10.9	11.8	1.07
8	6 h	99	58	15.2	16.3	1.07
9	8 h	99	74	18.2	20.2	1.09
10	9 h	99	84	20.0	21.5	1.04, 1.05[e]
11	21 h	99	94	21.9	23.6	1.04, 1.05[e]

[a]The terpolymerizations were performed at a ratio of $[TAz]_0/[PA]_0/[BnN(H)Ts]_0/[t\text{-}BuP_1]_0$ = 30/90/1/0.5 ($[TAz]_0$ = 1.0 M in epoxides and THF) at 100°C. [b]Determined by ^1H NMR in $CDCl_3$ using integrals of the characteristic signals. [c]Calculated as follows: (M.W. of Initiator) + ($[TAz]_0/[I]_0$) × conv.(TAz) × (M.W. of TAz + M.W. of PA) + {($[PA]_0/[I]_0$) × conv.(PA) - ($[TAz]_0/[I]_0$) × conv.(TAz)} × (M.W. of epoxides + M.W. of PA). [d]Determined by SEC at 35°C in THF (1.0 mL min^{-1}) using PSt standards. [e]Bimodal peaks.

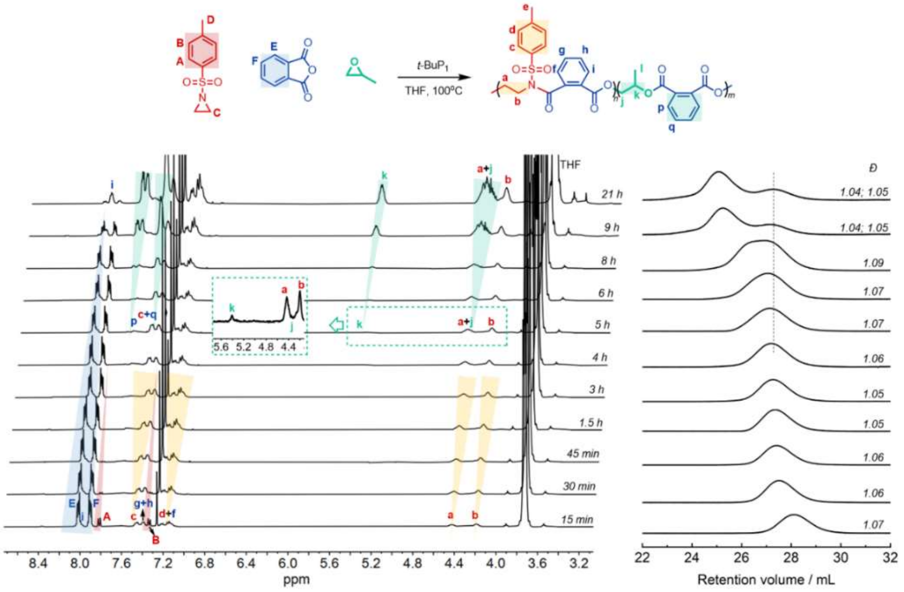

Supplementary Figure 1. Stacked ^1H NMR spectra (400 MHz, $CDCl_3$, 25°C) and SEC traces (THF, 35°C) of the reaction mixture at a ratio of $[TAz]_0/[PA]_0/[BnN(H)Ts]_0/[t\text{-}BuP_1]_0$ = 30/90/1/0.5 in THF (entries 3 and 4 in Table 1, Supplementary Table 1).

Supplementary Table 2. Terpolymerizations of TAz, PA, and PO using t-BuP$_1$ as catalyst[a]

Entry	Time	Conv. (TAz)[b]/%	Conv.(PA)[b] /%	$M_{n,theo}$[c]/kg mol^{-1}	$M_{n,NMR}$[b]/kg mol^{-1}	$Đ$[d]
1	5 min	61	20	6.58	6.91	1.06
2	10 min	93	31	9.90	10.5	1.05
3	15 min	95	32	10.1	10.7	1.05
4	30 min	99	33	10.5	11.2	1.05
5	1 h	99	33	10.5	11.2	1.05
6	2 h	99	48	13.3	14.2	1.04; 1.05[e]
7	3 h	99	76	18.5	19.2	1.03; 1.05[e]
8	4 h	99	99	22.8	24.2	1.03; 1.05[e]

[a]The terpolymerizations were performed at a ratio of [TAz]$_0$/[PA]$_0$/[BnN(H)Ts]$_0$/[t-BuP$_1$]$_0$ = 30/90/1/0.5 ([TAz]$_0$ = 1.0 M in epoxides) at 100°C. [b]Determined by ^{1}H NMR in $CDCl_3$ using integrals of the characteristic signals. [c]Calculated as follows: (M.W. of Initiator) + ([TAz]$_0$/[I]$_0$) × conv.(TAz) × (M.W. of TAz + M.W. of PA) + {([PA]$_0$/[I]$_0$) × conv.(PA) - ([TAz]$_0$/[I]$_0$) × conv.(TAz)} × (M.W. of epoxides + M.W. of PA). [d]Determined by SEC at 35°C in THF (1.0 mL min^{-1}) using PSt standards. [e]Bimodal peaks.

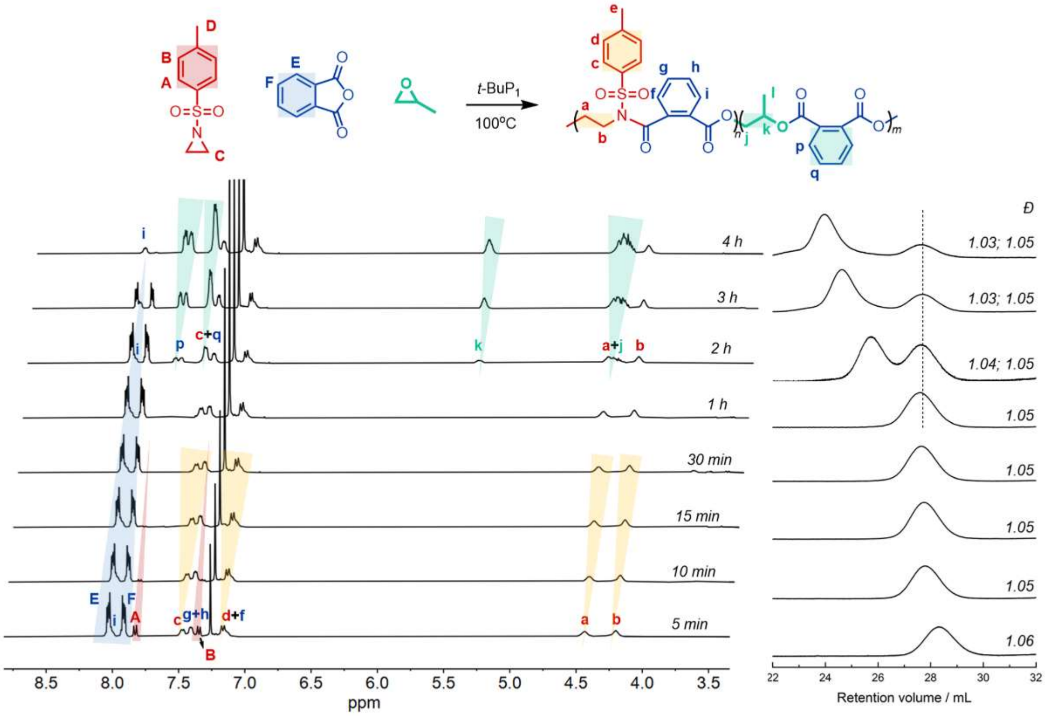

Supplementary Figure 2. Stacked ^{1}H NMR spectra (400 MHz, $CDCl_3$, 25°C) and SEC traces (THF, 35°C) of the reaction mixture at the ratio of [TAz]$_0$/[PA]$_0$/[BnN(H)Ts]$_0$/[t-BuP$_1$]$_0$ = 30/90/1/0.5 in bulk (entries 5 and 6 in Table 1, Supplementary Table 2).

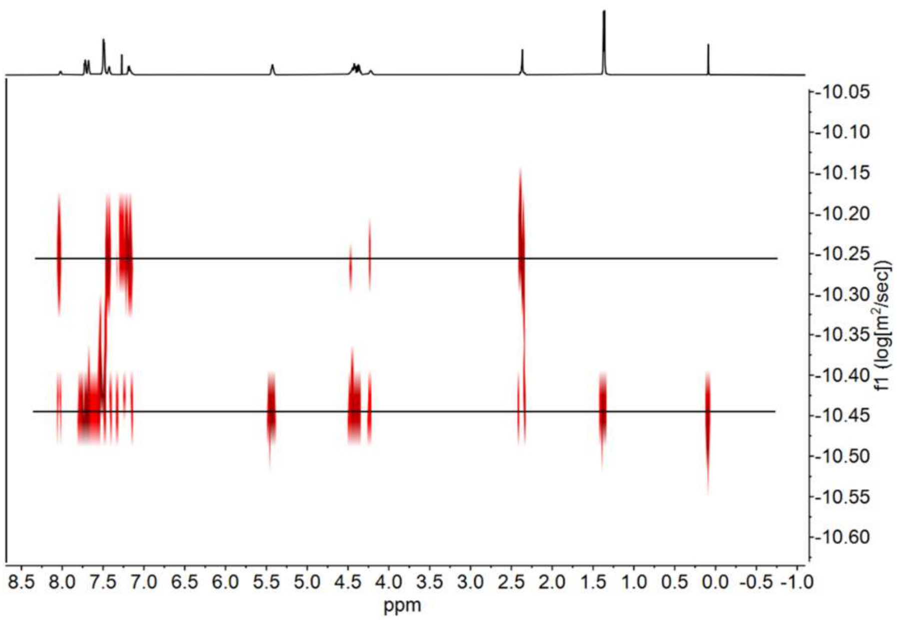

Supplementary Figure 3. DOSY spectrum (600 MHz, $CDCl_3$, 25°C) of the final reaction crude products: poly(TAz-*alt*-PA)-*b*-poly(PA-*alt*-PO) (bottom) and unreacted poly(TAz-*alt*-PA) (top).

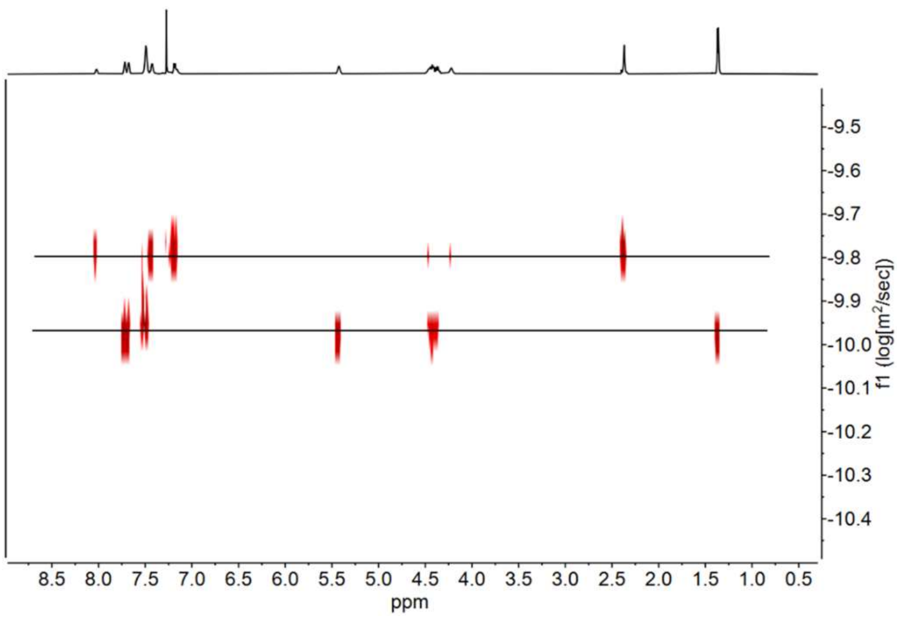

Supplementary Figure 4. DOSY spectrum (600 MHz, $CDCl_3$, 25°C) of blends of copolymers of poly(TAz-*alt*-PA) (top) and poly(PA-*alt*-PO) (bottom).

Supplementary Table 3. Terpolymerizations of TAz, PA, and PO using *t*-BuP$_1$ as catalyst[a]

Entry	Time	Conv. (TAz)[b]/%	Conv.(PA)[b] /%	$M_{n,theo}$[c]/kg mol^{-1}	$M_{n,NMR}$[b]/kg mol^{-1}	$Đ$[d]
1	2 min	48	16	5.23	5.57	1.06
2	5 min	97	32	10.3	10.8	1.03
3	10 min	99	33	10.5	11.2	1.03
4	15 min	99	33	10.5	11.2	1.03
5	30 min	99	33	10.5	11.2	1.03
6	1 h	99	43	12.4	13.4	1.04
7	2 h	99	55	14.6	15.3	1.04
8	3 h	99	73	18.0	19.2	1.03
9	4 h	99	89	20.9	22.2	1.03
10	5 h	99	99	22.8	24.8	1.03

[a]The terpolymerizations were performed at a ratio of $[TAz]_0/[PA]_0/[BnN(H)Ts]_0/[t\text{-}BuP_1]_0$ = 30/90/1/1 ($[TAz]_0$ = 1.0 M in epoxides) at 100°C. [b]Determined by ^{1}H NMR in $CDCl_3$ using integrals of the characteristic signals. [c]Calculated as follows: (M.W. of Initiator) + ($[TAz]_0/[I]_0$) × conv.(TAz) × (M.W. of TAz + M.W. of PA) + {($[PA]_0/[I]_0$) × conv.(PA) - ($[TAz]_0/[I]_0$) × conv.(TAz)} × (M.W. of epoxides + M.W. of PA). [d]Determined by SEC at 35°C in THF (1.0 mL min^{-1}) using PSt standards.

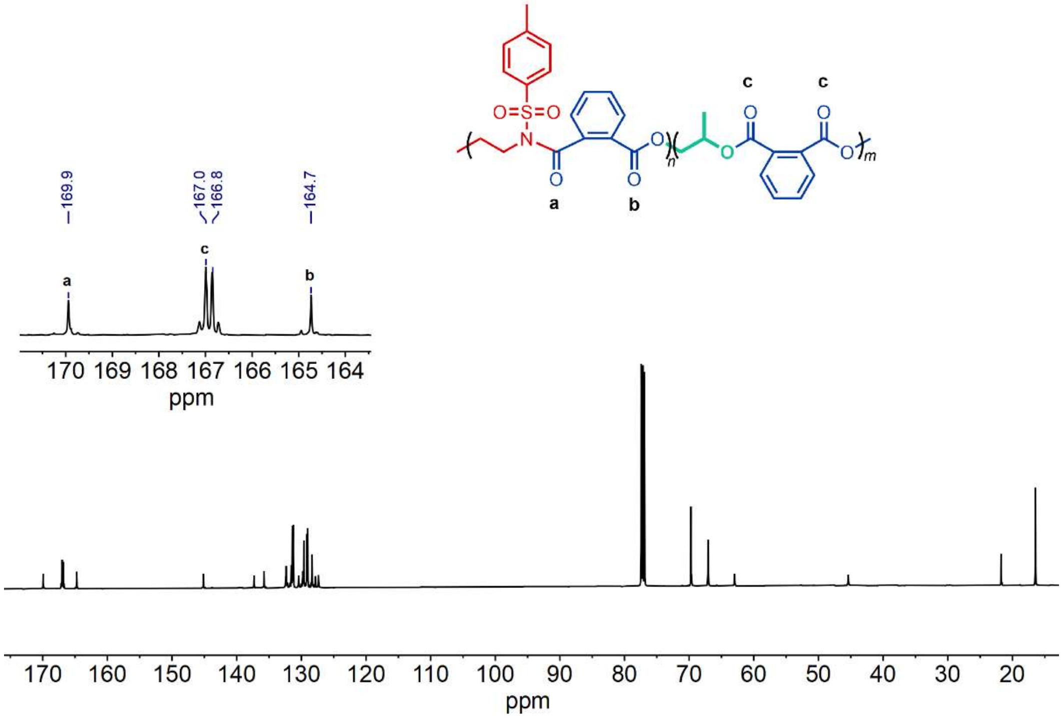

Supplementary Figure 5. ^{13}C NMR spectrum (100 MHz, $CDCl_3$, 25°C) of poly(TAz-*alt*-PA)-*b*-poly(PA-*alt*-PO)

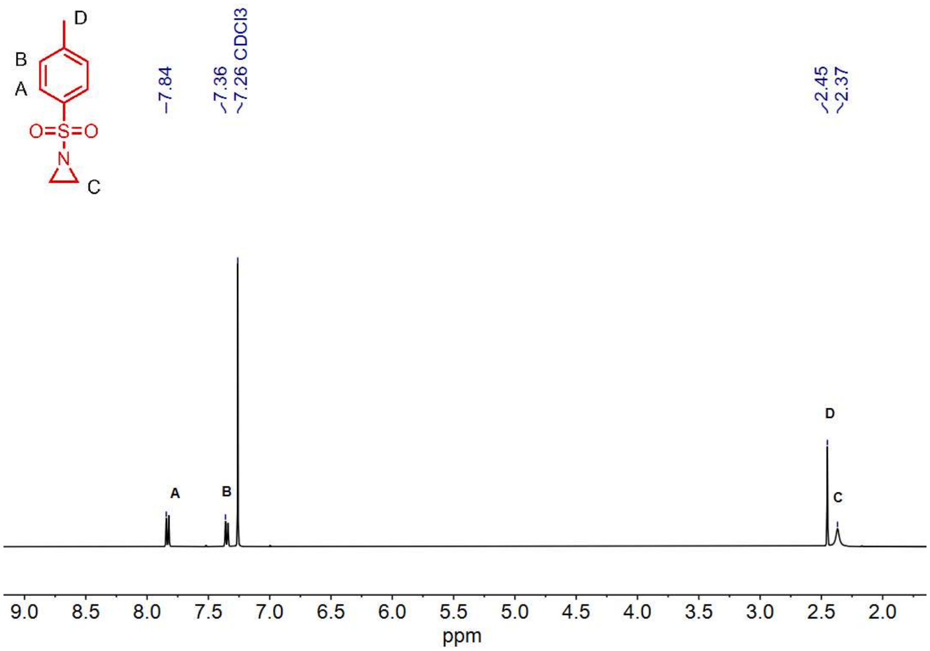

Supplementary Figure 6. ^1H NMR spectrum (400 MHz, $CDCl_3$, 25°C) of TAz.

Supplementary Table 4. Terpolymerizations of TAz, PA, and PO using *t*-BuP$_1$ as catalyst[a]

Entry	Time	Conv. (TAz)[b]/%	Conv.(PA)[b] /%	$M_{n,theo}$[c]/kg mol^{-1}	$M_{n,NMR}$[b]/kg mol^{-1}	*Đ*[d]
1	30 min	98	33	10.4	10.8	1.05
2	1 h	99	33	10.5	11.2	1.05
3	2 h	99	43	12.4	13.5	1.06
4	3 h	99	49	13.5	14.2	1.07
5	4 h	99	57	15.0	16.2	1.04
6	5 h	99	72	17.8	19.0	1.06
7	6 h	99	87	20.6	21.7	1.06

[a]The terpolymerizations were performed at ta ratio $[TAz]_0/[PA]_0/[BnN(H)Ts]_0/[t\text{-}BuP_1]_0$ = 30/90/1/1 ($[TAz]_0$ = 2.0 M in epoxides) at 100°C. [b]Determined by ^1H NMR in $CDCl_3$ using integrals of the characteristic signals. [c]Calculated as follows: (M.W. of Initiator) + ($[TAz]_0/[I]_0$) × conv.(TAz) × (M.W. of TAz + M.W. of PA) + {($[PA]_0/[I]_0$) × conv.(PA) - ($[TAz]_0/[I]_0$) × conv.(TAz)} × (M.W. of epoxides + M.W. of PA). [d]Determined by SEC at 35°C in THF (1.0 mL min^{-1}) using PSt standards.

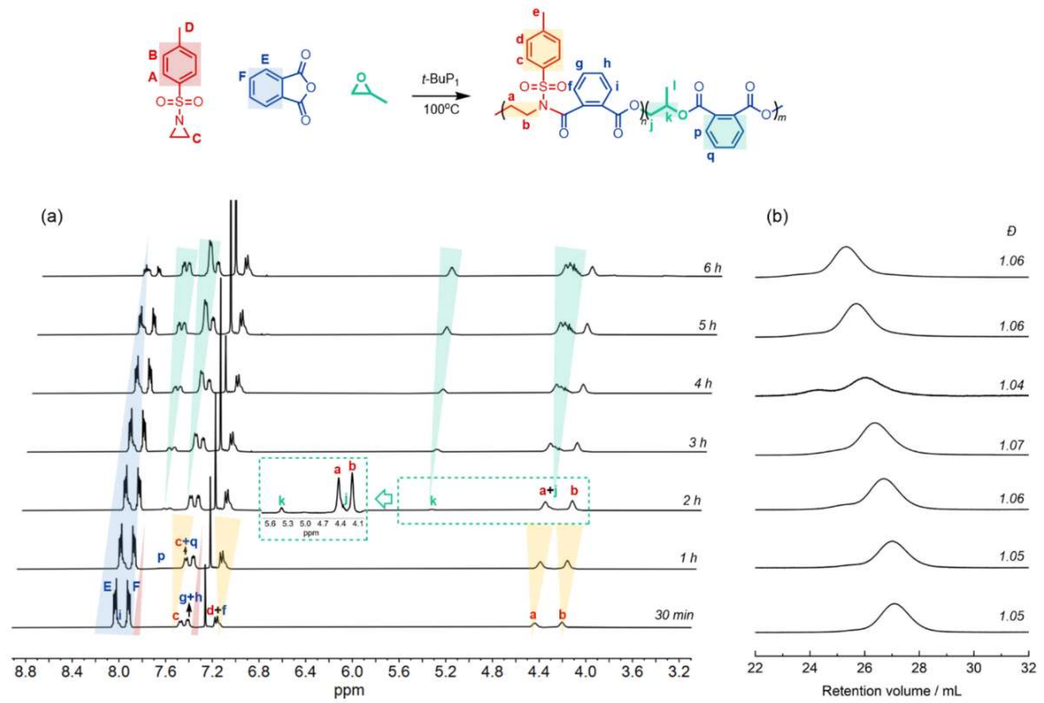

Supplementary Figure 7. Stacked ^{1}H NMR spectra (400 MHz, $CDCl_3$, 25°C) and SEC traces (THF, 35°C) of the reaction mixture at a ratio of $[TAz]_0/[PA]_0/[BnN(H)Ts]_0/[t\text{-}BuP_1]_0$ = 30/90/1/1 ($[TAz]_0$ = 2.0 M in PO) (entries 9 and 10, Table 1).

Supplementary Note 2. Terpolymerizations of TAz, PA, and EO

Supplementary Table 5. Terpolymerizations of TAz, PA, and EO using *t*-BuP$_1$ as catalyst [a]

Entry	Time	Conv. (TAz)[b]/%	Conv.(PA)[b] /%	$M_{n,theo}$[c]/kg mol^{-1}	$M_{n,NMR}$[b]/kg mol^{-1}	*Đ*[d]
1	5 min	87	29	9.28	9.71	1.03
2	10 min	98	33	10.4	10.7	1.03
3	15 min	99	33	10.5	11.2	1.03
4	30 min	99	38	11.4	12.1	1.04
5	45 min	99	40	11.8	12.9	1.05
6	1 h	99	46	12.8	13.5	1.06
7	1.5 h	99	64	15.9	16.5	1.05
8	2 h	99	80	18.7	19.7	1.05
9	2.5 h	99	93	20.9	22.2	1.06
10	3 h	99	99	22.0	24.0	1.08

[a]The terpolymerizations were performed at the ratio of $[TAz]_0/[PA]_0/[BnN(H)Ts]_0/[t\text{-}BuP_1]_0$ = 30/90/1/1 ($[TAz]_0$ = 1.0 M in epoxides) at 100°C. [b]Determined by ^{1}H NMR in $CDCl_3$ using integrals of the characteristic signals. [c]Calculated as follows: (M.W. of Initiator) + ($[TAz]_0/[I]_0$) × conv.(TAz) × (M.W. of TAz + M.W. of PA) + {($[PA]_0/[I]_0$) × conv.(PA) - ($[TAz]_0/[I]_0$) × conv.(TAz)} × (M.W. of epoxides + M.W. of PA). [d]Determined by SEC traces at 35°C in THF (1.0 mL min^{-1}) using PSt standards.

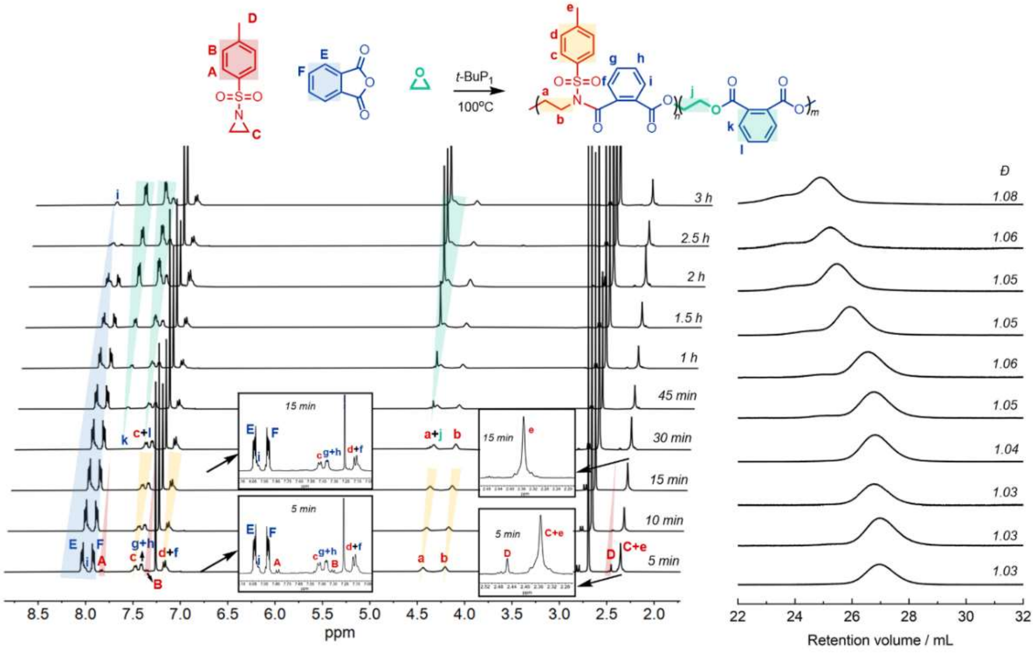

Supplementary Figure 8. Stacked ^{1}H NMR spectra (400 MHz, $CDCl_3$, 25°C) and SEC traces (THF, 35°C) of the reaction mixture at the ratio of $[TAz]_0/[PA]_0/[BnN(H)Ts]_0/[t\text{-}BuP_1]_0$ = 30/90/1/1 ($[TAz]_0$ = 1.0 M in EO) at 100°C (entries 11-13, Table 1).

Supplementary Table 6. Terpolymerizations of TAz, PA, and EO using t-BuP_1 as catalyst[a]

Entry	Time	Conv. (TAz)[b]/%	Conv.(PA)[b] /%	$M_{n,theo}$[c]/kg mol^{-1}	$M_{n,NMR}$[b]/kg mol^{-1}	$Đ$[d]
1	15 min	30	10	3.37	3.54	1.09
2	30 min	57	19	6.17	6.49	1.05
3	45 min	71	24	7.62	8.02	1.05
4	1 h	79	26	8.45	8.95	1.04
5	1.5 h	99	33	10.5	11.2	1.04
6	2 h	99	33	10.5	11.2	1.04
7	4 h	99	33	10.5	11.2	1.04
8	6 h	99	46	12.8	13.9	1.04
9	10 h	99	53	14.0	15.2	1.04
10	20 h	99	70	17.0	18.1	1.04
11	30 h	99	99	22.0	24.2	1.03

[a]The terpolymerizations were performed at a ratio of $[TAz]_0/[PA]_0/[BnN(H)Ts]_0/[t\text{-}BuP_1]_0$ = 30/90/1/1 ($[TAz]_0$ = 1.0 M in epoxides) at 60°C. [b]Determined by ^{1}H NMR in $CDCl_3$ using integrals of the characteristic signals. [c]Calculated as follows: (M.W. of Initiator) + ($[TAz]_0/[I]_0$) × conv.(TAz) × (M.W. of TAz + M.W. of PA) + {($[PA]_0/[I]_0$) × conv.(PA) - ($[TAz]_0/[I]_0$) × conv.(TAz)} × (M.W. of epoxides + M.W. of PA). [d]Determined by SEC at 35°C in THF (1.0 mL min^{-1}) using PSt standards.

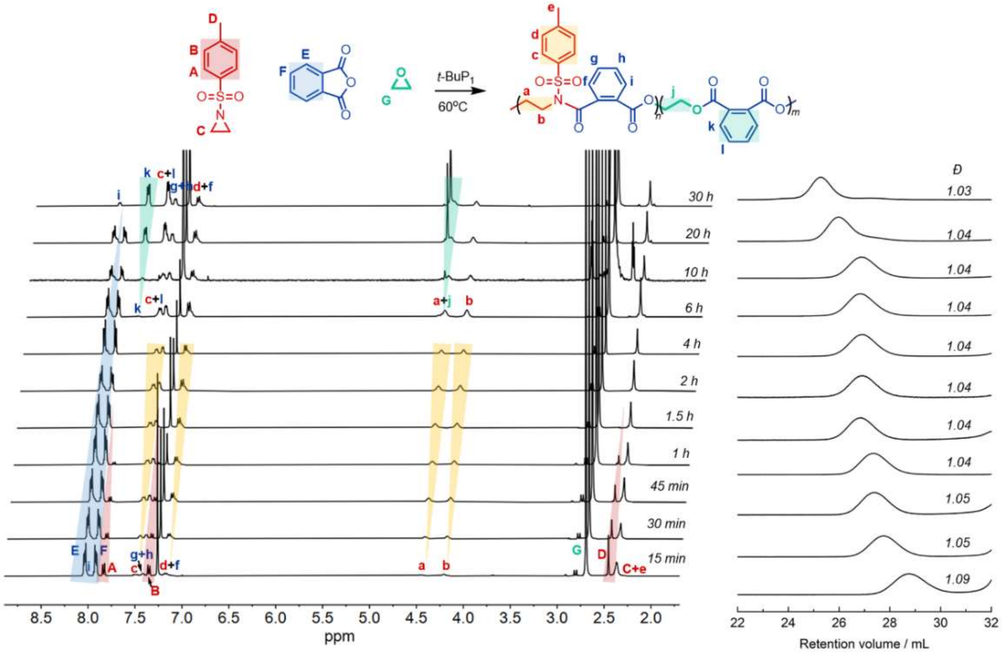

Supplementary Figure 9. Stacked ^{1}H NMR spectra (400 MHz, $CDCl_3$, 25°C) and SEC traces (THF, 35°C) of the reaction mixture at the ratio of $[TAz]_0/[PA]_0/[BnN(H)Ts]_0/[t\text{-}BuP_1]_0$ = 30/90/1/1 ($[TAz]_0$ = 1.0 M in EO) at 60°C (entries 14 and 15, Table 1).

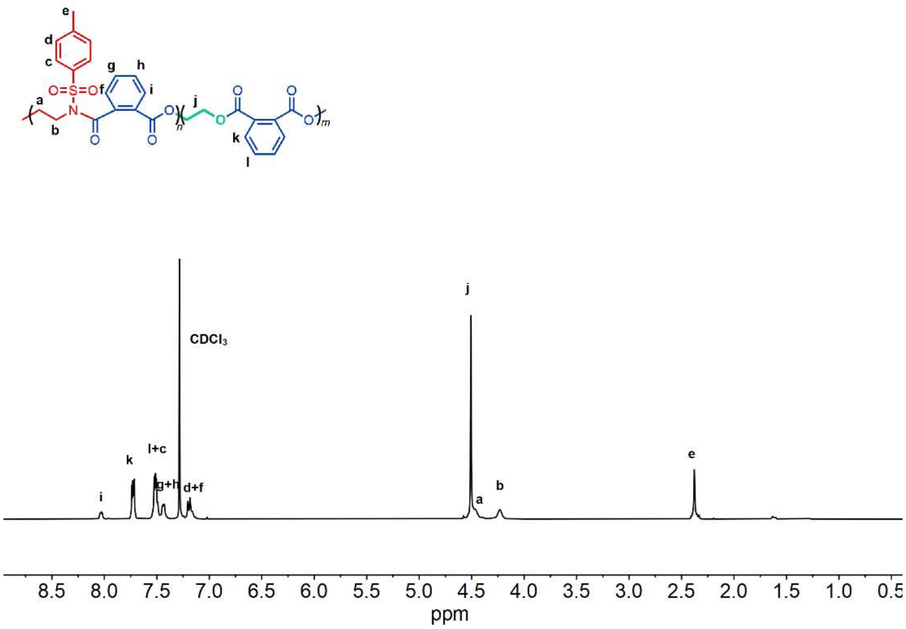

Supplementary Figure 10. ^{1}H NMR spectrum (400 MHz, $CDCl_3$, 25°C) of poly(TAz-*alt*-PA)-*b*-poly(PA-*alt*-EO).

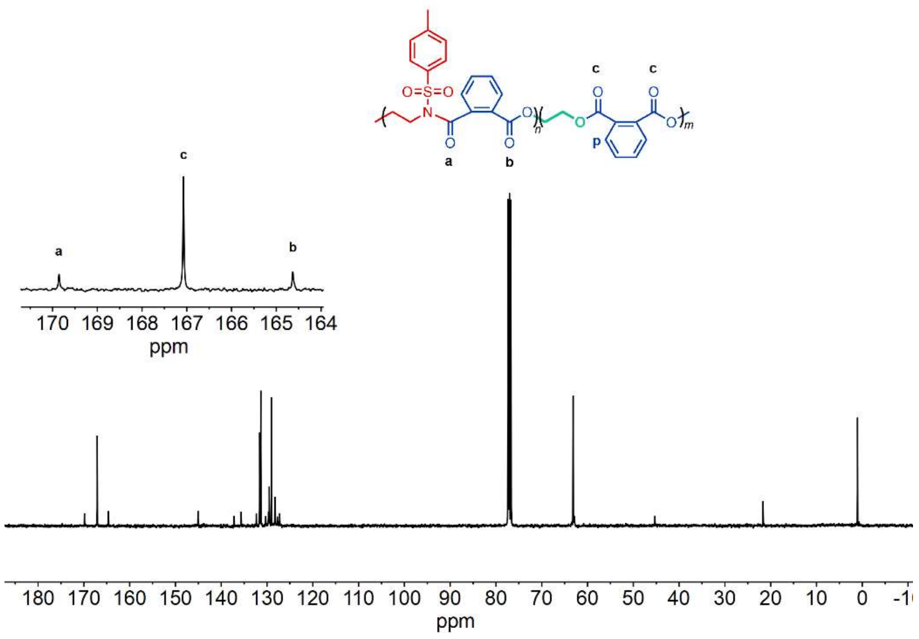

Supplementary Figure 11. ^{13}C NMR spectrum (100 MHz, $CDCl_3$, 25°C) of poly(TAz-*alt*-PA)-*b*-poly(PA-*alt*-EO).

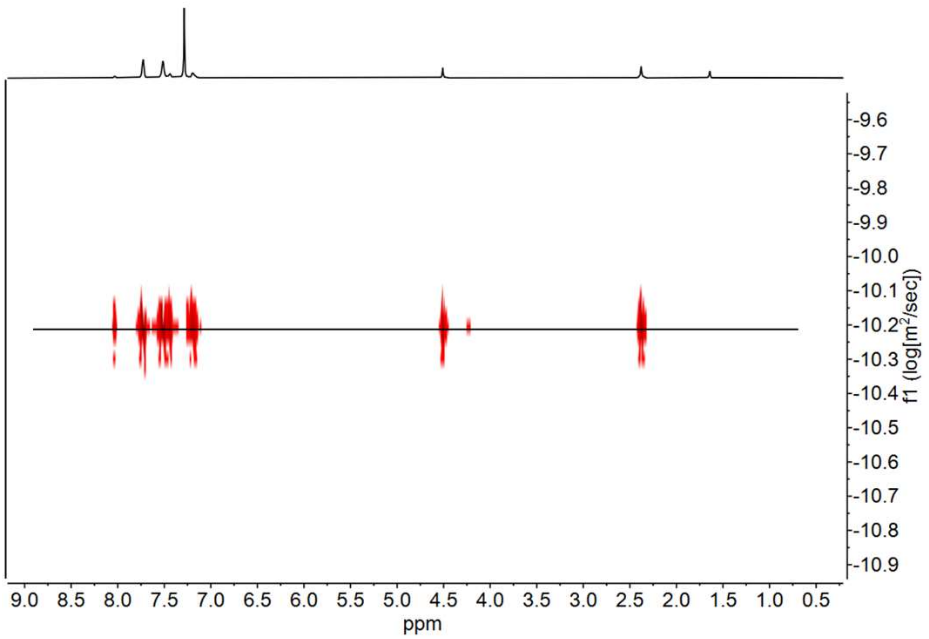

Supplementary Figure 12. DOSY spectrum (600 MHz, $CDCl_3$, 25°C) of poly(TAz-*alt*-PA)-*b*-poly(PA-*alt*-EO).

Supplementary Note 3. Terpolymerizations of TAz, PA, and BO

Supplementary Table 7. Terpolymerizations of TAz, PA, and BO using *t*-BuP$_1$ as catalyst[a]

Entry	Time	Conv. (TAz)[b]/%	Conv.(PA)[b] /%	$M_{n,theo}$[c]/kg mol^{-1}	$M_{n,NMR}$[b]/kg mol^{-1}	*Đ*[d]
1	5 min	84	28	8.96	9.47	1.03
2	10 min	94	31	10.0	10.7	1.03
3	15 min	97	32	10.3	11.1	1.03
4	30 min	99	33	10.5	11.2	1.03
5	1 h	99	46	13.1	13.9	1.04
6	2 h	99	56	15.1	16.2	1.05
7	3 h	99	66	17.1	18.2	1.05
8	4 h	99	87	21.3	23.1	1.04
9	5 h	99	99	23.6	25.1	1.04

[a]The terpolymerizations were performed at a ratio of [TAz]$_0$/[PA]$_0$/[BnN(H)Ts]$_0$/[*t*-BuP$_1$]$_0$ = 30/90/1/1 ([TAz]$_0$ = 1.0 M in epoxides) at 100°C. [b]Determined by ^{1}H NMR in CDCl$_3$ using integrals of the characteristic signals. [c]Calculated as follows: (M.W. of Initiator) + ([TAz]$_0$/[I]$_0$) × conv.(TAz) × (M.W. of TAz + M.W. of PA) + {([PA]$_0$/[I]$_0$) × conv.(PA) - ([TAz]$_0$/[I]$_0$) × conv.(TAz)} × (M.W. of epoxides + M.W. of PA). [d]Determined by SEC at 35°C in THF (1.0 mL min^{-1}) using PSt standards.

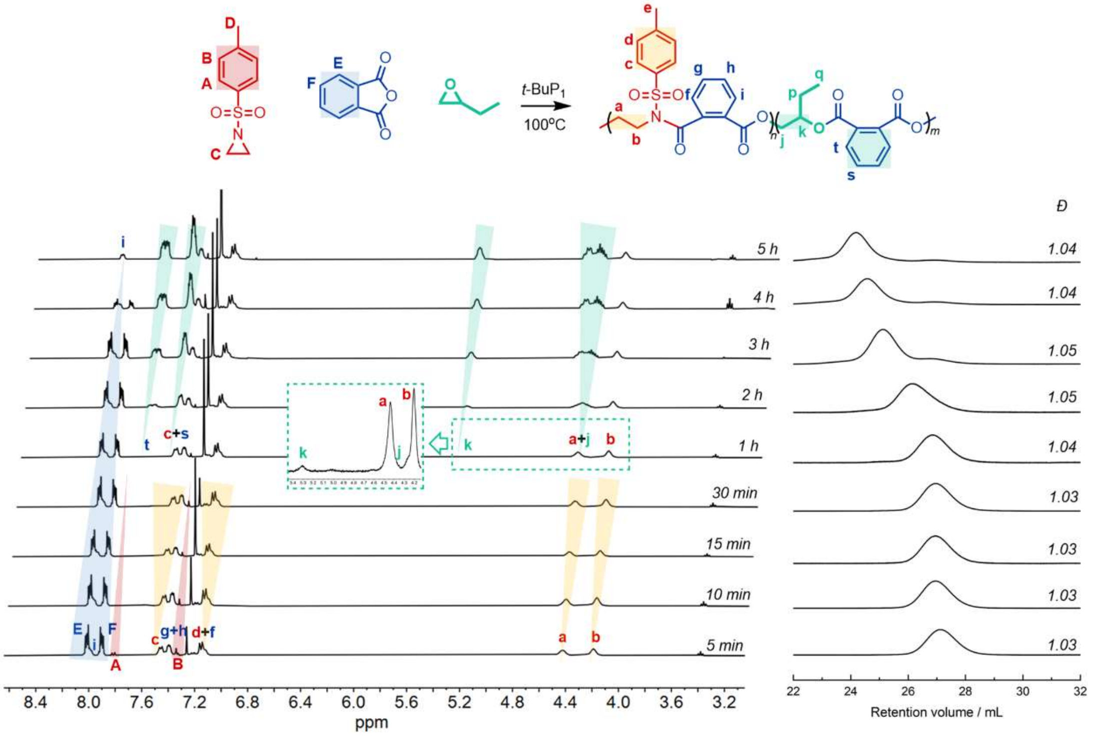

Supplementary Figure 13. Stacked ^{1}H NMR spectra (400 MHz, CDCl$_3$, 25°C) and SEC traces (THF, 35°C) of the reaction mixture at the ratio of [TAz]$_0$/[PA]$_0$/[BnN(H)Ts]$_0$/[*t*-BuP$_1$]$_0$ = 30/90/1/1 ([TAz]$_0$ = 1.0 M in BO) at 100°C (entries 16 and 17, Table 1).

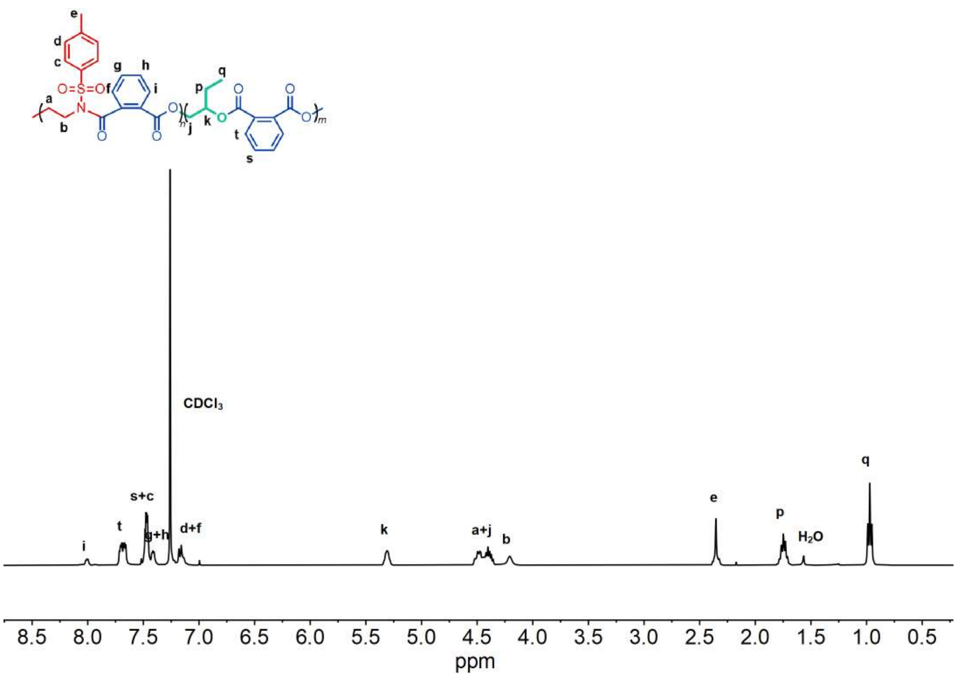

Supplementary Figure 14. ^{1}H NMR spectrum (400 MHz, $CDCl_3$, 25°C) of poly(TAz-*alt*-PA)-*b*-poly(PA-*alt*-BO).

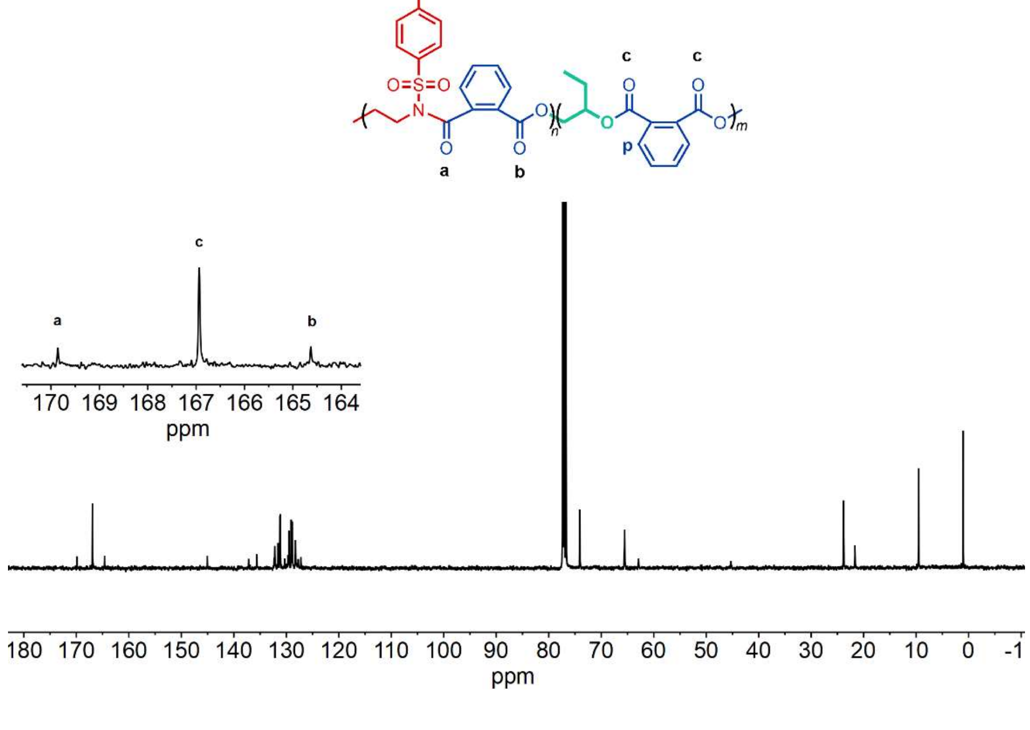

Supplementary Figure 15. ^{13}C NMR spectrum (100 MHz, $CDCl_3$, 25°C) of poly(TAz-*alt*-PA)-*b*-poly(PA-*alt*-BO).

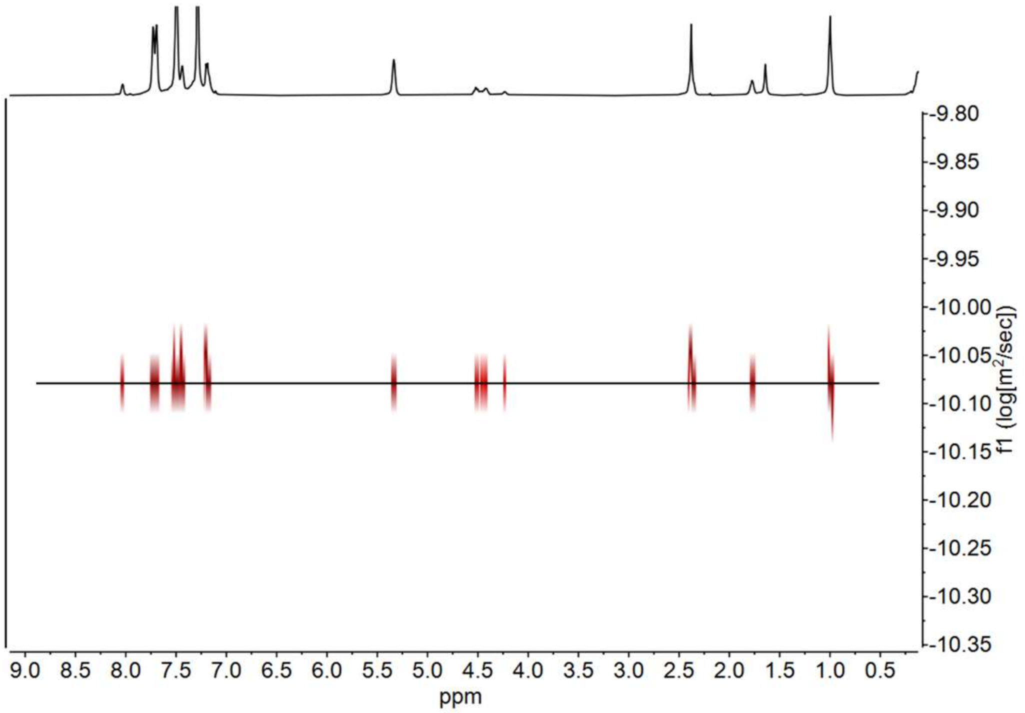

Supplementary Figure 16. DOSY spectrum (600 MHz, $CDCl_3$, 25°C) of poly(TAz-*alt*-PA)-*b*-poly(PA-*alt*-BO).

Supplementary Note 4. Terpolymerizations of TAz, PA, and PO initiated by 1,4-benzenedimethanol

Supplementary Table 8. Terpolymerizations of TAz, PA, and PO using t-BuP_1 as catalyst[a]

Entry	Time	Conv. (TAz)[b]/%	Conv.(PA)[b] /%	$M_{n,theo}$[c]/kg mol^{-1}	$M_{n,NMR}$[b]/kg mol^{-1}	$Đ$[d]
1	5 min	94	31	9.88	10.7	1.03
2	10 min	97	32	10.2	10.8	1.04
3	15 min	99	33	10.4	11.2	1.04
4	1 h	99	33	10.4	11.2	1.07
5	2 h	99	50	13.6	14.6	1.10
6	3 h	99	56	14.7	15.8	1.10
7	4 h	99	74	18.0	19.4	1.09
8	5 h	99	94	21.8	23.8	1.10

[a]The terpolymerizations were performed at aratio $[TAz]_0/[PA]_0/[1,4\text{-benzenedimethanol}]_0/[t\text{-BuP}_1]_0$ = 30/90/1/1 ($[TAz]_0$ = 1.0 M in epoxides) at 100°C. [b]Determined by ^{1}H NMR in $CDCl_3$ using integrals of the characteristic signals. [c]Calculated as follows: (M.W. of Initiator) + ($[TAz]_0/[I]_0$) × conv.(TAz) × (M.W. of TAz + M.W. of PA) + {($[PA]_0/[I]_0$) × conv.(PA) - ($[TAz]_0/[I]_0$) × conv.(TAz)} × (M.W. of epoxides + M.W. of PA). [d]Determined by SEC at 35°C in THF (1.0 mL min^{-1}) using PSt standards.

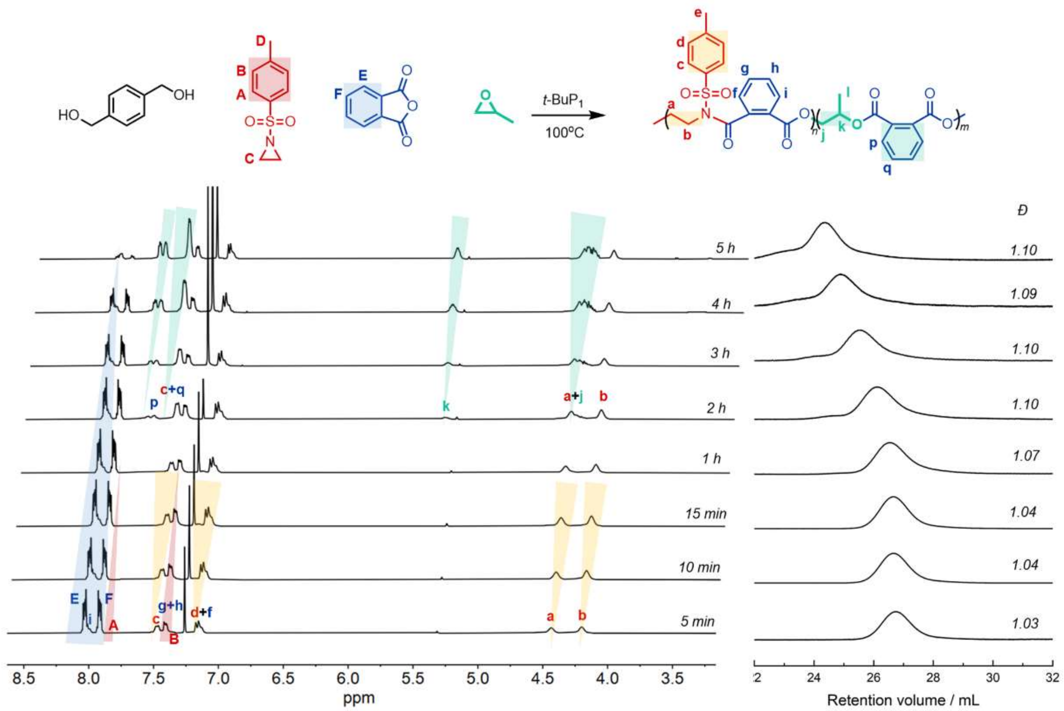

Supplementary Figure 17. Stacked ^{1}H NMR spectra (400 MHz, $CDCl_3$, 25°C) and SEC traces (THF, 35°C) of the reaction mixture at the ratio of $[TAz]_0/[PA]_0/[1,4\text{-benzenedimethanol}]_0/[t\text{-BuP}_1]_0$ = 30/90/1/1 ($[TAz]_0$ = 1.0 M in PO) at 100°C (entries 18 and 19, Table 1).

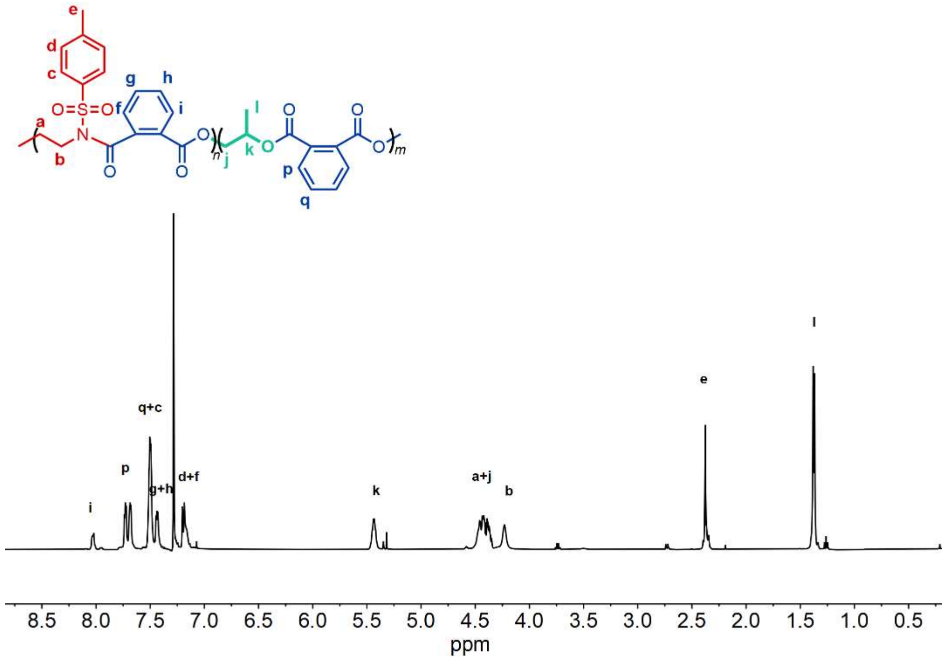

Supplementary Figure 18. ^{1}H NMR spectrum (400 MHz, $CDCl_3$, 25°C) of poly(TAz-*alt*-PA)-*b*-poly(PA-*alt*-PO) initiated by 1,4-benzenedimethanol.

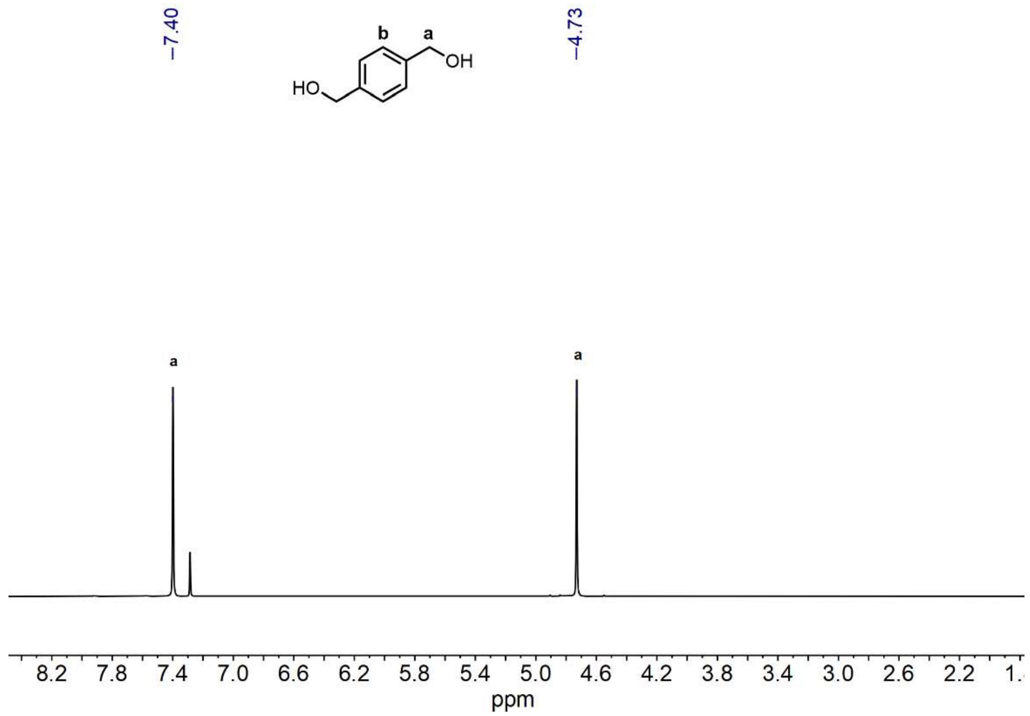

Supplementary Figure 19. ^{1}H NMR spectrum (400 MHz, $CDCl_3$, 25°C) of 1,4-benzenedimethanol.

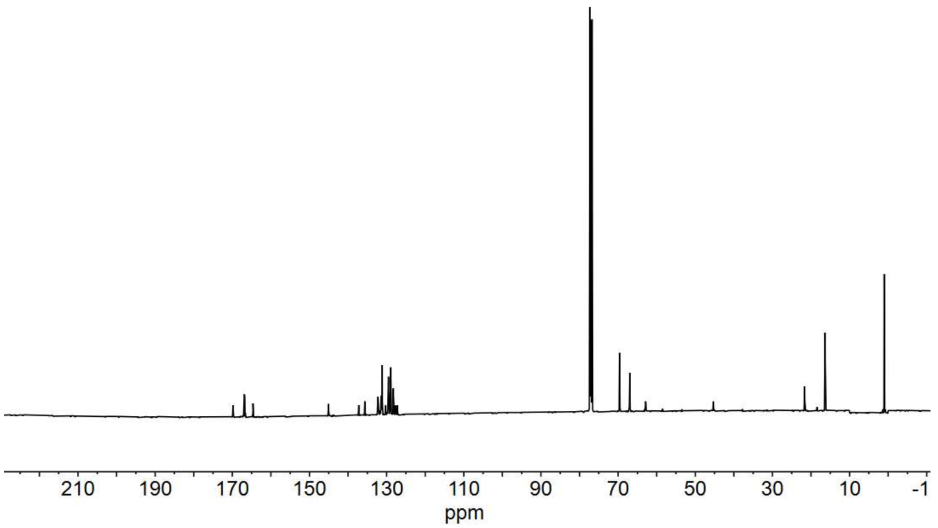

Supplementary Figure 20. ^{13}C NMR spectrum (100 MHz, $CDCl_3$, 25°C) of poly(TAz-*alt*-PA)-*b*-poly(PA-*alt*-PO) initiated by 1,4-benzenedimethanol.

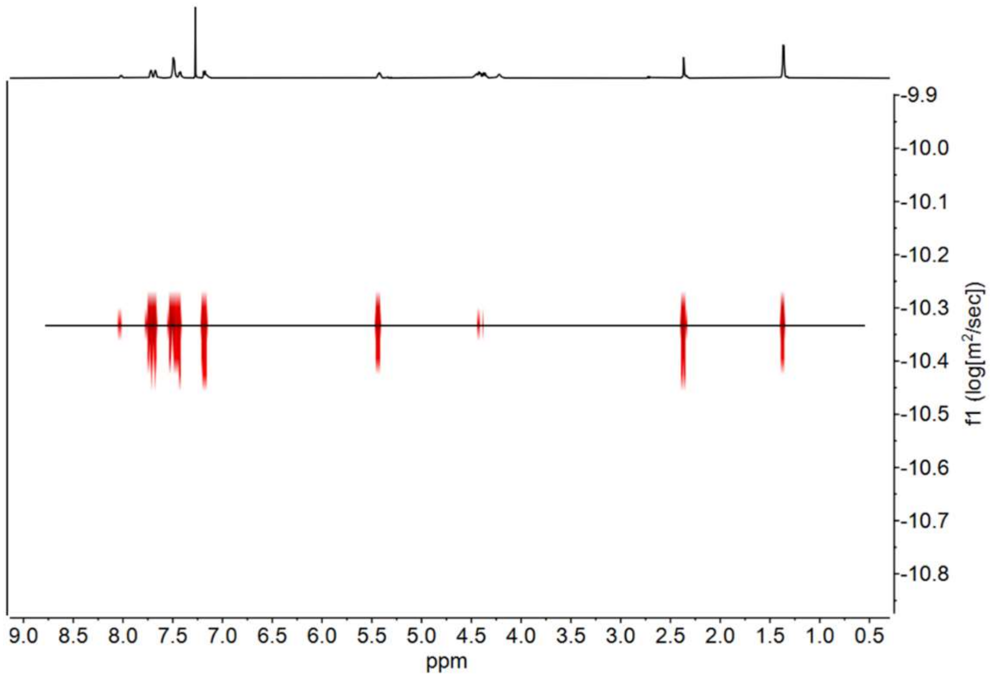

Supplementary Figure 21. DOSY spectrum (600 MHz, $CDCl_3$, 25°C) of poly(TAz-*alt*-PA)-*b*-poly(PA-*alt*-BO)) initiated by 1,4-benzenedimethanol.

Supplementary Note 5. Terpolymerizations of TAz, PA, and PO initiated by polyethylene glycol-8000

Supplementary Table 9. Terpolymerizations of TAz, PA, and PO using *t*-BuP_1 as catalyst[a]

Entry	Time	Conv. (TAz)[b]/%	Conv.(PA)[b] /%	$M_{n,theo}$[c]/kg mol^{-1}	$M_{n,NMR}$[b]/kg mol^{-1}	$Đ$[d]
1	5 min	93	31	17.6	18.5	1.01
2	10 min	97	32	18.1	19.4	1.02
3	15 min	99	33	18.3	19.6	1.02
4	30 min	99	33	18.3	19.6	1.02
5	1 h	99	42	20.0	21.9	1.02
6	2 h	99	73	25.7	27.1	1.02
7	3 h	99	99	30.5	32.0	1.03

[a]The terpolymerizations were performed at a ratio of $[TAz]_0/[PA]_0/[\text{polyethylene glycol}]_0/[t\text{-}BuP_1]_0$ = 30/90/1/1 ($[TAz]_0$ = 1.0 M in epoxides) at 100°C. [b]Determined by ^1H NMR in $CDCl_3$ using integrals of the characteristic signals. [c]Calculated as follows: (M.W. of Initiator) + ($[TAz]_0/[I]_0$) × conv.(TAz) × (M.W. of TAz + M.W. of PA) + {($[PA]_0/[I]_0$) × conv.(PA) - ($[TAz]_0/[I]_0$) × conv.(TAz)} × (M.W. of epoxides + M.W. of PA). [d]Determined by SEC at 35°C in THF (1.0 mL min^{-1}) using PSt standards.

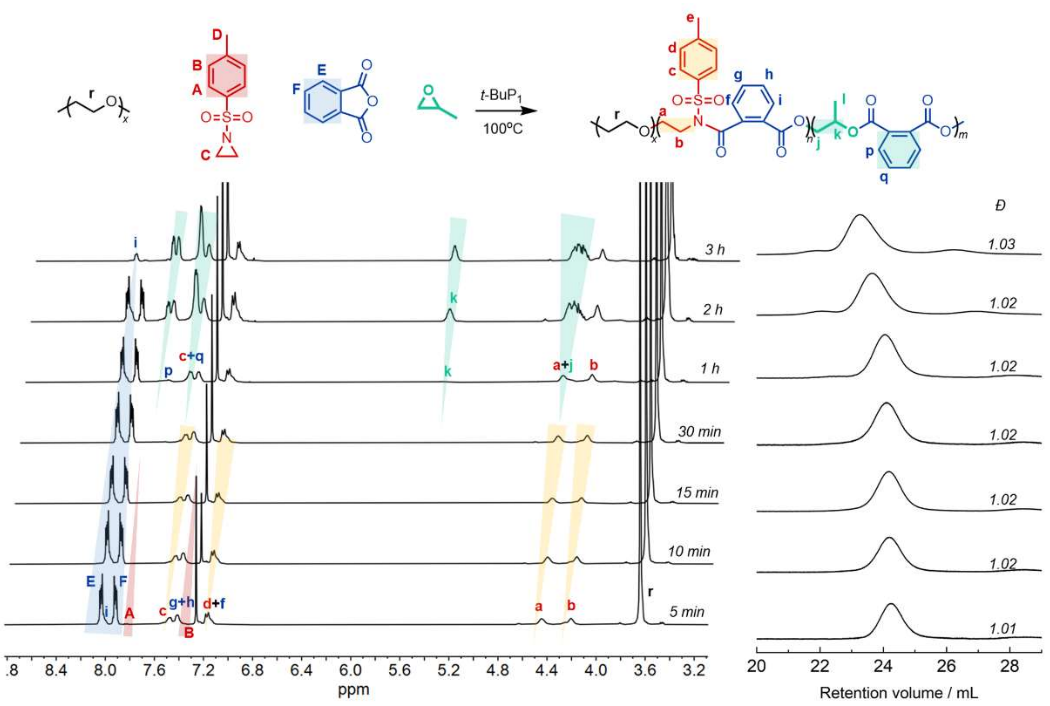

Supplementary Figure 22. Stacked ^{1}H NMR spectra (400 MHz, $CDCl_3$, 25°C) and SEC traces (THF, 35°C) of the reaction mixture at the ratio of [TAz]$_0$/[PA]$_0$/[polyethylene glycol-8000]$_0$/[t-BuP$_1$]$_0$ = 30/90/1/1 ([TAz]$_0$ = 1.0 M in PO) at 100°C (entries 20 and 21, Table 1).

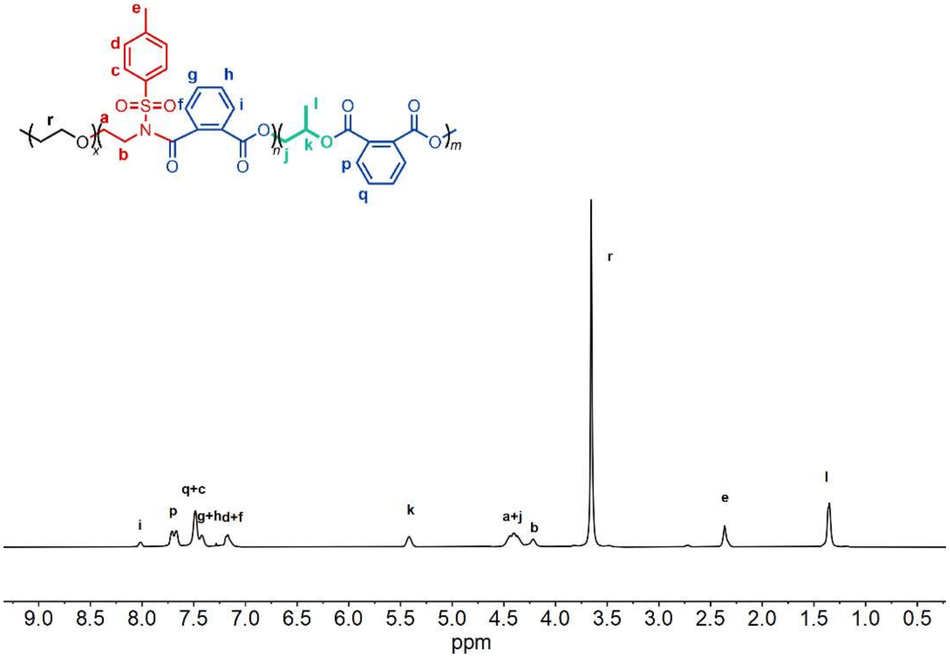

Supplementary Figure 23. ^{1}H NMR spectrum (400 MHz, $CDCl_3$, 25°C) of poly(TAz-*alt*-PA)-*b*-poly(PA-*alt*-PO) initiated by polyethylene glycol-8000.

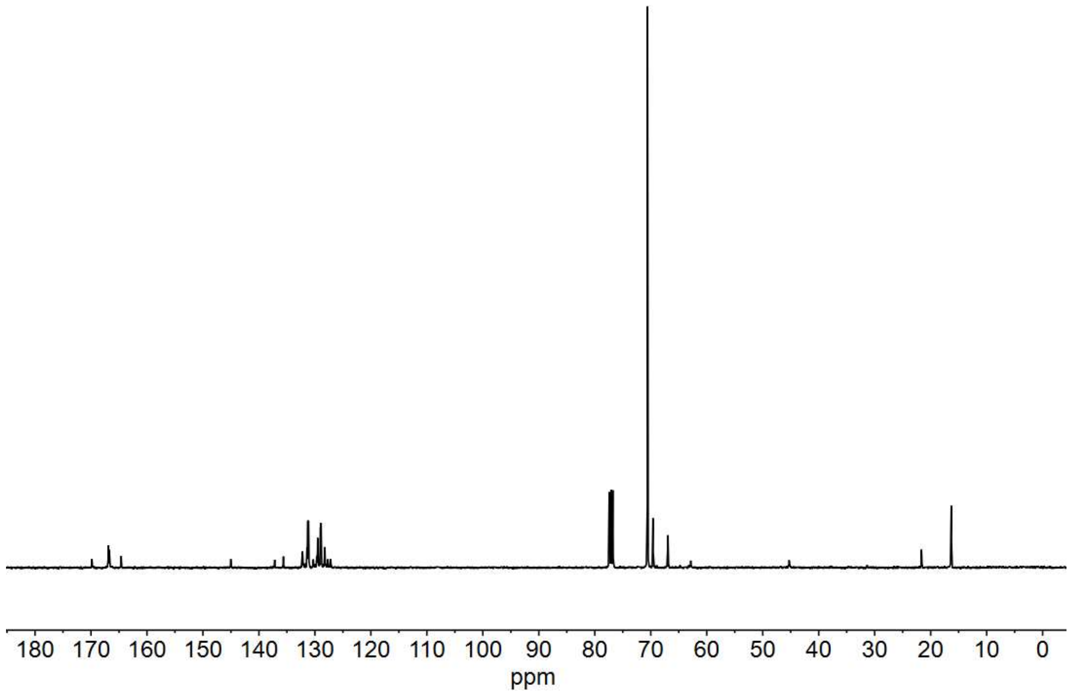

Supplementary Figure 24. ^{13}C NMR spectrum (100 MHz, $CDCl_3$, 25°C) of poly(TAz-*alt*-PA)-*b*-poly(PA-*alt*-PO) initiated by polyethylene glycol-8000.

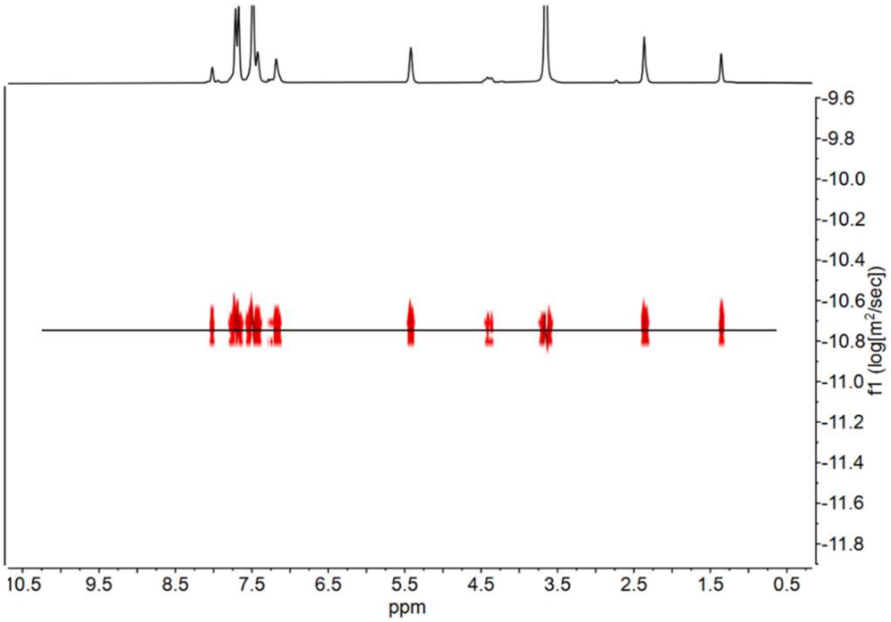

Supplementary Figure 25. DOSY spectrum (600 MHz, $CDCl_3$, 25°C) of poly(TAz-*alt*-PA)-*b*-poly(PA-*alt*-PO) initiated by polyethylene glycol-8000.

Supplementary Note 6. Terpolymerizations of TAz, PA, and other epoxides

Supplementary Table 10. Terpolymerizations of TAz, PA, and SO using t-BuP$_1$ as catalyst[a]

Entry	Time	Conv. (TAz)[b]/%	Conv.(PA)[b] /%	$M_{n,theo}$[c]/kg mol^{-1}	$M_{n,SEC}$[d]/kg mol^{-1}	$Đ$[d]
1	5 min				4.02	1.14
2	10 min				6.19	1.10
3	15 min				7.27	1.08
4	30 min	99		10.5	7.53	1.08
5	1 h	99			7.60	1.09
6	2 h	99			8.57	1.15
7	3 h	99	99	26.6	9.97	1.03; 1.08

[a]The terpolymerizations were performed at a ratio of $[TAz]_0/[PA]_0/[Bn(Ts)NH]_0/[t\text{-BuP}_1]_0$ = 30/90/1/1 ($[TAz]_0$ = 1.0 M in SO) at 100°C. [b]Determined by ^1H NMR in $CDCl_3$. [c]Calculated as follows: (M.W. of Initiator) + ($[TAz]_0/[I]_0$) × conv.(TAz) × (M.W. of TAz + M.W. of PA) + {($[PA]_0/[I]_0$) × conv.(PA) - ($[TAz]_0/[I]_0$) × conv.(TAz)} × (M.W. of epoxides + M.W. of PA). [d]Determined by SEC at 35°C in THF (1.0 mL min^{-1}) using PSt standards.

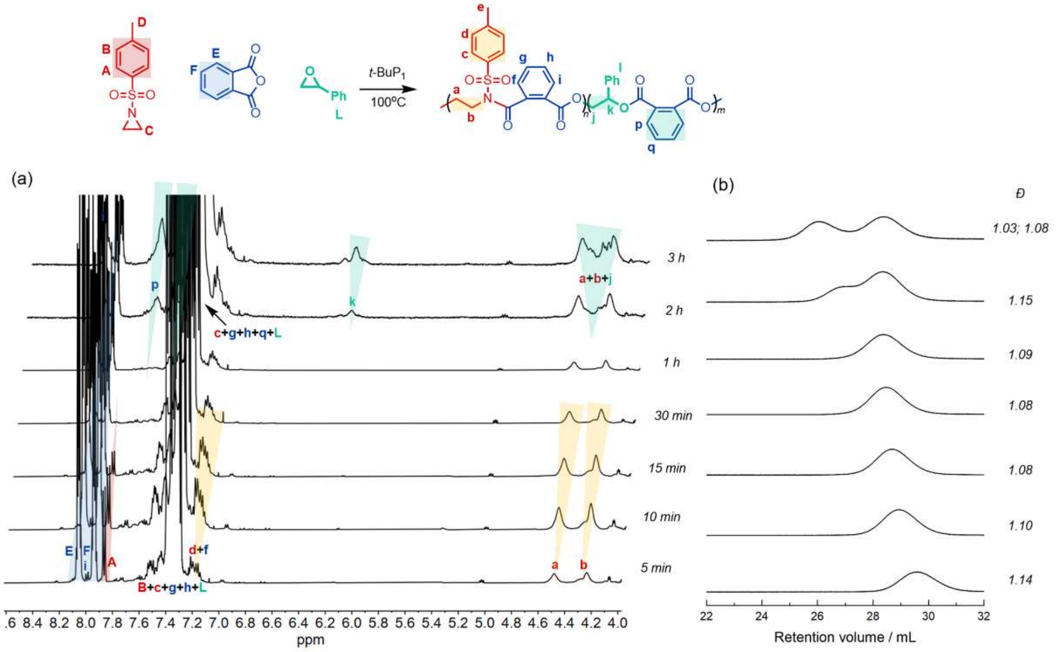

Supplementary Figure 26. Stacked ^1H NMR spectra (400 MHz, $CDCl_3$, 25°C) and SEC traces (THF, 35°C) of the reaction mixture at a ratio of $[TAz]_0/[PA]_0/[BnN(H)Ts]_0/[t\text{-BuP}_1]_0$ = 30/90/1/1 ($[TAz]_0$ = 1.0 M in SO) at 100°C.

Supplementary Table 11. Terpolymerizations of TAz, PA, and EPP using *t*-BuP_1 as catalyst[a]

Entry	Time	Conv. (TAz)[b]/%	Conv.(PA)[b] /%	$M_{n,theo}$[c]/kg mol^{-1}	$M_{n,SEC}$[d]/kg mol^{-1}	$Đ$[d]
1	5 min				6.37	1.06
2	10 min				6.68	1.06
3	15 min				7.08	1.06
4	30 min	99		10.5	8.18	1.12
5	1 h	99			16.7; 7.00	1.03; 1.06
6	2 h	99	99	28.4	19.9; 7.76	1.06; 1.05

[a]The terpolymerizations were performed at a ratio of $[TAz]_0/[PA]_0/[Bn(Ts)NH]_0/[t\text{-}BuP_1]_0$ = 30/90/1/1 ($[TAz]_0$ = 1.0 M in EPP) at 100°C. [b]Determined by ^1H NMR in $CDCl_3$. [c]Calculated as follows: (M.W. of Initiator) + ($[TAz]_0/[I]_0$) × conv.(TAz) × (M.W. of TAz + M.W. of PA) + {($[PA]_0/[I]_0$) × conv.(PA) - ($[TAz]_0/[I]_0$) × conv.(TAz)} × (M.W. of epoxides + M.W. of PA). [d]Determined by SEC at 35°C in THF (1.0 mL min^{-1}) using PSt standards.

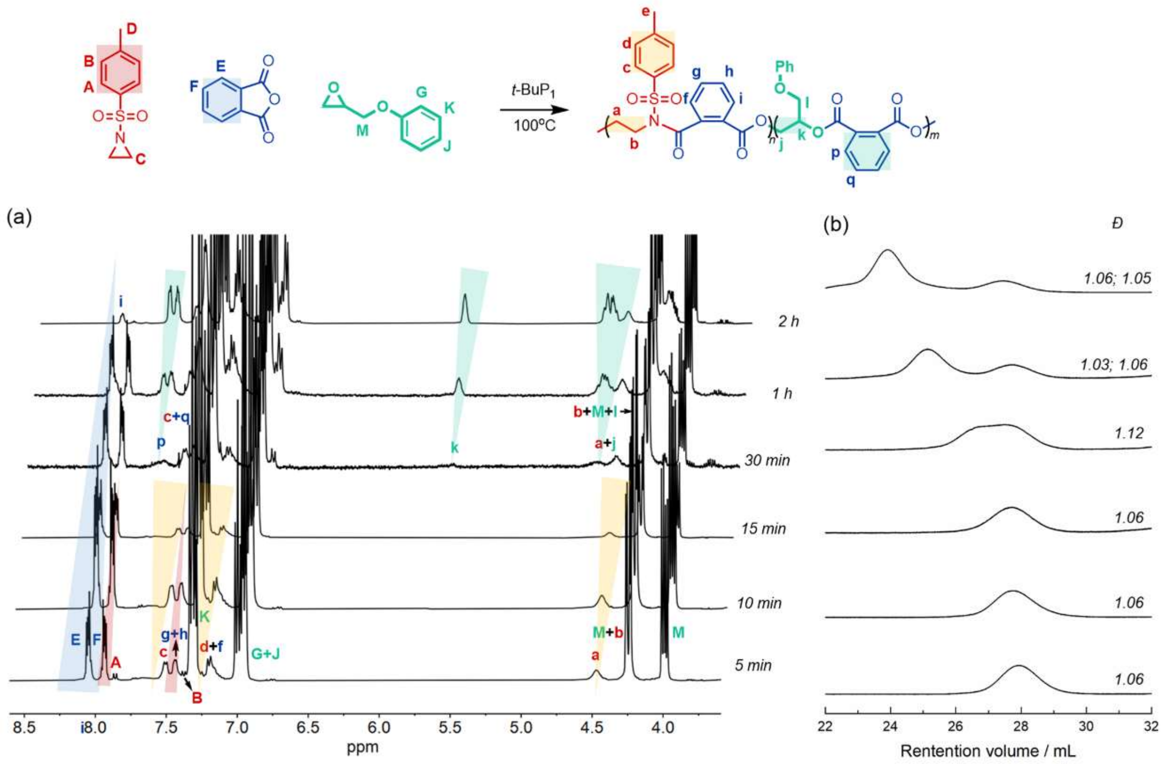

Supplementary Figure 27. Stacked ^1H NMR spectra (400 MHz, $CDCl_3$, 25°C) and SEC traces (THF, 35°C) of the reaction mixture at a ratio of $[TAz]_0/[PA]_0/[BnN(H)Ts]_0/[t\text{-}BuP_1]_0$ = 30/90/1/1 ($[TAz]_0$ = 1.0 M in EPP) at 100°C.

Supplementary Table 12. Terpolymerizations of TAz, PA, and NBGE using t-BuP$_1$ as catalyst[a]

Entry	Time	Conv. (TAz)[b]/%	Conv.(PA)[b] /%	$M_{n,theo}$[c]/kg mol^{-1}	$M_{n,SEC}$[d]/kg mol^{-1}	$Đ$[d]
1	5 min				5.15	1.08
2	10 min				5.52	1.08
3	15 min				5.65	1.08
4	30 min				5.89	1.08
5	1 h	99		10.5	7.00	1.14
6	2 h	99			7.44	1.08; 1.06
7	3 h	99			16.3; 6.28	1.11; 1.08
8	4 h	99	99	27.2	19.5; 6.34	1.16; 1.07

[a]The terpolymerizations were performed at a ratio of $[TAz]_0/[PA]_0/[Bn(Ts)NH]_0/[t\text{-}BuP_1]_0$ = 30/90/1/1 ($[TAz]_0$ = 1.0 M in NBGE) at 100°C. [b]Determined by ^{1}H NMR in $CDCl_3$. [c]Calculated as follows: (M.W. of Initiator) + ($[TAz]_0/[I]_0$) × conv.(TAz) × (M.W. of TAz + M.W. of PA) + {($[PA]_0/[I]_0$) × conv.(PA) - ($[TAz]_0/[I]_0$) × conv.(TAz)} × (M.W. of epoxides + M.W. of PA). [d]Determined by SEC at 35°C in THF (1.0 mL min^{-1}) using PSt standards.

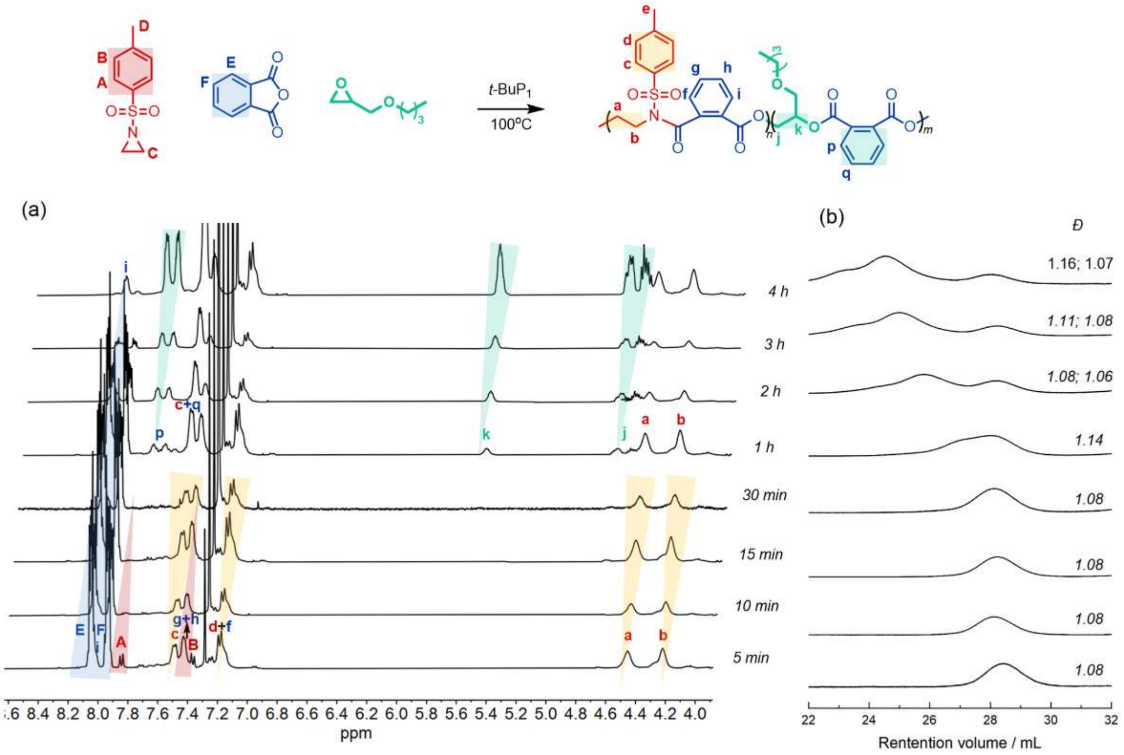

Supplementary Figure 28. Stacked ^{1}H NMR spectra (400 MHz, $CDCl_3$, 25°C) and SEC traces (THF, 35°C) of the reaction mixture at a ratio of $[TAz]_0/[PA]_0/[BnN(H)Ts]_0/[t\text{-}BuP_1]_0$ = 30/90/1/1 ($[TAz]_0$ = 1.0 M in NBGE) at 100°C.

Supplementary Table 13. Terpolymerizations of TAz, PA, and CHO using *t*-BuP$_1$ as catalyst[a]

Entry	Time	Conv. (TAz)[b]/%	Conv.(PA)[b] /%	$M_{n,theo}$[c]/kg mol^{-1}	$M_{n,SEC}$[d]/kg mol^{-1}	$Đ$[d]
1	5 min				2.12	1.19
2	10 min				2.84	1.14
3	15 min				3.07	1.14
4	30 min	99		10.5	3.21	1.14
5	1 h	99			3.08	1.15
6	2 h	99			3.15	1.17
7	3 h	99			3.29	1.25
8	5 h	99	99	25.3	14.5; 3.36	1.10; 1.15

[a]The terpolymerizations were performed at a ratio of $[TAz]_0/[PA]_0/[Bn(Ts)NH]_0/[t\text{-BuP}_1]_0$ = 30/90/1/1 ($[TAz]_0$ = 1.0 M in CHO) at 100°C. [b]Determined by ^{1}H NMR in $CDCl_3$. [c]Calculated as follows: (M.W. of Initiator) + ($[TAz]_0/[I]_0$) × conv.(TAz) × (M.W. of TAz + M.W. of PA) + {($[PA]_0/[I]_0$) × conv.(PA) - ($[TAz]_0/[I]_0$) × conv.(TAz)} × (M.W. of epoxides + M.W. of PA). [d]Determined by SEC at 35°C in THF (1.0 mL min^{-1}) using PSt standards.

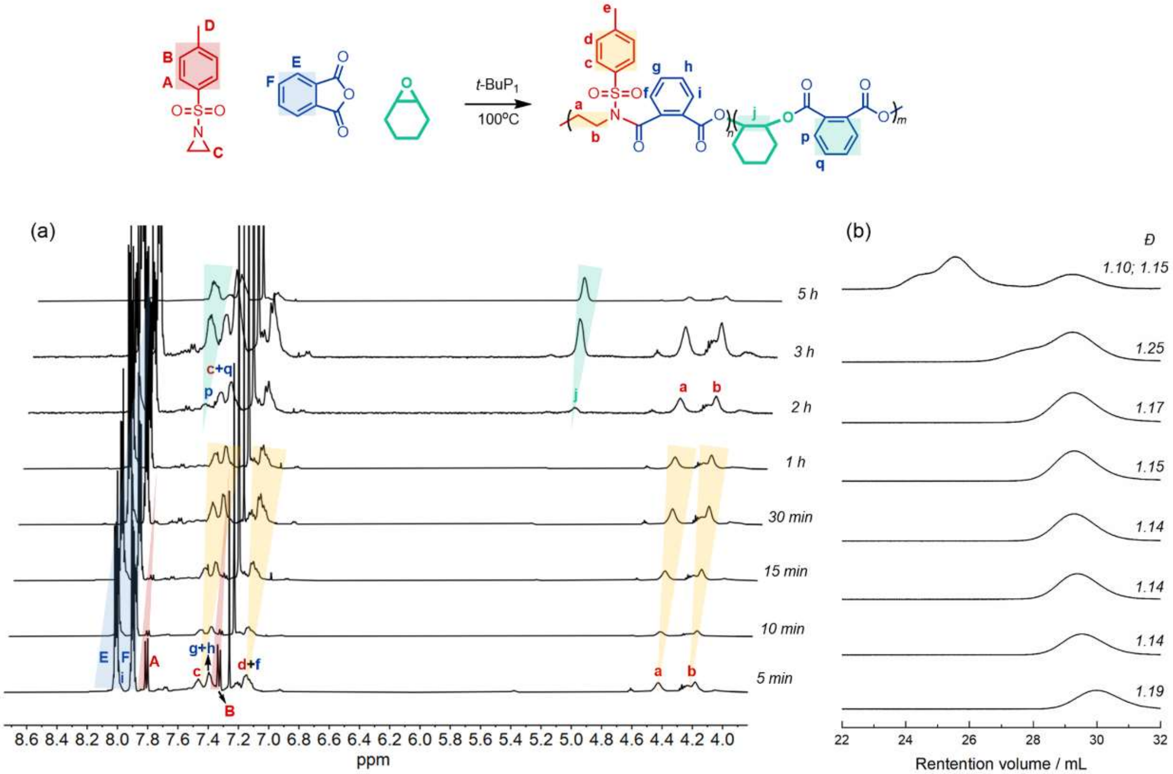

Supplementary Figure 29. Stacked ^{1}H NMR spectra (400 MHz, $CDCl_3$, 25°C) and SEC traces (THF, 35°C) of the reaction mixture at a ratio of $[TAz]_0/[PA]_0/[BnN(H)Ts]_0/[t\text{-BuP}_1]_0$ = 30/90/1/1 ($[TAz]_0$ = 1.0 M in CHO) at 100°C.

Supplementary Note 7. Terpolymerizations of PA, and PO with other *N*-sulfonyl aziridines

Supplementary Table 14. Terpolymerizations of BAz, PA, and PO using *t*-BuP$_1$ as catalyst[a]

Entry	Time	Conv. (BAz)[b]/%	Conv.(PA)[b] /%	$M_{n,theo}$[c]/kg mol^{-1}	$M_{n,NMR}$[b]/kg mol^{-1}	$Đ$[d]
1	2 min	77	26	9.74	9.28	1.06
2	5 min	99	33	12.5	11.7	1.05
3	10 min	99	33	12.5	11.7	1.05
4	15 min	99	33	12.5	11.7	1.06
5	30 min	99	33	12.5	11.7	1.08
6	1 h	99	46	14.9	14.8	1.09
7	2 h	99	56	16.8	16.9	1.10
8	3 h	99	75	20.3	20.7	1.12
9	4 h	99	91	23.3	23.6	1.11
10	5 h	99	99	24.7	25.1	1.10

[a]The terpolymerizations were performed at a ratio of [BAz]$_0$/[PA]$_0$/[BnN(H)Ts]$_0$/[*t*-BuP$_1$]$_0$ = 30/90/1/1 ([BAz]$_0$ = 1.0 M in PO) at 100°C. [b]Determined by ^{1}H NMR in $CDCl_3$ using integrals of the characteristic signals. [c]Calculated as follows: (M.W. of Initiator) + ([BAz]$_0$/[I]$_0$) × conv.(BAz) × (M.W. of BAz + M.W. of PA) + {([PA]$_0$/[I]$_0$) × conv.(PA) - ([BAz]$_0$/[I]$_0$) × conv.(BAz)} × (M.W. of epoxides + M.W. of PA). [d]Determined by SEC at 35°C in THF (1.0 mL min^{-1}) using PSt standards.

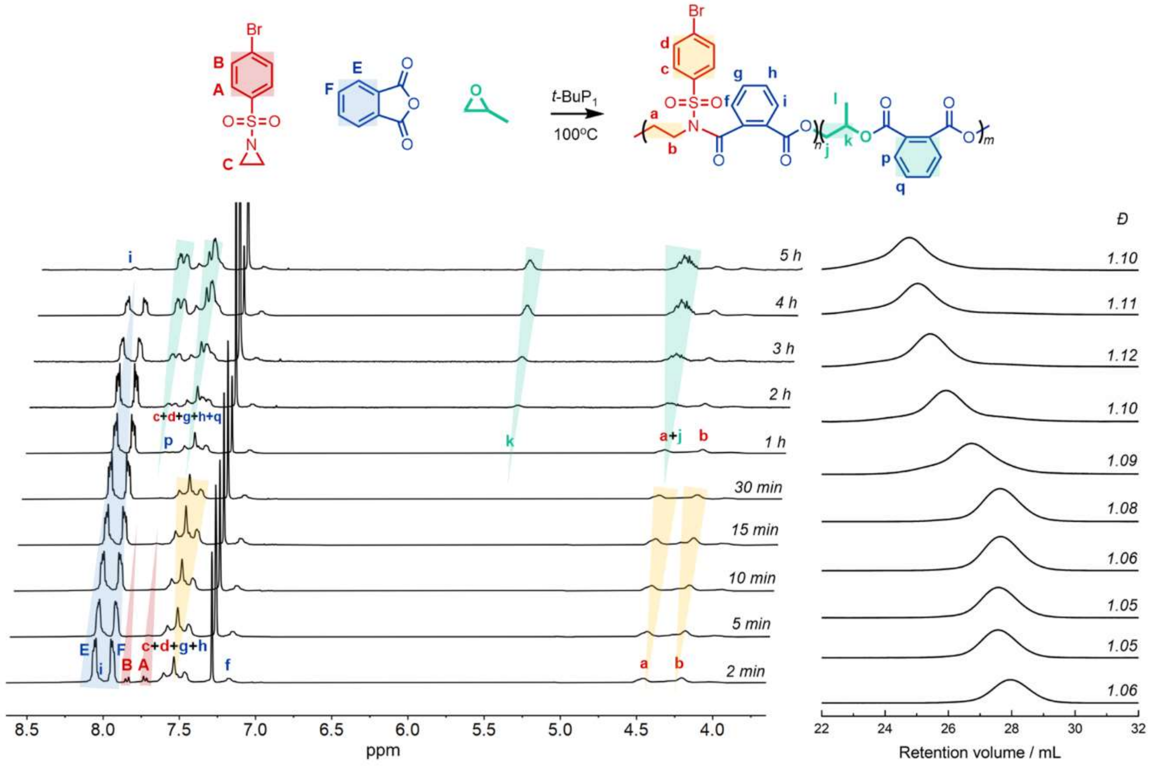

Supplementary Figure 30. Stacked ^{1}H NMR spectra (400 MHz, $CDCl_3$, 25°C) and SEC traces (THF, 35°C) of the reaction mixture at a ratio of [BAz]$_0$/[PA]$_0$/[BnN(H)Ts]$_0$/[*t*-BuP$_1$]$_0$ = 30/90/1/1 ([BAz]$_0$ = 1.0 M in PO) at 100°C.

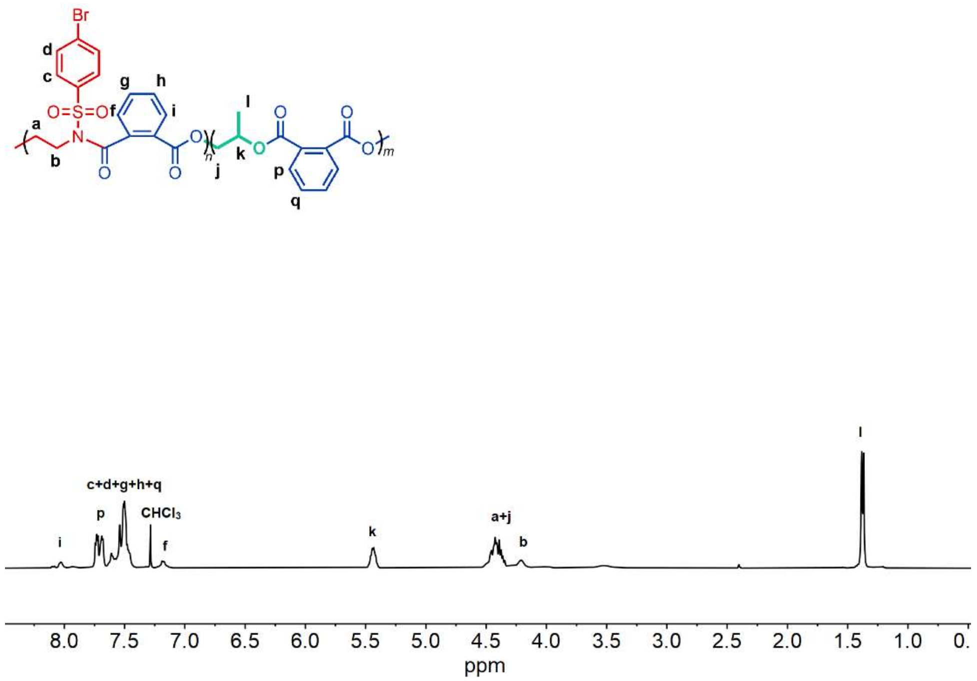

Supplementary Figure 31. ^{1}H NMR spectrum (400 MHz, $CDCl_3$, 25°C) of poly(BAz-*alt*-PA)-*b*-poly(PA-*alt*-PO).

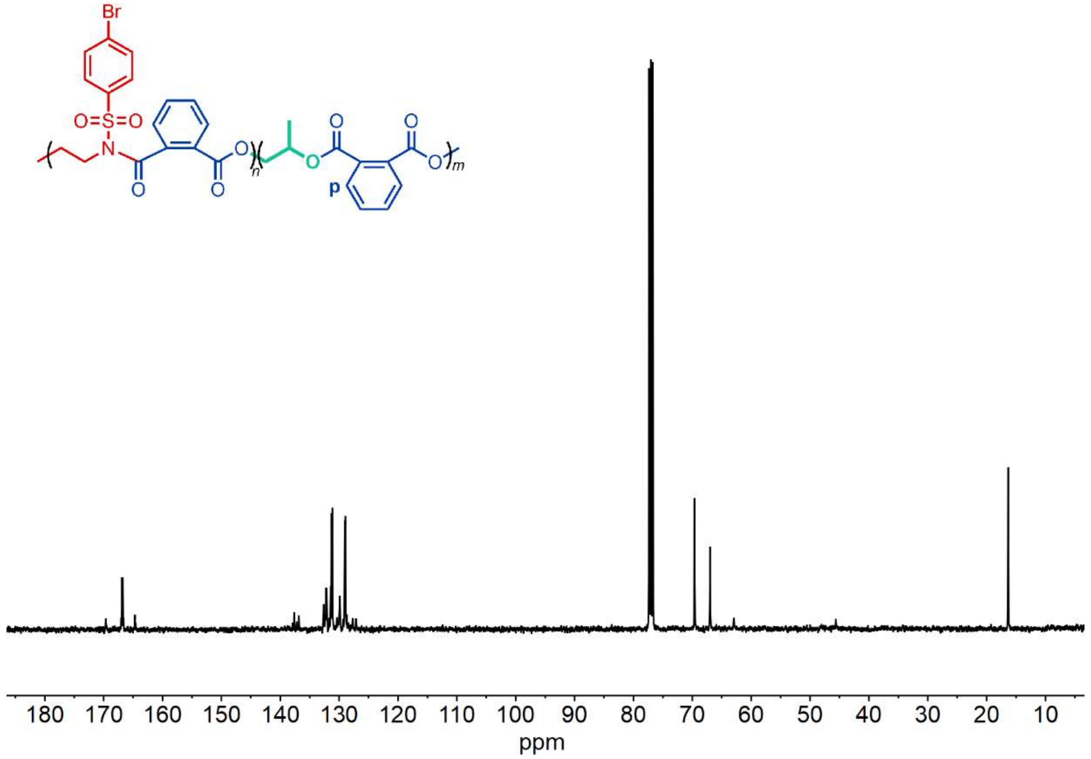

Supplementary Figure 32. ^{13}C NMR spectrum (100 MHz, $CDCl_3$, 25°C) of poly(BAz-*alt*-PA)-*b*-poly(PA-*alt*-PO).

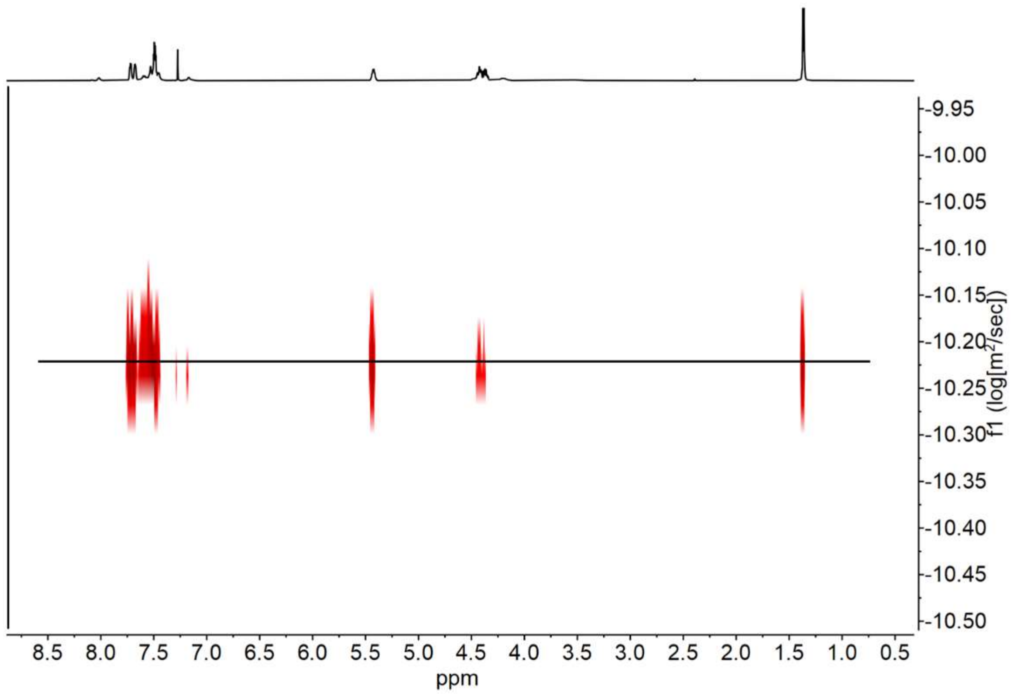

Supplementary Figure 33. DOSY spectrum (600 MHz, $CDCl_3$, 25°C) of poly(BAz-*alt*-PA)-*b*-poly(PA-*alt*-PO).

Supplementary Table 15. Terpolymerizations of NAz, PA, and PO using *t*-BuP$_1$ as catalyst[a]

Entry	Time	Conv. (NAz)[b]/%	Conv.(PA)[b] /%	$M_{n,theo}$[c]/kg mol^{-1}	$M_{n,SEC}$[d]/kg mol^{-1}	$Đ$[d]
1	2 min	99		11.6	7.79	1.08
2	5 min	99			7.79	1.06
3	10 min	99			7.79	1.06
4	15 min	99			7.79	1.05
5	30 min	99			7.79	1.06
6	1 h	99			9.36	1.09
7	2 h	99			16.8	1.09
8	3 h	99			20.9	1.14
9	4 h	99	99	23.7	20.2	1.12
10	5 h	99	99	23.7	20.2	1.11

[a]The terpolymerizations were performed at a ratio of $[NAz]_0/[PA]_0/[BnN(H)Ts]_0/[t\text{-}BuP_1]_0$ = 30/90/1/1 ($[NAz]_0$ = 1.0 M in PO) at 100°C. [b]Determined by ^1H NMR in $CDCl_3$ using integrals of the characteristic signals. [c]Calculated as follows: (M.W. of Initiator) + ($[NAz]_0/[I]_0$) × conv.(NAz) × (M.W. of NAz + M.W. of PA) + {($[PA]_0/[I]_0$) × conv.(PA) - ($[NAz]_0/[I]_0$) × conv.(NAz)} × (M.W. of epoxides + M.W. of PA). [d]Determined by SEC at 35°C in THF (1.0 mL min^{-1}) using PSt standards.

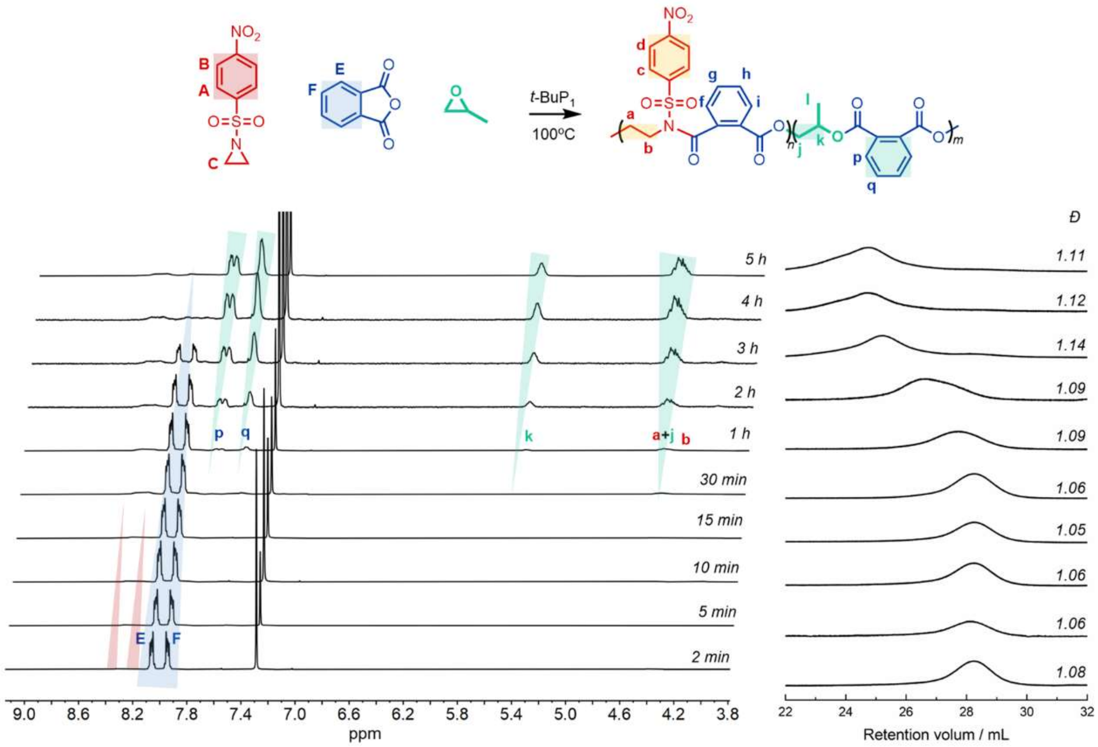

Supplementary Figure 34. Stacked ^{1}H NMR spectra (400 MHz, $CDCl_3$, 25°C) and SEC traces (THF, 35°C) of the reaction mixture at a ratio of $[NAz]_0/[PA]_0/[BnN(H)Ts]_0/[t\text{-}BuP_1]_0$ = 30/90/1/1 ($[NAz]_0$ = 1.0 M in PO) at 100°C. (The low solubility of poly(NAz-*alt*-PA) leads to low NMR signal)

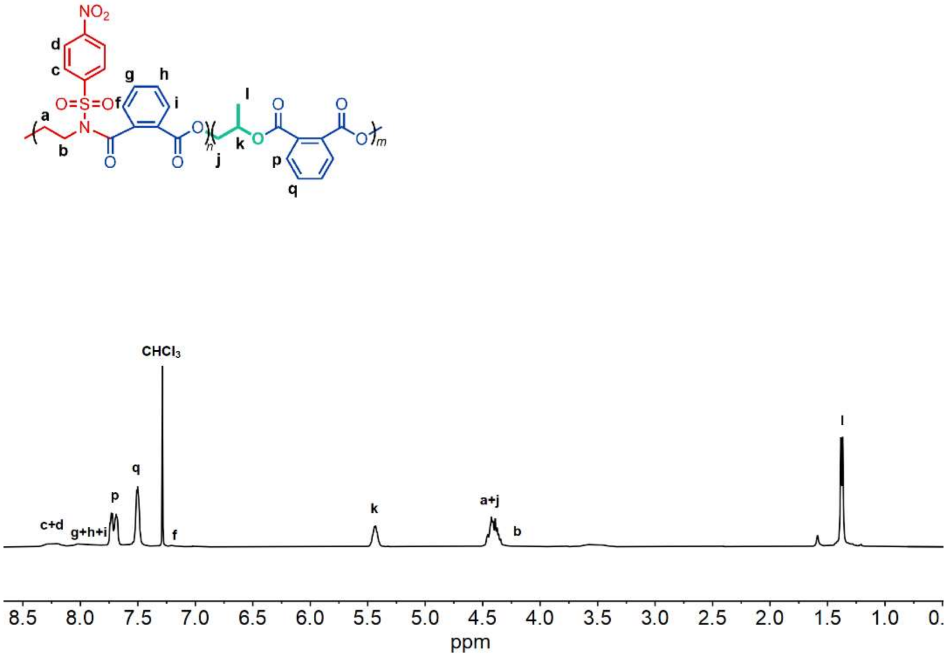

Supplementary Figure 35. ^{1}H NMR spectrum (400 MHz, $CDCl_3$, 25°C) of poly(NAz-*alt*-PA)-*b*-poly(PA-*alt*-PO).

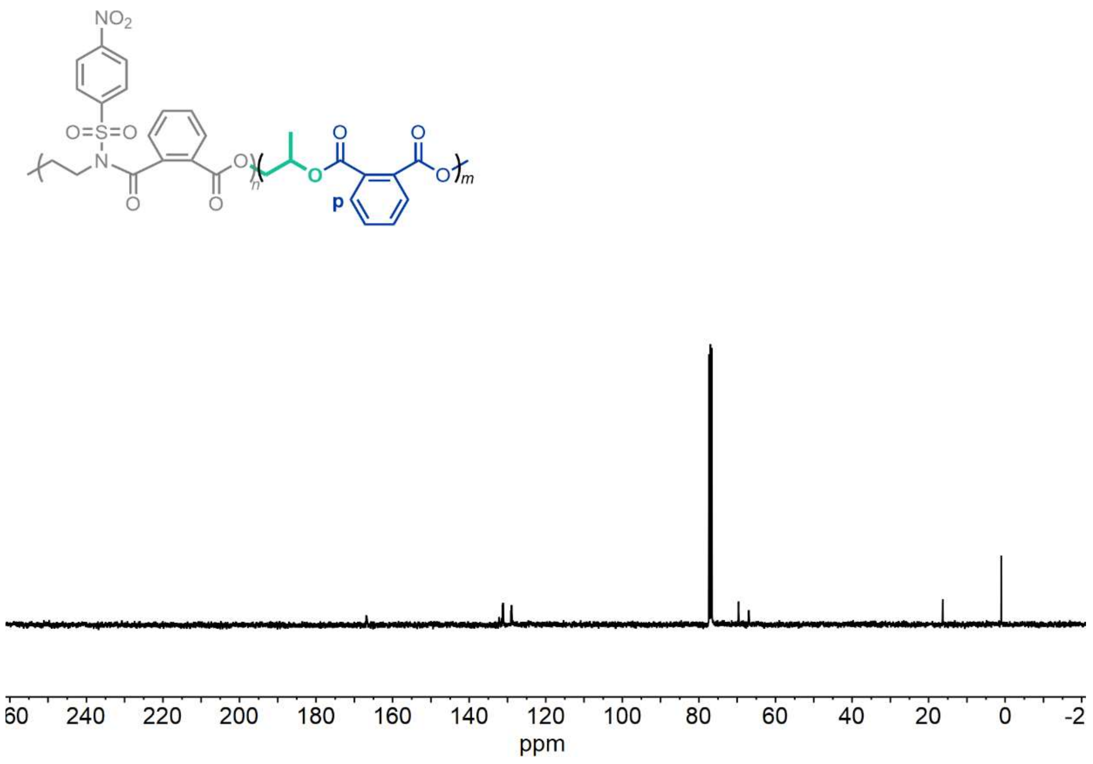

Supplementary Figure 36. ^{13}C NMR spectrum (100 MHz, $CDCl_3$, 25°C) of poly(NAz-*alt*-PA)-*b*-poly(PA-*alt*-PO). (The low solubility of poly(NAz-*alt*-PA) leads to low NMR signal)

Supplementary Note 8. Terpolymerizations of TAz, PA, and PO with different bases catalysts

Supplementary Table 16. Terpolymerizations of TAz, PA, and PO using *t*-BuP$_4$ as catalyst[a]

Entry	Time	Conv. (TAz)[b]/%	Conv.(PA)[b] /%	$M_{n,theo}$[c]/kg mol^{-1}	$M_{n,NMR}$[b]/kg mol^{-1}	*Đ*[d]
1	2 min	84	28	8.96	9.13	1.06
2	5 min	98	32	10.4	10.8	1.03
3	10 min	99	33	10.5	11.2	1.03
4	15 min	99	33	10.5	11.2	1.03
5	30 min	99	33	10.5	11.2	1.06
6	1 h	99	46	13.0	14.1	1.13
7	2 h	99	83	19.8	21.3	1.20
8	3 h	99	99	22.8	25.6	1.77

[a]The terpolymerizations were performed at a ratio of $[TAz]_0/[PA]_0/[BnN(H)Ts]_0/[t\text{-}BuP_4]_0$ = 30/90/1/1 ($[TAz]_0$ = 1.0 M in PO) at 100°C. [b]Determined by ^{1}H NMR in $CDCl_3$ using integrals of the characteristic signals. [c]Calculated as follows: (M.W. of Initiator) + ($[TAz]_0/[I]_0$) × conv.(TAz) × (M.W. of TAz + M.W. of PA) + {($[PA]_0/[I]_0$) × conv.(PA) - ($[TAz]_0/[I]_0$) × conv.(TAz)} × (M.W. of epoxides + M.W. of PA). [d]Determined by SEC at 35°C in THF (1.0 mL min^{-1}) using PSt standards.

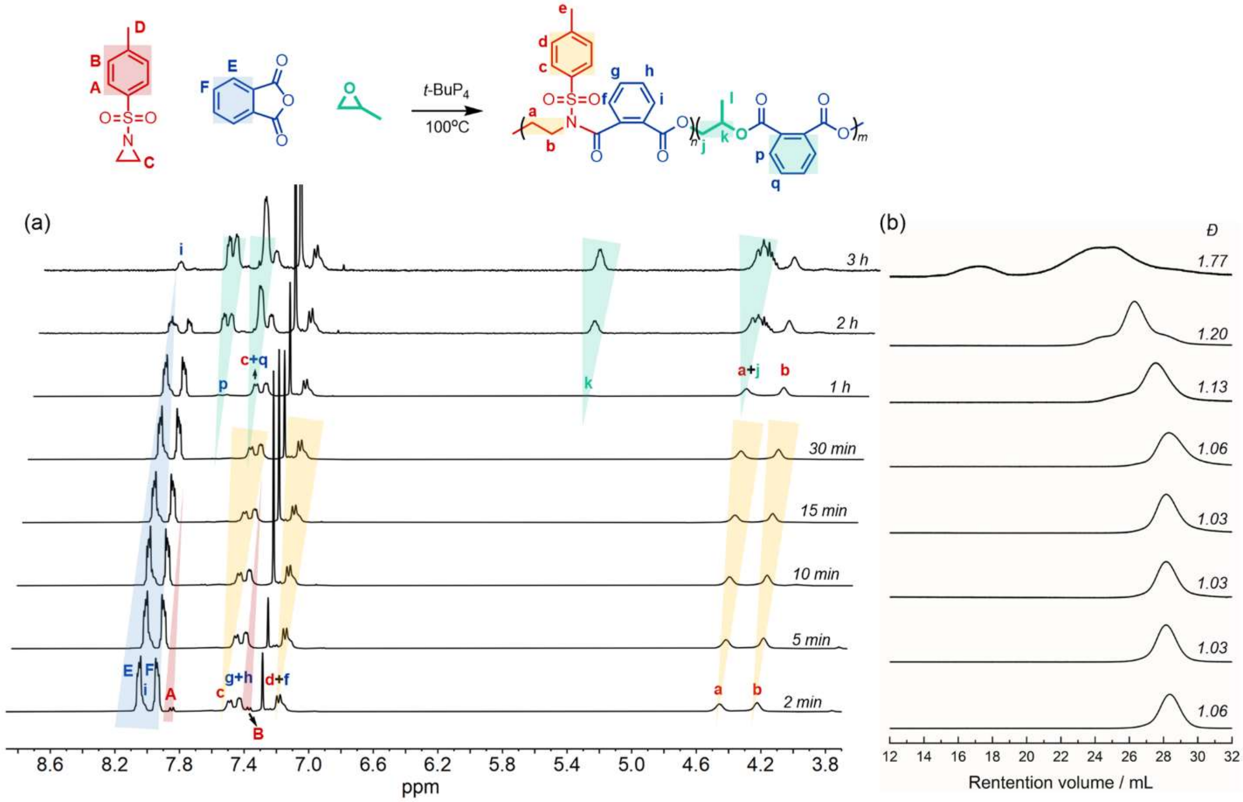

Supplementary Figure 37. Stacked ^{1}H NMR spectra (400 MHz, $CDCl_3$, 25°C) and SEC traces (THF, 35°C) of the reaction mixture at the ratio of $[TAz]_0/[PA]_0/[BnN(H)Ts]_0/[t\text{-}BuP_4]_0$ = 30/90/1/1 ($[TAz]_0$ = 1.0 M in PO) at 100°C (entries 22 and 23, Table 1).

Supplementary Table 17. Terpolymerizations of TAz, PA, and PO using *t*-BuP$_2$ as catalyst[a]

Entry	Time	Conv. (TAz)[b]/%	Conv.(PA)[b] /%	$M_{n,theo}$[c]/kg mol^{-1}	$M_{n,NMR}$[b]/kg mol^{-1}	*Đ*[d]
1	2 min	77	25	8.24	8.39	1.07
2	5 min	97	32	10.3	10.7	1.04
3	10 min	99	33	10.5	11.2	1.03
4	15 min	99	33	10.5	11.2	1.06
5	30 min	99	33	10.5	11.2	1.08
6	1 h	99	45	12.8	14.1	1.14
7	2 h	99	67	16.9	17.6	1.16
8	3 h	99	89	20.9	22.1	1.18
9	4 h	99	99	22.8	24.8	1.25

[a]The terpolymerizations were performed at a ratio of $[TAz]_0/[PA]_0/[BnN(H)Ts]_0/[t\text{-}BuP_2]_0$ = 30/90/1/1 ($[TAz]_0$ = 1.0 M in PO) at 100°C. [b]Determined by ^{1}H NMR in $CDCl_3$ using integrals of the characteristic signals. [c]Calculated as follows: (M.W. of Initiator) + ($[TAz]_0/[I]_0$) × conv.(TAz) × (M.W. of TAz + M.W. of PA) + {($[PA]_0/[I]_0$) × conv.(PA) - ($[TAz]_0/[I]_0$) × conv.(TAz)} × (M.W. of epoxides + M.W. of PA). [d]Determined by SEC at 35°C in THF (1.0 mL min^{-1}) using PSt standards.

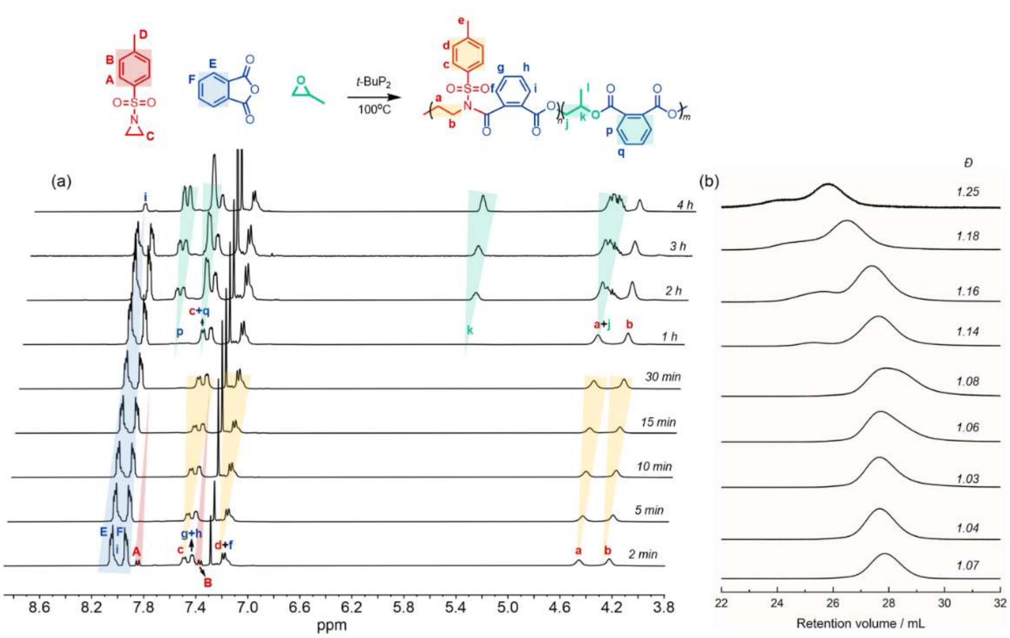

Supplementary Figure 38. Stacked ^{1}H NMR spectra (400 MHz, $CDCl_3$, 25°C) and SEC traces (THF, 35°C) of the reaction mixture at the ratio of $[TAz]_0/[PA]_0/[BnN(H)Ts]_0/[t\text{-}BuP_2]_0$ = 30/90/1/1 ($[TAz]_0$ = 1.0 M in PO) at 100°C (entries 24 and 25, Table 1).

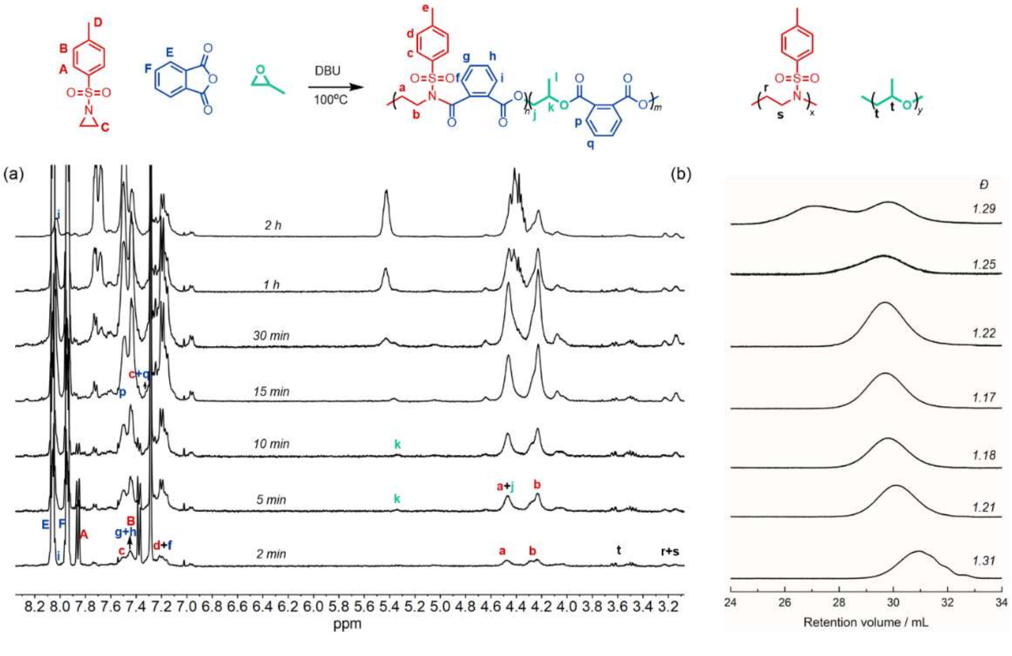

Supplementary Figure 39. Stacked ^{1}H NMR spectra (400 MHz, $CDCl_3$, 25°C) and SEC traces (THF, 35°C) of the reaction mixture at the ratio of $[TAz]_0/[PA]_0/[BnN(H)Ts]_0/[DBU]_0$ = 30/90/1/1 ($[TAz]_0$ = 1.0 M in PO) at 100°C (entry 26, Table 1).

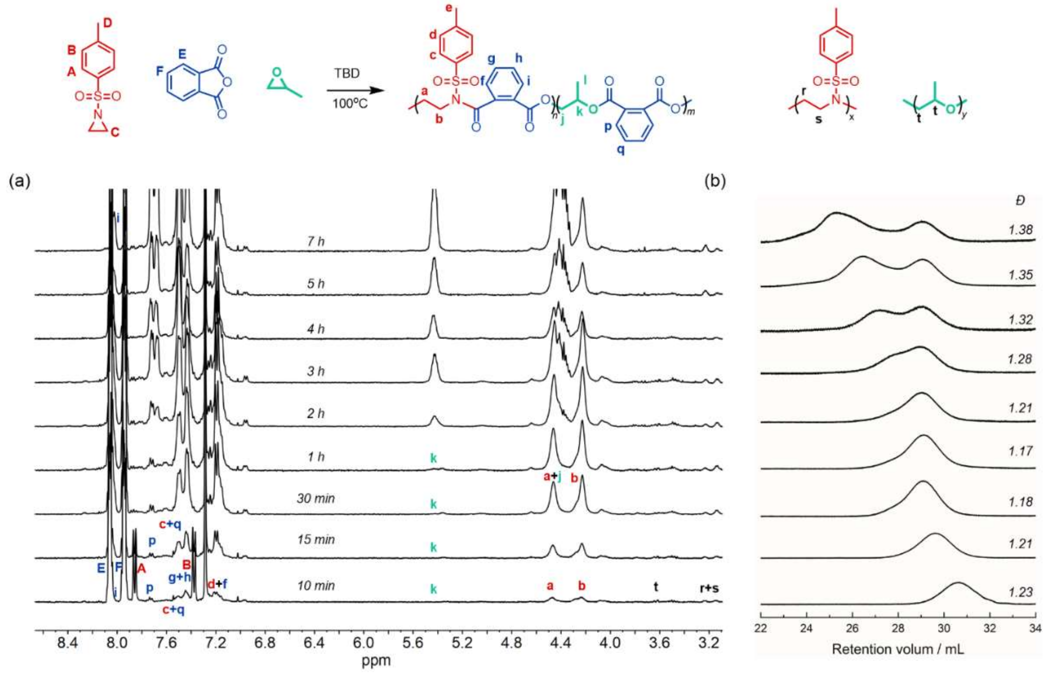

Supplementary Figure 40. Stacked ^{1}H NMR spectra (400 MHz, $CDCl_3$, 25°C) and SEC traces (THF, 35°C) of the reaction mixture at the ratio of $[TAz]_0/[PA]_0/[BnN(H)Ts]_0/[TBD]_0$ = 30/90/1/1 ($[TAz]_0$ = 1.0 M in PO) at 100°C (entry 27, Table 1).

Supplementary Note 9. Terpolymerizations of TAz, PA, and PO with different monomer feed ratios

Supplementary Table 18. Terpolymerizations of TAz, PA, and PO using t-BuP$_1$ as catalyst[a]

Entry	Time	Conv. (TAz)[b]/%	Conv.(PA)[b] /%	$M_{n,theo}$[c]/kg mol^{-1}	$M_{n,NMR}$[b]/kg mol^{-1}	$Đ$[d]
1	2 min	25	12	2.85	2.96	1.07
2	5 min	63	21	6.79	6.98	1.04
3	10 min	94	47	10.0	10.8	1.03
4	15 min	99	49	10.5	11.2	1.03
5	30 min	99	49	10.5	11.2	1.03
6	1 h	99	52	10.9	11.9	1.06
7	2 h	99	69	13.0	13.7	1.04
8	3 h	99	88	15.3	15.9	1.04
9	4 h	99	99	16.7	18.2	1.04

[a]The terpolymerizations were performed at a ratio of $[TAz]_0/[PA]_0/[BnN(H)Ts]_0/[t\text{-}BuP_1]_0$ = 30/60/1/1 ($[TAz]_0$ = 1.0 M in PO) at 100°C. [b]Determined by ^{1}H NMR in $CDCl_3$ using integrals of the characteristic signals. [c]Calculated as follows: (M.W. of Initiator) + ($[TAz]_0/[I]_0$) × conv.(TAz) × (M.W. of TAz + M.W. of PA) + {($[PA]_0/[I]_0$) × conv.(PA) - ($[TAz]_0/[I]_0$) × conv.(TAz)} × (M.W. of epoxides + M.W. of PA). [d]Determined by SEC at 35°C in THF (1.0 mL min^{-1}) using PSt standards.

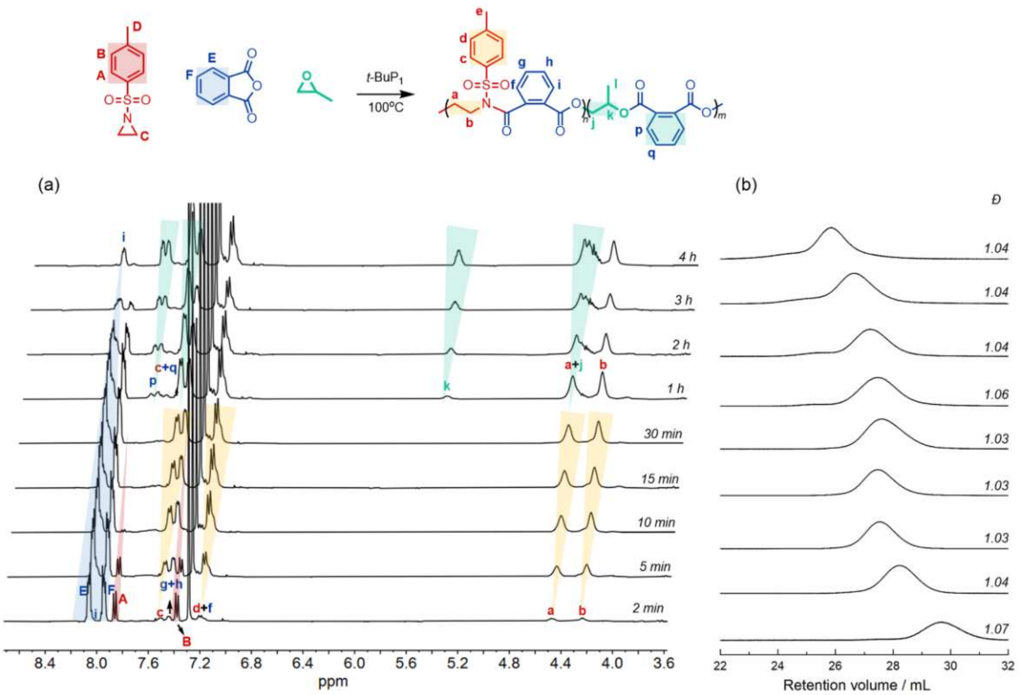

Supplementary Figure 41. Stacked ^{1}H NMR spectra (400 MHz, $CDCl_3$, 25°C) and SEC traces (THF, 35°C) of the reaction mixture at the ratio of $[TAz]_0/[PA]_0/[BnN(H)Ts]_0/[t\text{-}BuP_1]_0$ = 30/60/1/1 ($[TAz]_0$ = 1.0 M in PO) at 100°C (entries 28 and 29, Table 1).

Supplementary Table 19. Terpolymerizations of TAz, PA, and PO using *t*-BuP$_1$ as catalyst[a]

Entry	Time	Conv. (TAz)[b]/%	Conv.(PA)[b] /%	$M_{n,theo}$[c]/kg mol^{-1}	$M_{n,NMR}$[b]/kg mol^{-1}	*Đ*[d]
1	2 min	36	9	4.00	4.31	1.07
2	5 min	77	19	8.24	8.98	1.05
3	10 min	99	24	10.5	11.2	1.03
4	15 min	99	24	10.5	11.2	1.03
5	30 min	99	24	10.5	11.2	1.03
6	1 h	99	30	11.8	13.1	1.06
7	2 h	99	35	13.1	14.2	1.04
8	3 h	99	47	16.0	16.9	1.04
9	4 h	99	58	18.8	20.1	1.04
10	6 h	99	88	26.2	27.8	1.03
11	8 h	99	99	28.9	30.0	1.02

[a]The terpolymerizations were performed at a ratio of $[TAz]_0/[PA]_0/[BnN(H)Ts]_0/[t\text{-}BuP_1]_0$ = 30/120/1/1 ($[TAz]_0$ = 1.0 M in PO) at 100°C. [b]Determined by ^{1}H NMR in $CDCl_3$ using integrals of the characteristic signals. [c]Calculated as follows: (M.W. of Initiator) + ($[TAz]_0/[I]_0$) × conv.(TAz) × (M.W. of TAz + M.W. of PA) + {($[PA]_0/[I]_0$) × conv.(PA) - ($[TAz]_0/[I]_0$) × conv.(TAz)} × (M.W. of epoxides + M.W. of PA). [d]Determined by SEC at 35°C in THF (1.0 mL min^{-1}) using PSt standards.

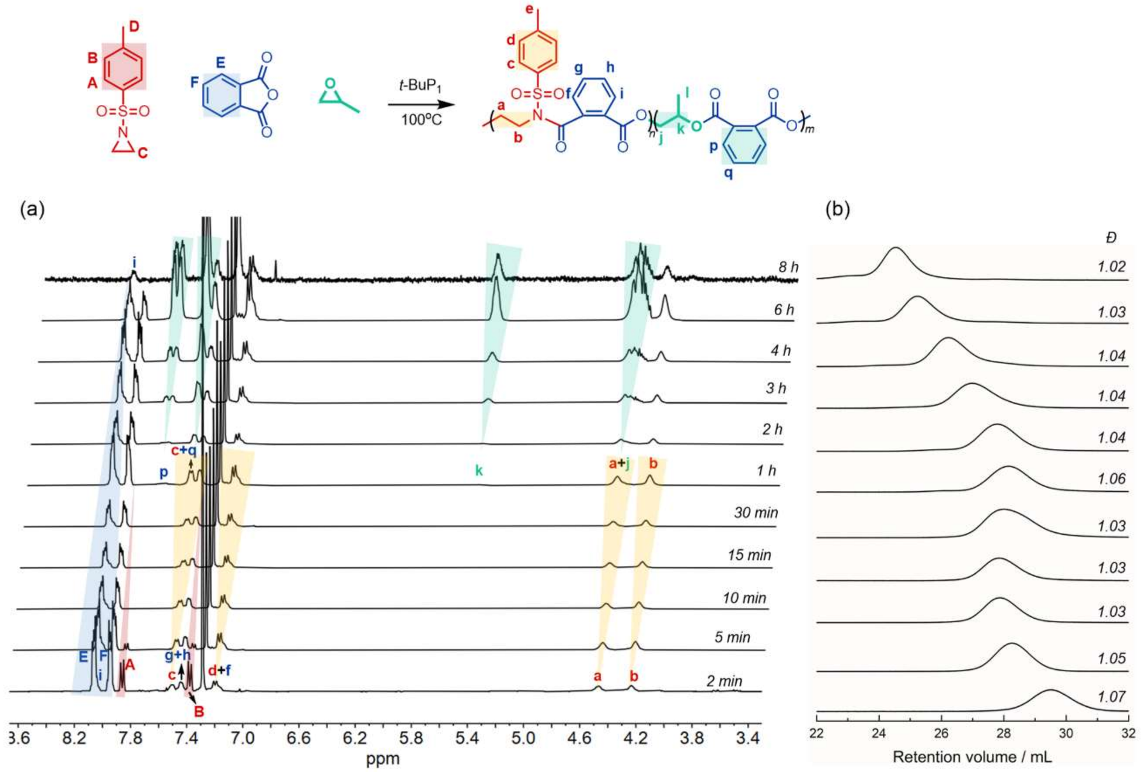

Supplementary Figure 42. Stacked ^{1}H NMR spectra (400 MHz, $CDCl_3$, 25°C) and SEC traces (THF, 35°C) of the reaction mixture at the ratio of $[TAz]_0/[PA]_0/[BnN(H)Ts]_0/[t\text{-}BuP_1]_0$ = 30/120/1/1 ($[TAz]_0$ = 1.0 M in PO) at 100°C (entries 30 and 31, Table 1).

Supplementary Note 10. Multiple switches

Supplementary Table 20. Terpolymerizations of TAz, PA, and PO using *t*-BuP$_1$ as catalyst as catalyst[a]

Entry	Time	Conv. (TAz)[b]/%	Conv.(PA)[b] /%	$M_{n,theo}$[c]/kg mol^{-1}	$M_{n,NMR}$[b]/kg mol^{-1}	$Đ$[d]
1	30 min	98	33	10.4	11.3	1.04
2	2.5 h	99	70	17.4	19.6	1.06
3[e]	3 h	99	80	20.9	22.3	1.06
4[e]	6 h	99	99	24.4	26.5	1.09

[a]The terpolymerizations were performed at the ratio of $[TAz]_0/[PA]_0/[BnN(H)Ts]_0/[t\text{-}BuP_1]_0$ = 30/90/1/1 ($[TAz]_0$ = 1.0 M in epoxides) at 100°C. [b]Determined by ^{1}H NMR in $CDCl_3$ using integrals of the characteristic signals. [c]Calculated as follows: (M.W. of Initiator) + ($[TAz]_0/[I]_0$) × conv.(TAz) × (M.W. of TAz + M.W. of PA) + {($[PA]_0/[I]_0$) × conv.(PA) - ($[TAz]_0/[I]_0$) × conv.(TAz)} × (M.W. of epoxides + M.W. of PA). [d]Determined by SEC at 35°C in THF (1.0 mL min^{-1}) using PSt standards. [e]The addition of extra TAz.

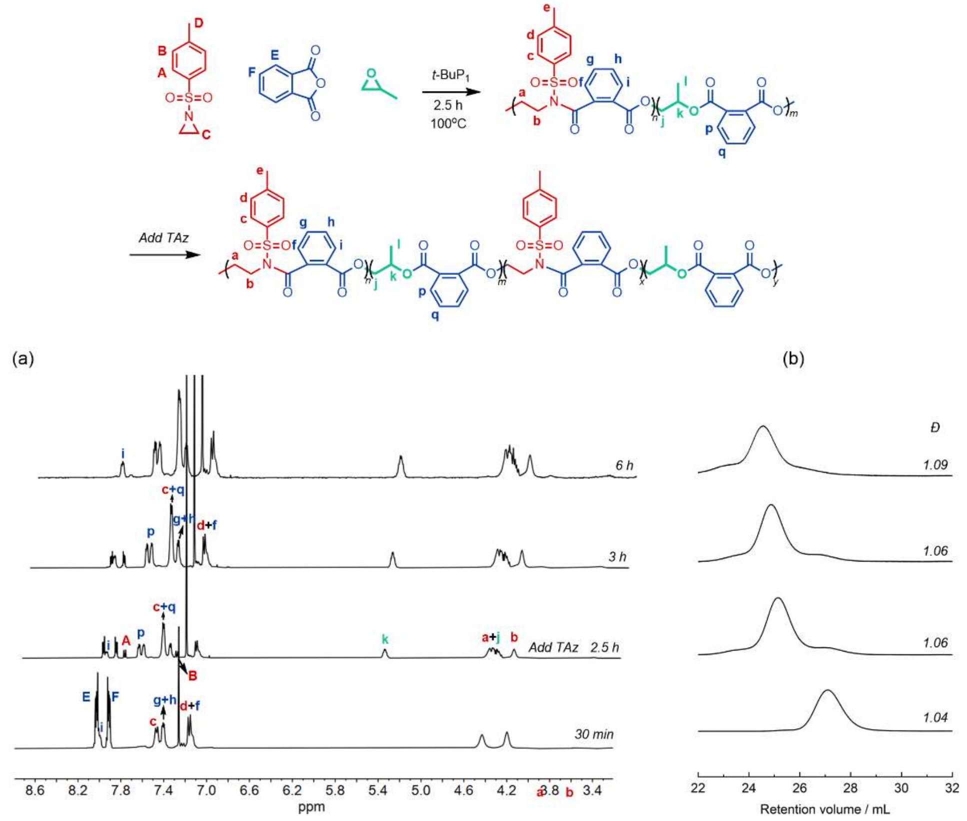

Supplementary Figure 43. Stacked ^{1}H NMR spectra (400 MHz, $CDCl_3$, 25°C) and SEC traces (THF, 35°C) of the crude reaction mixture from terpolymerizations of TAz, PA, and PO at different reaction times. After 2.5 h, TAz was added to the reaction mixture.

Supplementary Table 21. Terpolymerizations of TAz, PA, and PO using t-BuP$_1$ as catalyst[a]

Entry	Time	Conv. (TAz)[b]/%	Conv.(PA)[b] /%	$M_{n,theo}$[c]/kg mol^{-1}	$M_{n,NMR}$[b]/kg mol^{-1}	$Đ$[d]
1	30 min	99	33	10.5	11.2	1.04
2	5 h	99	99	22.8	24.8	1.04
3[e]	5.5 h	99	67	33.3	35.1	1.05
4[e]	10 h	99	99	45.6	49.2	1.18

[a]The terpolymerizations were performed at a ratio of $[TAz]_0/[PA]_0/[BnN(H)Ts]_0/[t\text{-BuP}_1]_0$ = 30/90/1/1 ($[TAz]_0$ = 1.0 M in epoxides) at 100°C. [b]Determined by ^{1}H NMR in $CDCl_3$ using integrals of the characteristic signals. [c]Calculated as follows: (M.W. of Initiator) + ($[TAz]_0/[I]_0$) × conv.(TAz) × (M.W. of TAz + M.W. of PA) + {($[PA]_0/[I]_0$) × conv.(PA) - ($[TAz]_0/[I]_0$) × conv.(TAz)} × (M.W. of epoxides + M.W. of PA). [d]Determined by SEC at 35°C in THF (1.0 mL min^{-1}) using PSt standards. [e]The addition of extra TAz and PA.

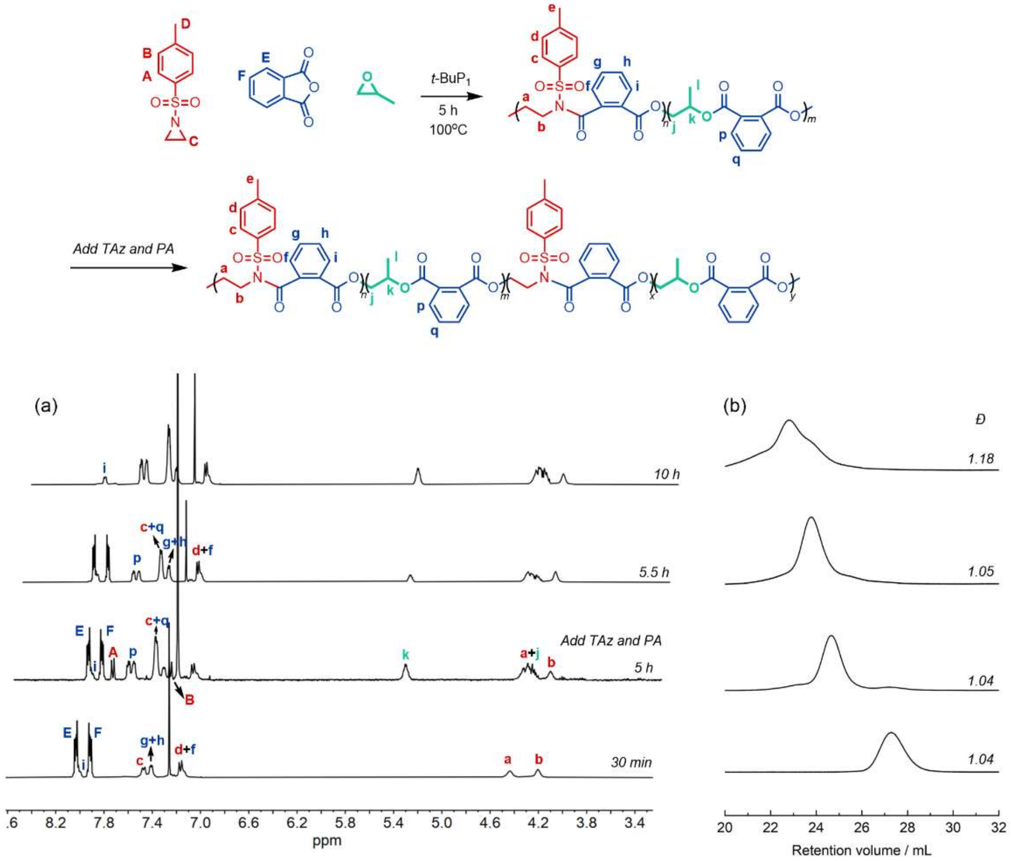

Supplementary Figure 44. Stacked ^{1}H NMR spectra (400 MHz, $CDCl_3$, 25°C) and SEC traces (THF, 35°C) of the crude reaction mixture from terpolymerizations of TAz, PA, and PO at different reaction time. After 5 h, TAz and PA were added to the reaction mixture.

Supplementary Note 11. Kinetic studies

$$-\frac{d[PA]}{dt} = k_{obs}$$

$$-\int d[PA] = k_{obs}\int dt$$

$$[PA]_0 - [PA]_t = k_{obs} \times t$$

$$\frac{[PA]_0 - [PA]_t}{t} = k_{obs}$$

$$\frac{conv.\% \div 100}{t \div [PA]_0} = k_{obs}$$

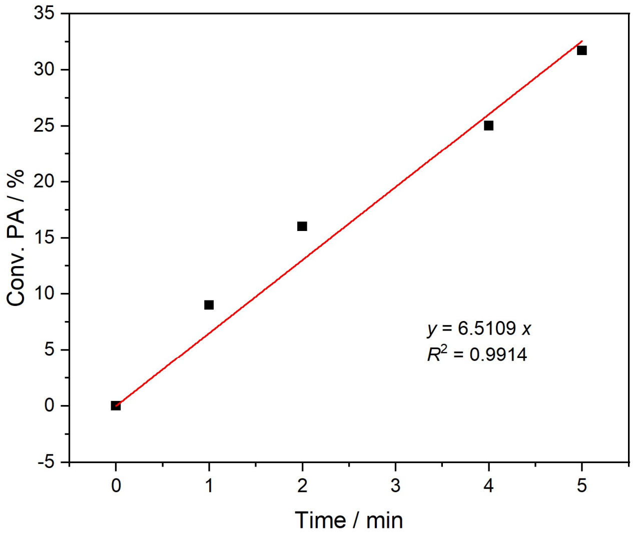

Supplementary Figure 45. Zero-order plot of PA conversion versus time at the first stage copolymerization at 100°C ($[PA]_0$ = 3.0 M) (k_{obs} = 6.5109/100*60*3 = 11.7196 mol L^{-1} h^{-1}).

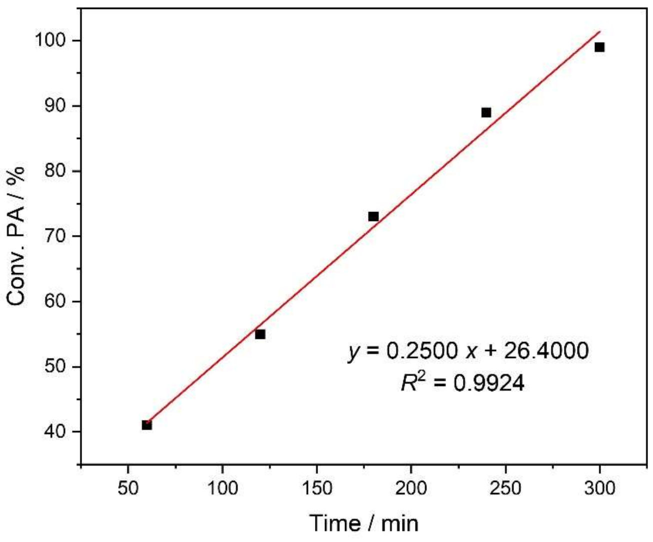

Supplementary Figure 46. Zero-order plot of PA conversion versus time at the second stage copolymerization at 100°C ($[PA]_0$ = 3.0 M) (k_{obs} = 0.2500/100*60*3 = 0.4500 mol L^{-1} h^{-1}).

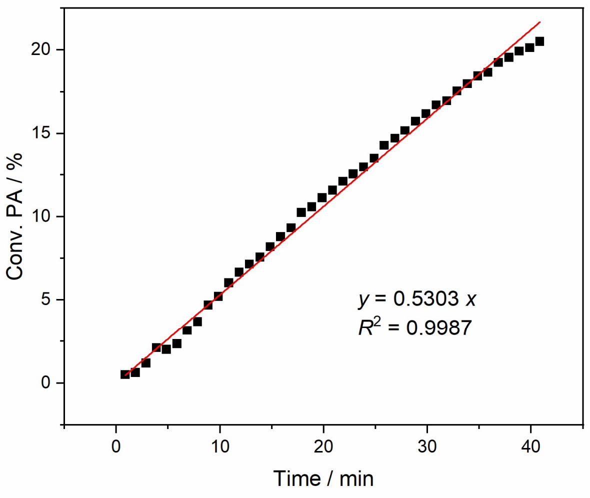

Supplementary Figure 47. Zero-order plot of PA conversion versus time at the first stage copolymerization at 60°C ($[PA]_0$ = 1.5 M) (k_{obs} = 0.5303/100*60*1.5 = 0.4773 mol L^{-1} h^{-1}).

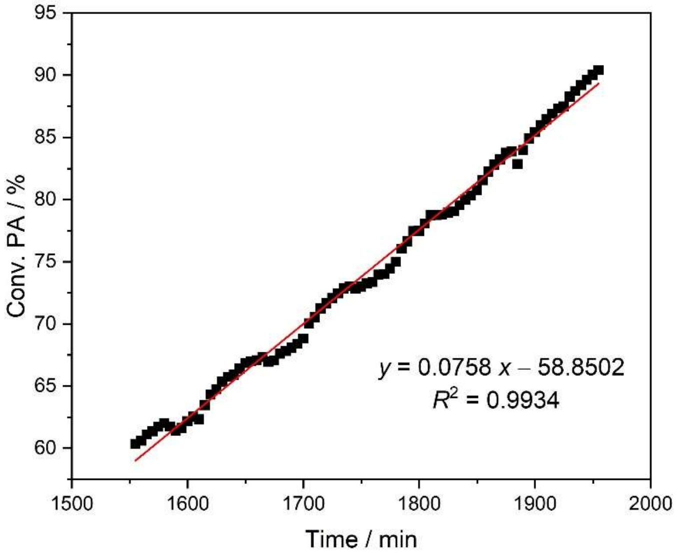

Supplementary Figure 48. Zero-order plot of PA conversion (60-100%) versus time at the second stage copolymerization at 60°C ($[PA]_0$ = 1.5 M) (k_{obs} = 0.0758/100*60*1.5 = 0.0682 mol L^{-1} h^{-1}).

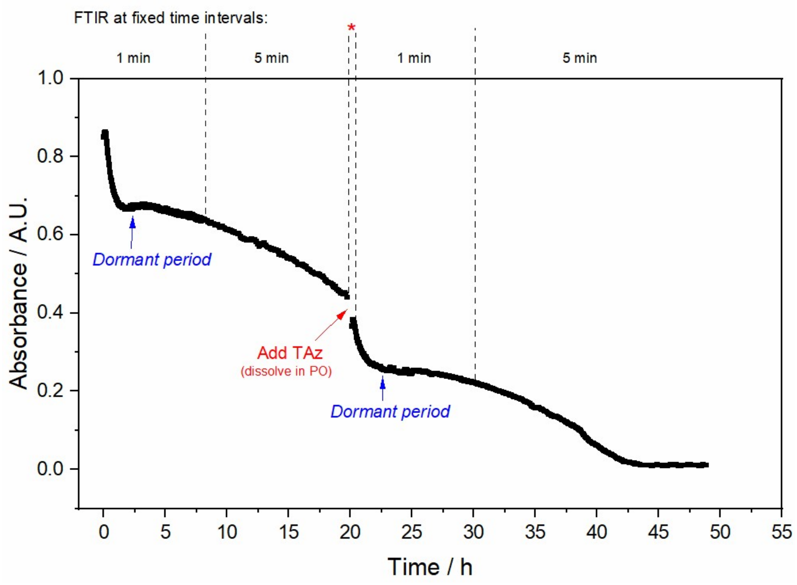

Supplementary Figure 49. In-situ FTIR absorption intensity in the range of 1779 cm^{-1} versus time for the terpolymerization of TAz, PA, and PO (*second feeding).

Supplementary Note 12. Mechanism

12.1 Proposed mechanism

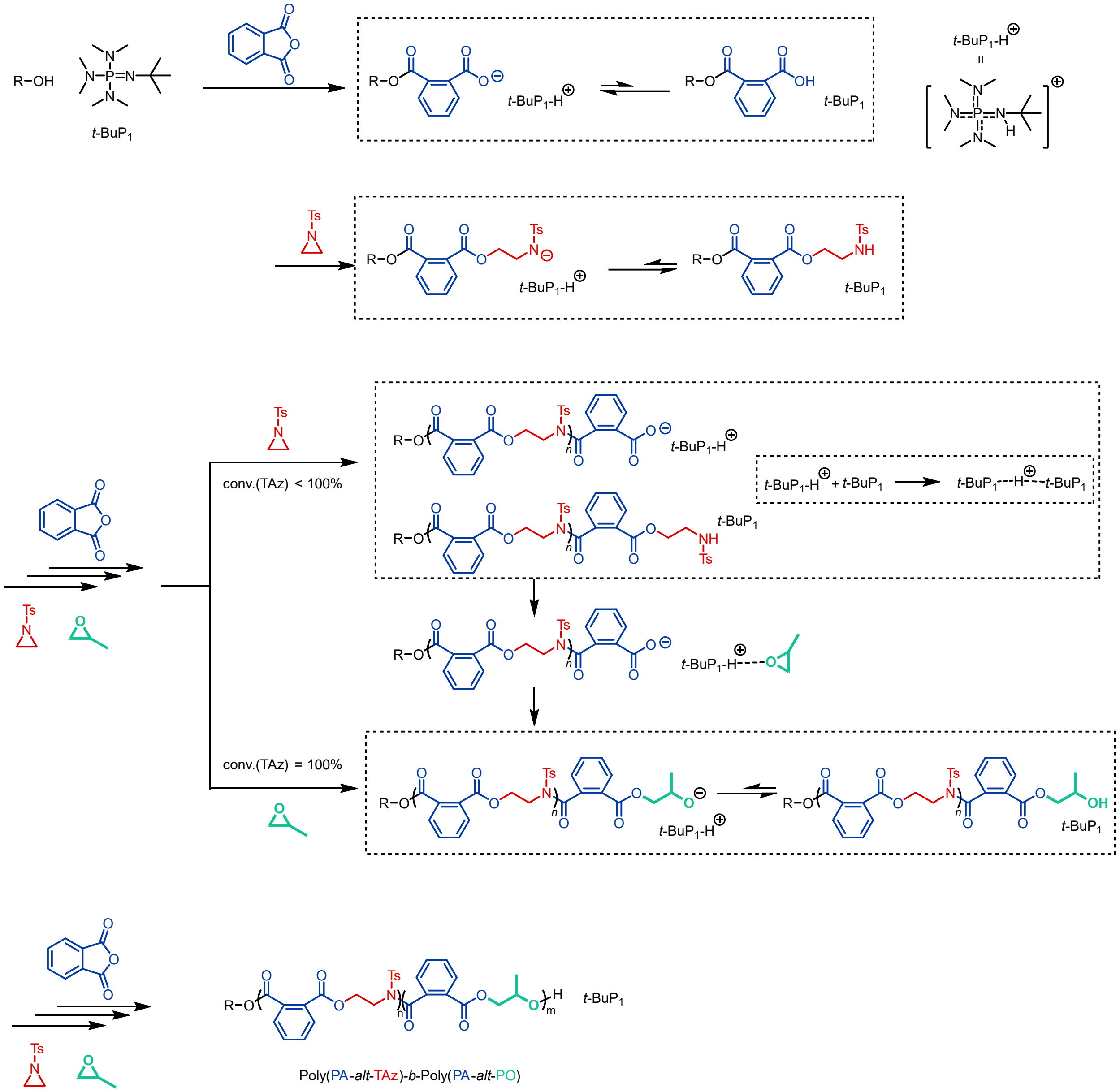

Supplementary Figure 50. Plausible mechanistic pathway for t-BuP$_1$-catalyzed terpolymerization of TAz, PA, and PO.

12.2 Stacked ^{1}H NMR spectra

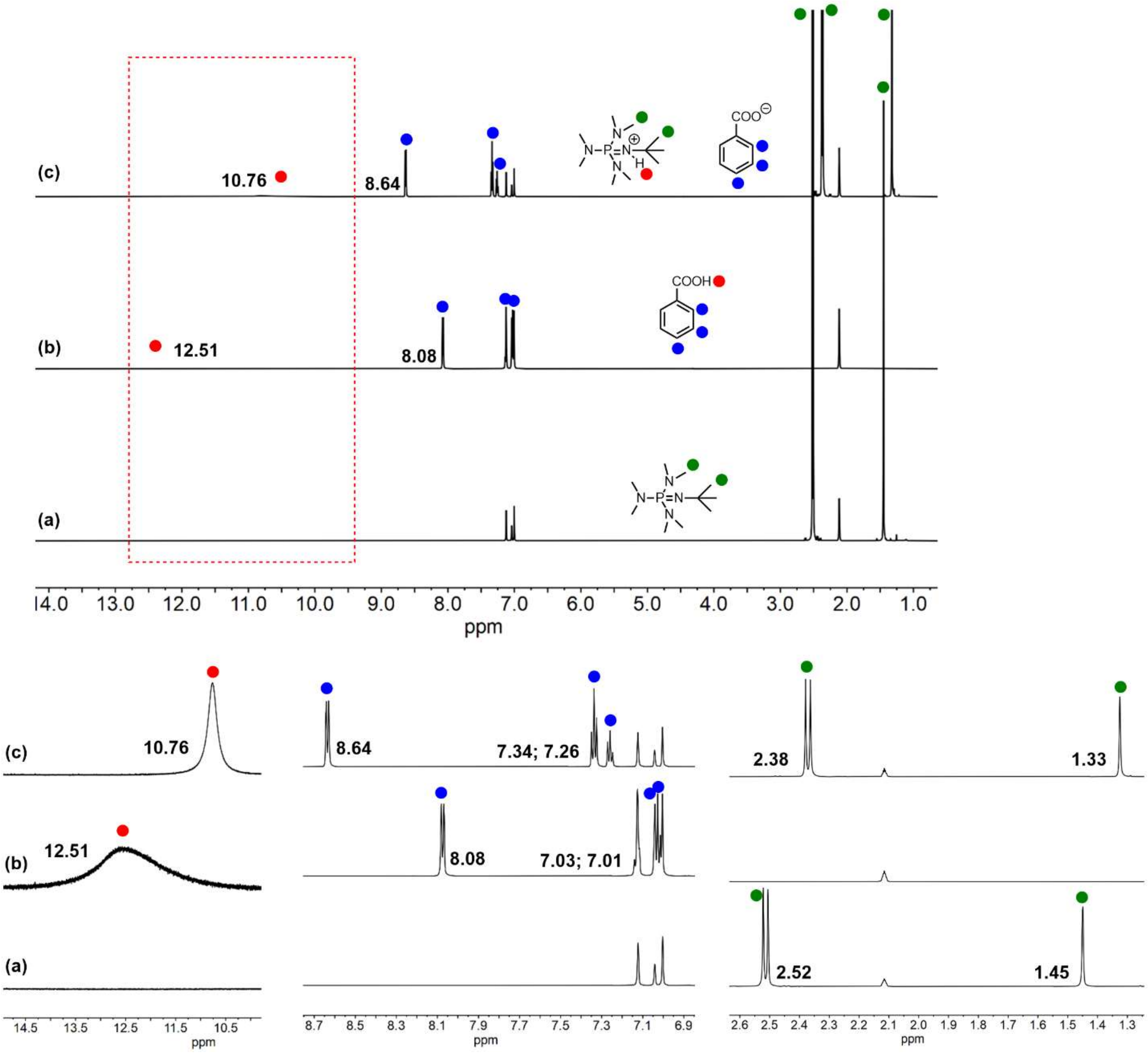

Supplementary Figure 51. Stacked ^{1}H NMR (600 MHz, toluene-d_8, 25°C) for (a) *t*-BuP$_1$, (b) BA, and (c) *t*-BuP$_1$ / BA = 1/1.

As shown in Supplementary Fig. 51, the methyl signals in *t*-BuP$_1$ shifted to the high field (green dots●), and the aromatic peaks in benzoic acid (BA) shifted to the low field (blue dots●) in the mixture of BA and *t*-BuP$_1$. In addition, the active hydrogen signal shifted from 12.51 to 10.76 ppm (red dots●). These shifts indicated that the *t*-BuP$_1$ can deprotonate the proton of BA. We concluded that the equilibrium tends to generate COO$^-$/*t*-BuP$_1$-H$^+$ due to the stronger basicity of *t*-BuP$_1$ than BA$^-$.

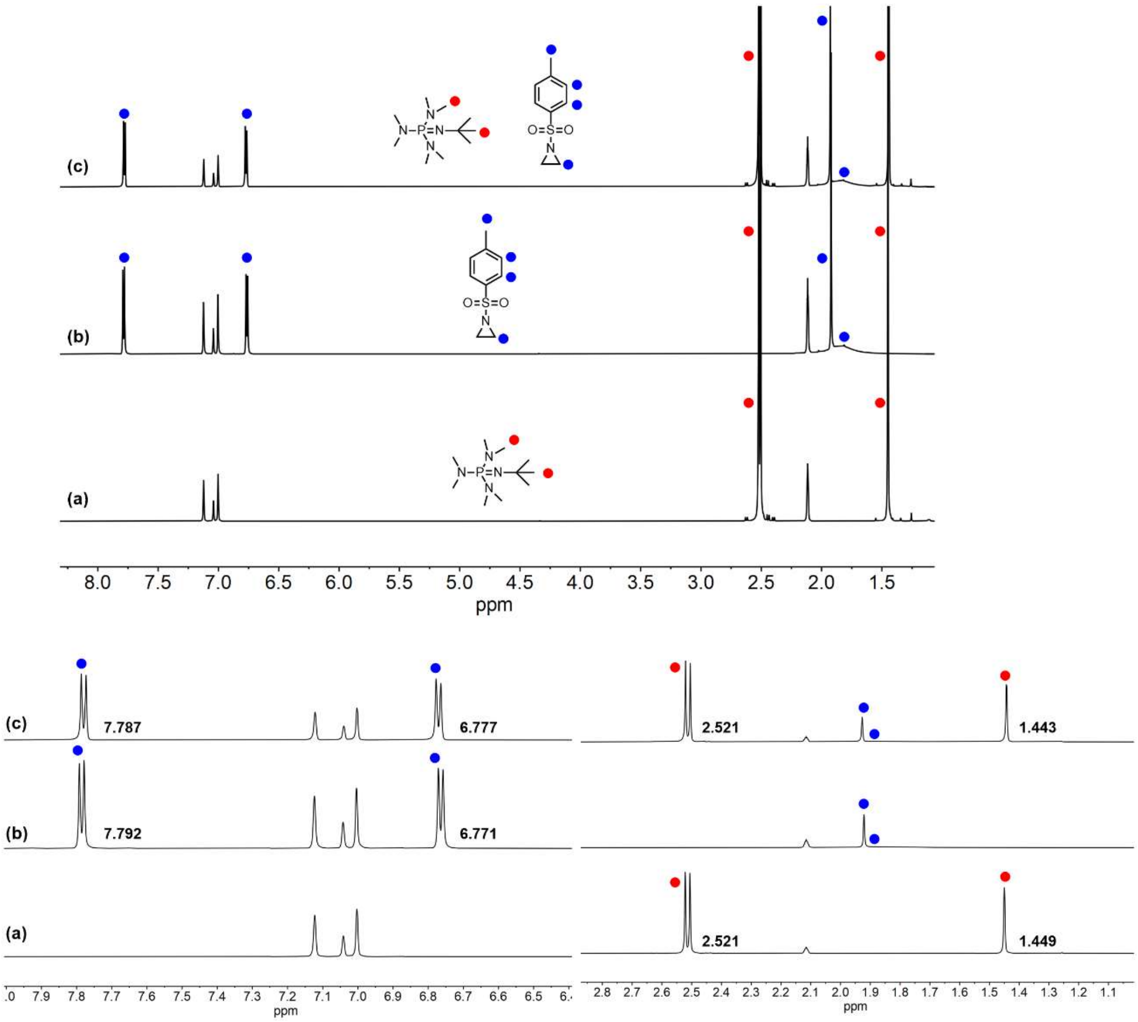

Supplementary Figure 52. Stacked ^{1}H NMR (600 MHz, toluene-d_8, 25°C) for (a) t-BuP$_1$, (b) TAz, and (c) t-BuP$_1$ / TAz = 1/1.

As shown in Supplementary Fig. 52, we mixed equivalent t-BuP$_1$ and aziridine in toluene-d_8 in Young's NMR tube under argon. There was almost no shift for the t-BuP$_1$ in the absence/presence of TAz. The results indicated that the t-BuP$_1$ cannot abstract the proton from aziridine.

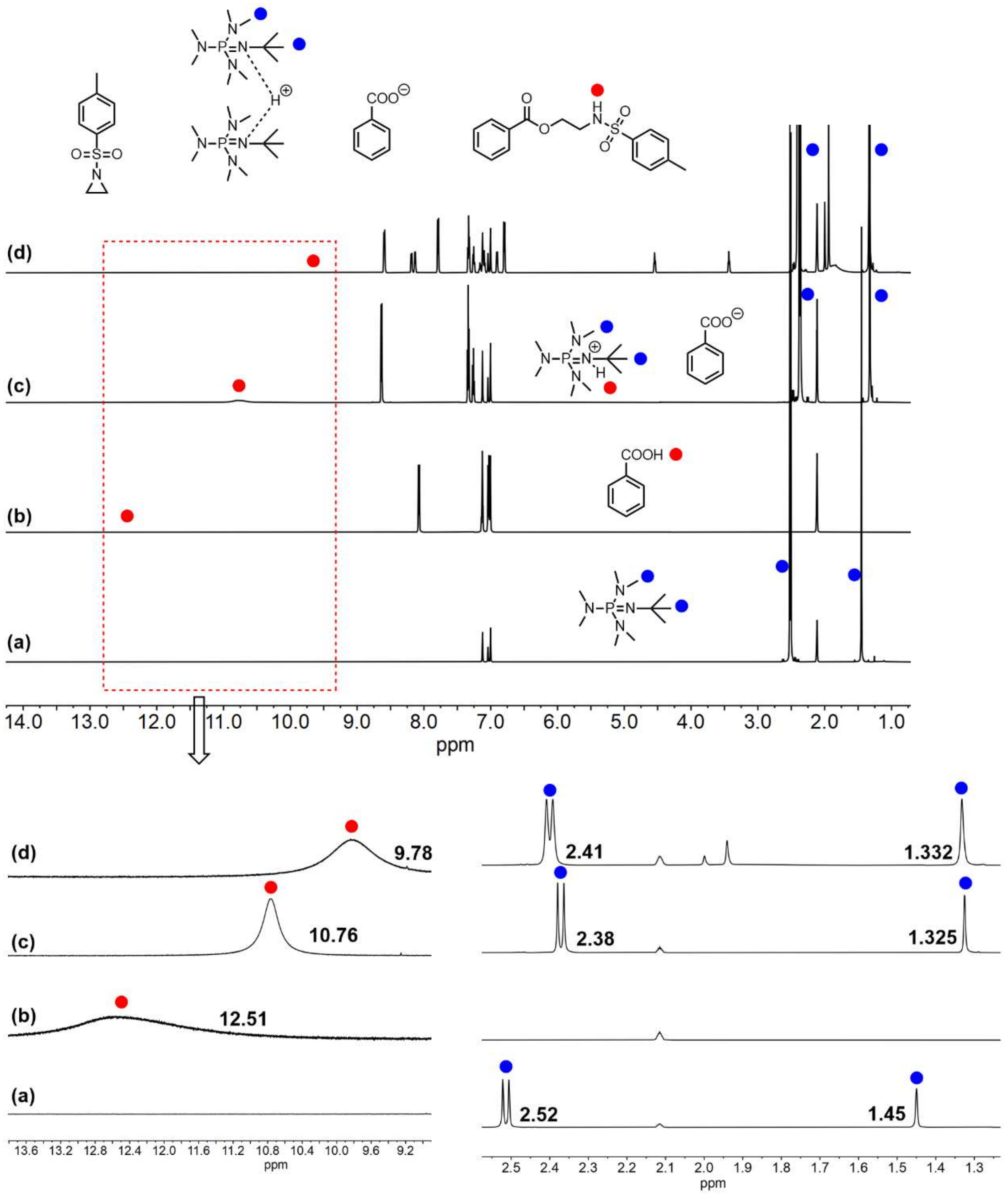

Supplementary Figure 53. Stacked ^{1}H NMR (600 MHz, toluene-d_8, 25°C) for (a) t-BuP_1, (b) BA, (c) t-BuP_1 / BA = 1/1, and (d) t-BuP_1 / BA / TAz = 1/1/1. (d: add t-BuP_1, BA, and TAz into the NMR tube).

Supplementary Fig. 46 a-c (same as Supplementary Fig. 52) demonstrated the proton transfer from BA to t-BuP_1. After the addition of TAz into the mixture of BA and t-BuP_1 in toluene-d_8 in Young's NMR tube under argon (Supplementary Fig. 53 d), the active hydrogen signals continuously shifted to the high field (red dots●, 12.51 to 10.76 to 9.78 ppm), indicating the progress of deprotonation from BA to t-BuP_1 to ring-opened TAz. After ring-opening of TAz, the proton in the t-BuP_1-H^+ is transferred to the new chain-end, forming –N(Ts)H/t-BuP_1.The methyl signals in t-BuP_1 returned to the low field slightly (blue dots●, 2.38 to 2.41 ppm), but the chemical shifts was still smaller than the original t-BuP_1 (2.52 ppm). The chemical shifts indicated the fast proton exchange between the t-BuP_1 and t-BuP_1-H^+.

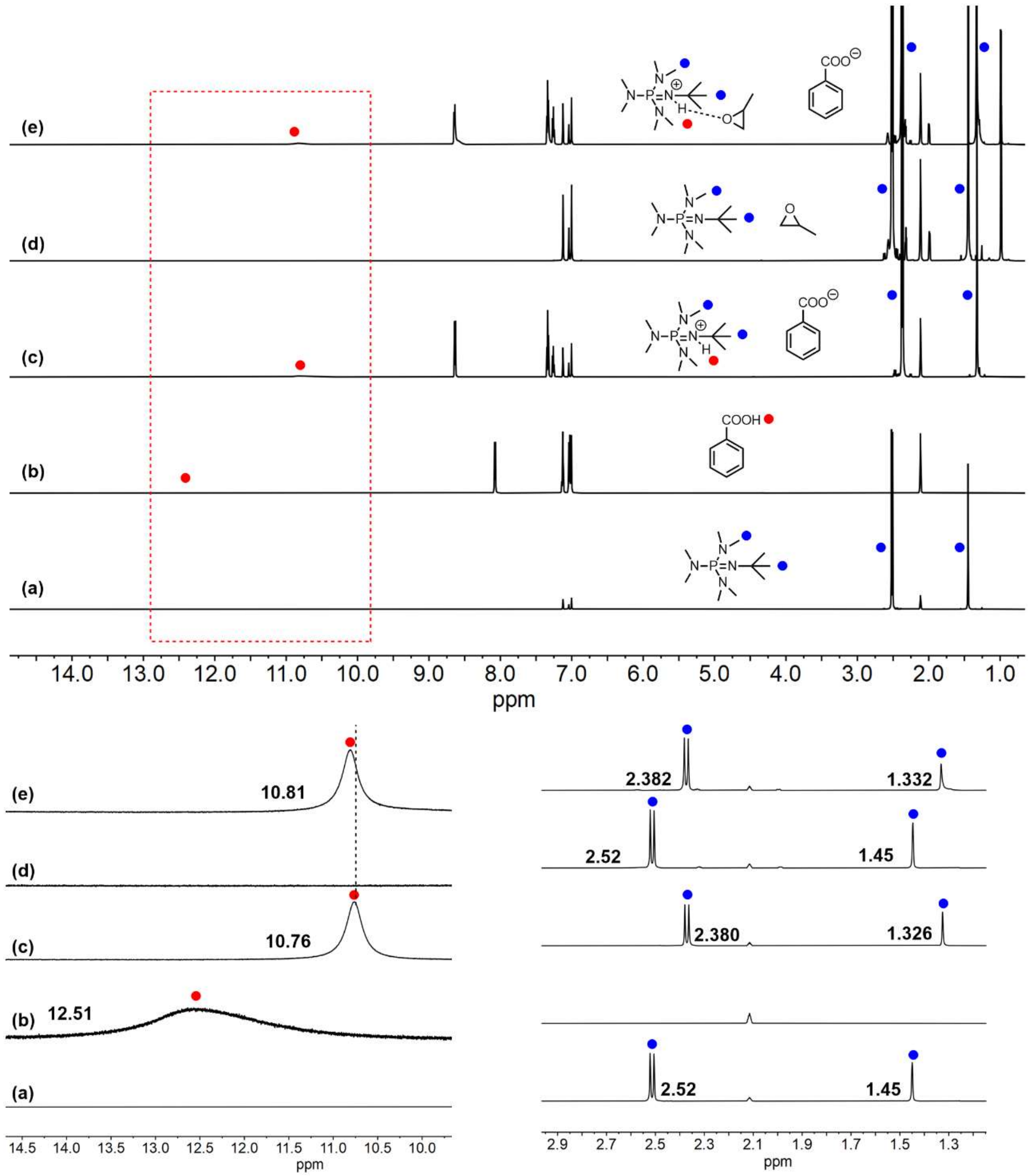

Supplementary Figure 54. Stacked ^{1}H NMR (600 MHz, toluene-d_8, 25°C) for (a) t-BuP_1, (b) BA, (c) t-BuP_1 / BA = 1/1, (d) t-BuP_1 / PO = 1/1, and (e) t-BuP_1 / BA / PO = 1/1/1.

Supplementary Fig. 54 a-c (same as Supplementary Fig. 52) demonstrated the proton transfer from BA to t-BuP_1. After mixing equivalent t-BuP_1 and PO in toluene-d_8 in Young's NMR tube under argon (Supplementary Fig. 54 d), there was almost no shift observed for the t-BuP_1 in the absence/presence of PO. The results indicated that the t-BuP_1 cannot abstract the proton from PO. After the addition of PO into the mixture of BA and t-BuP_1 in toluene-d_8 in Young's NMR tube under argon (Supplementary Fig. 54 e), the methyl and active hydrogen signals in t-BuP_1 shifted to the low field (2.380 and 1.326 to 2.382 and 1.332 ppm as well as 10.76 to 10.81 ppm), indicating hydrogen bonding between the t-BuP_1-H^+ and PO.

12.3 ^{1}H NMR spectra

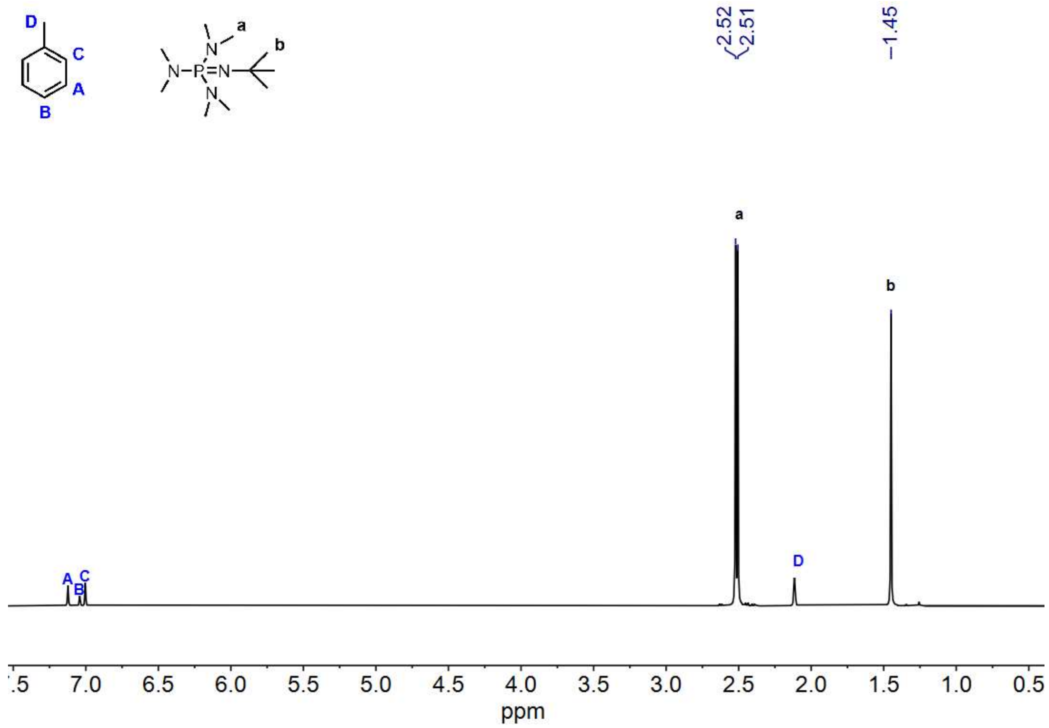

Supplementary Figure 55. ^{1}H NMR spectra (600 MHz, toluene-d_8, 25°C) of t-BuP$_1$.

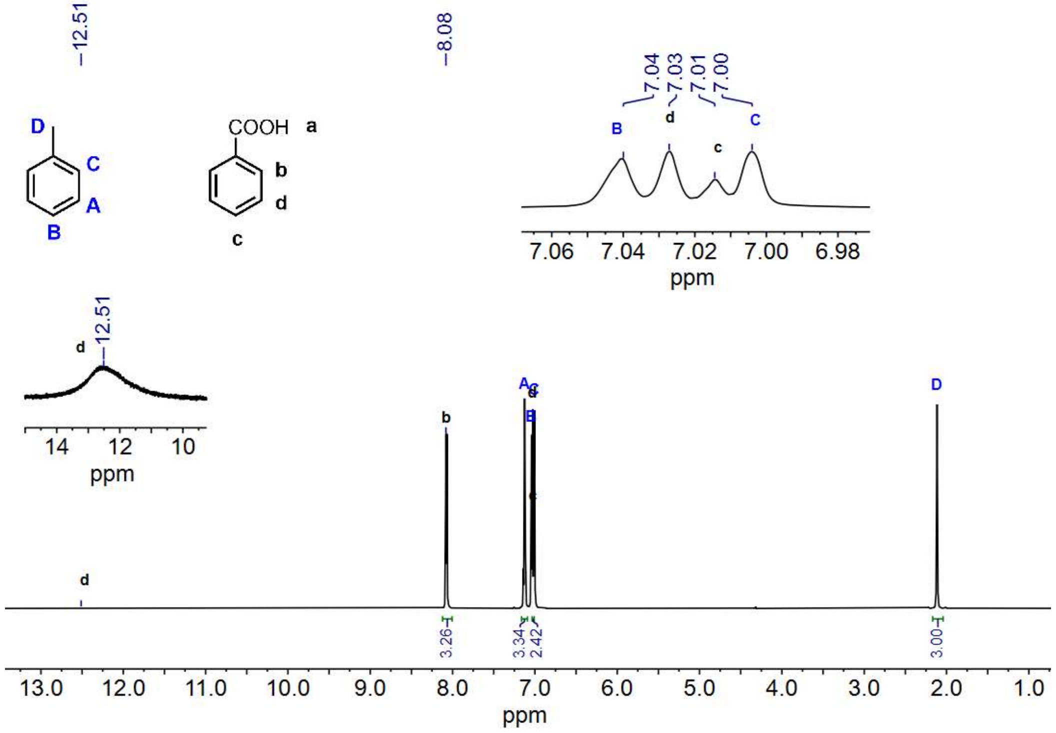

Supplementary Figure 56. ^{1}H NMR spectra (600 MHz, toluene-d_8, 25°C) of benzoic acid.

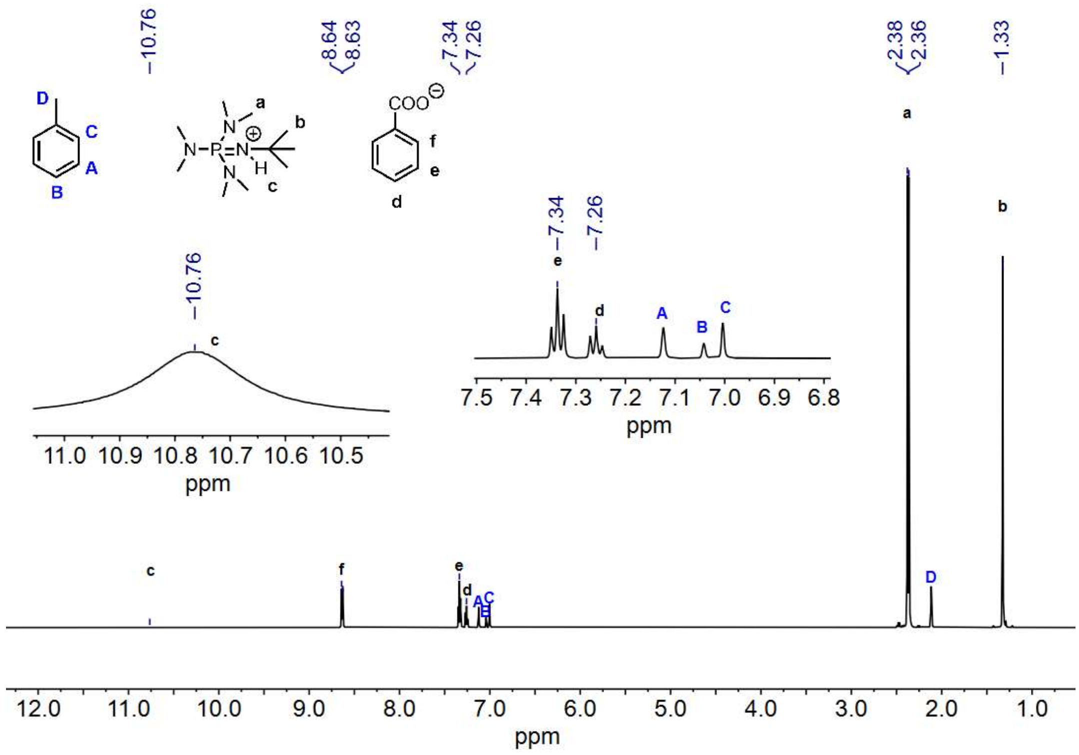

Supplementary Figure 57. ^{1}H NMR spectra (600 MHz, toluene-d_8, 25°C) of *t*-BuP$_1$ and benzoic acid at the ratio of 1:1.

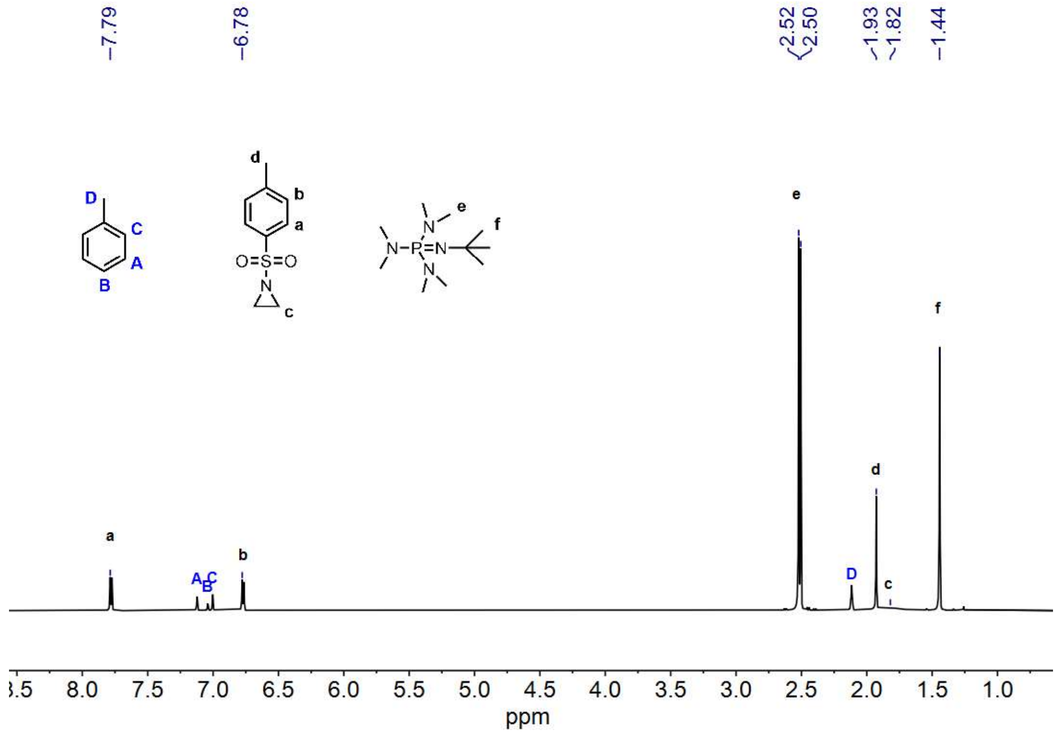

Supplementary Figure 58. ^{1}H NMR spectra (600 MHz, toluene-d_8, 25°C) of *t*-BuP$_1$ and TAz at the ratio of 1:1.

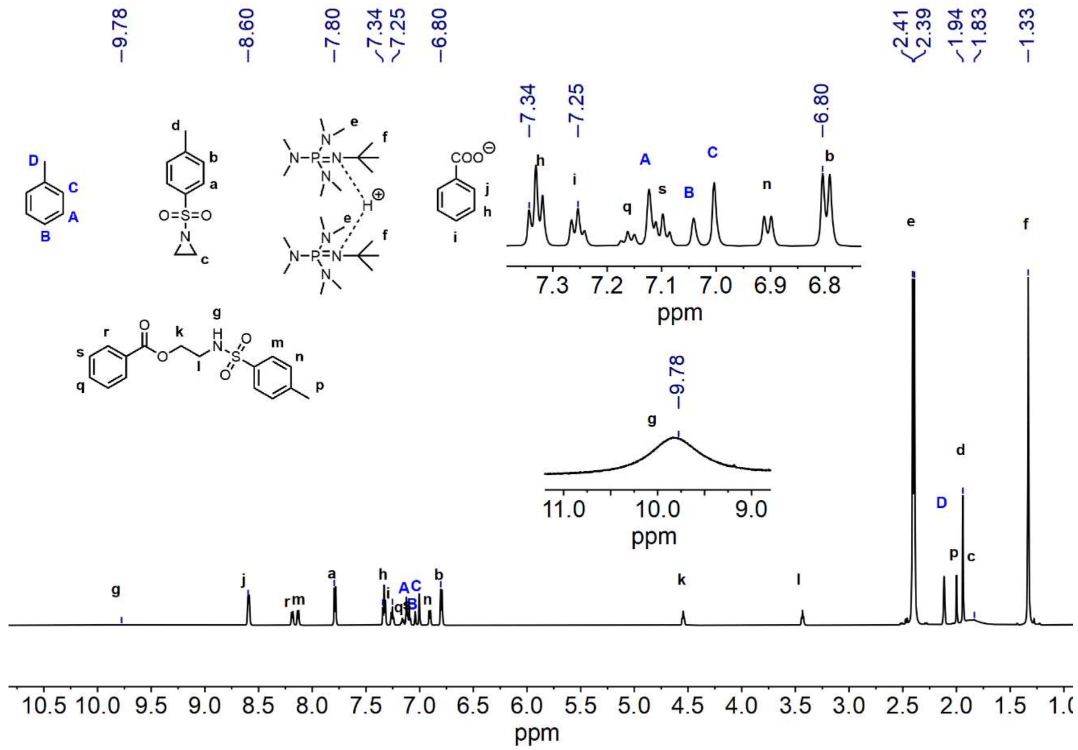

Supplementary Figure 59. ^{1}H NMR spectra (600 MHz, toluene-d_8, 25°C) of *t*-BuP$_1$, benzoic acid, and TAz at the ratio of 1:1:1.

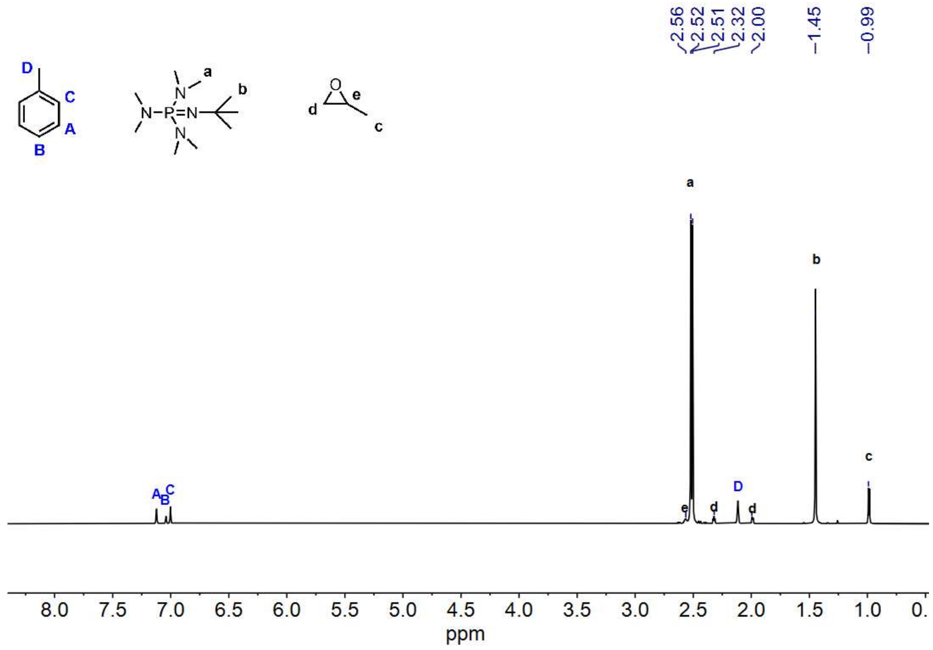

Supplementary Figure 60. ^{1}H NMR spectra (600 MHz, toluene-d_8, 25°C) of *t*-BuP$_1$ and PO at the ratio of 1:1.

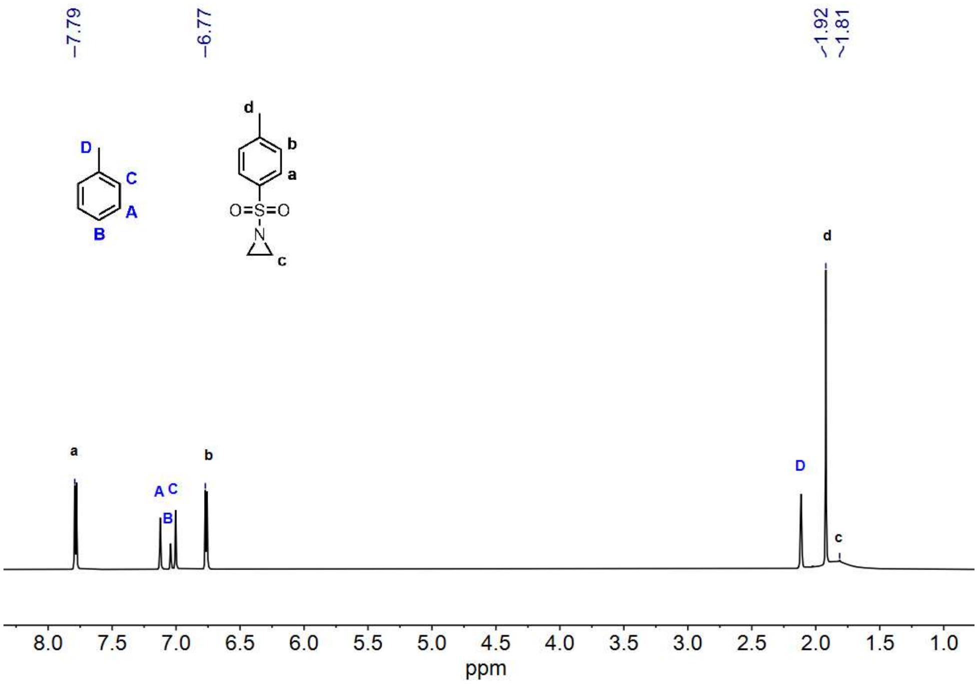

Supplementary Figure 61. ^{1}H NMR spectra (600 MHz, toluene-d_8, 25°C) of TAz.

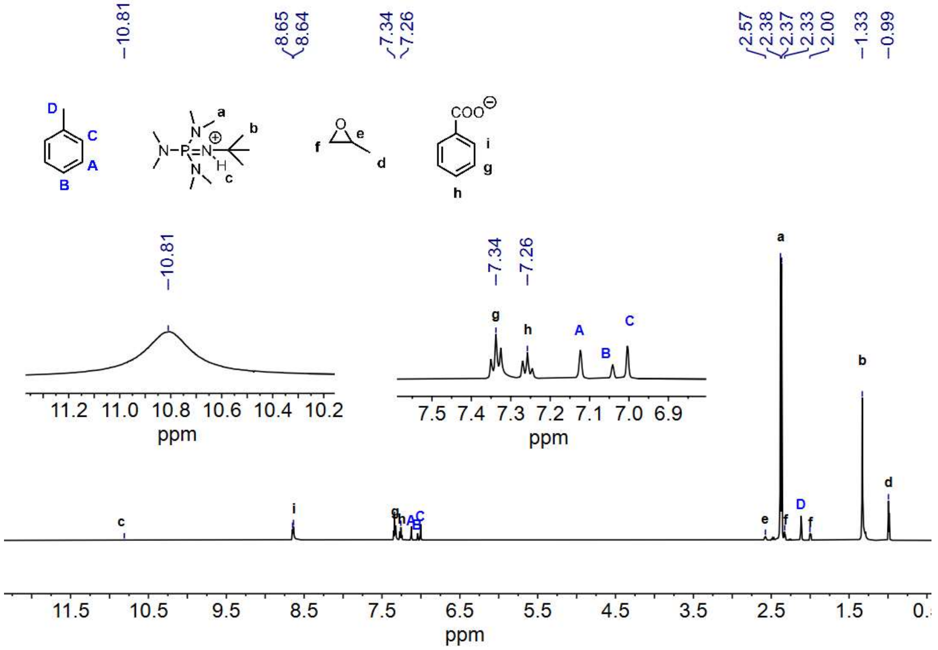

Supplementary Figure 62. ^{1}H NMR spectra (600 MHz, toluene-d_8, 25°C) of *t*-BuP$_1$, benzoic acid and PO at the ratio of 1:1:1.

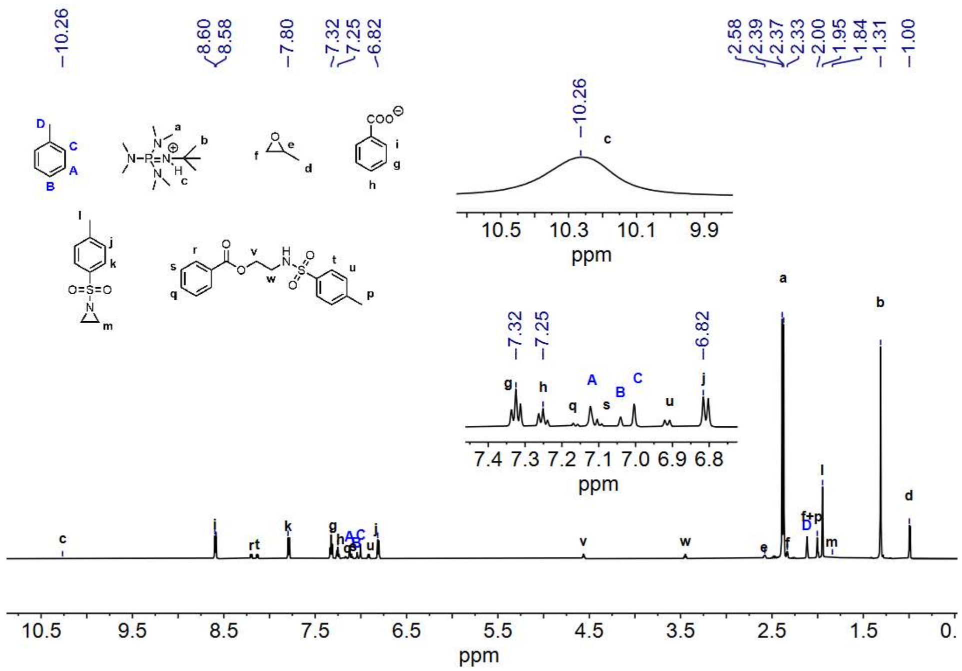

Supplementary Figure 63. ^{1}H NMR spectra (600 MHz, toluene-d_8, 25°C) of t-BuP$_1$, benzoic acid, TAz, and PO at the ratio of 1:1:1:1.

Supplementary Note 13. Thermal analysis

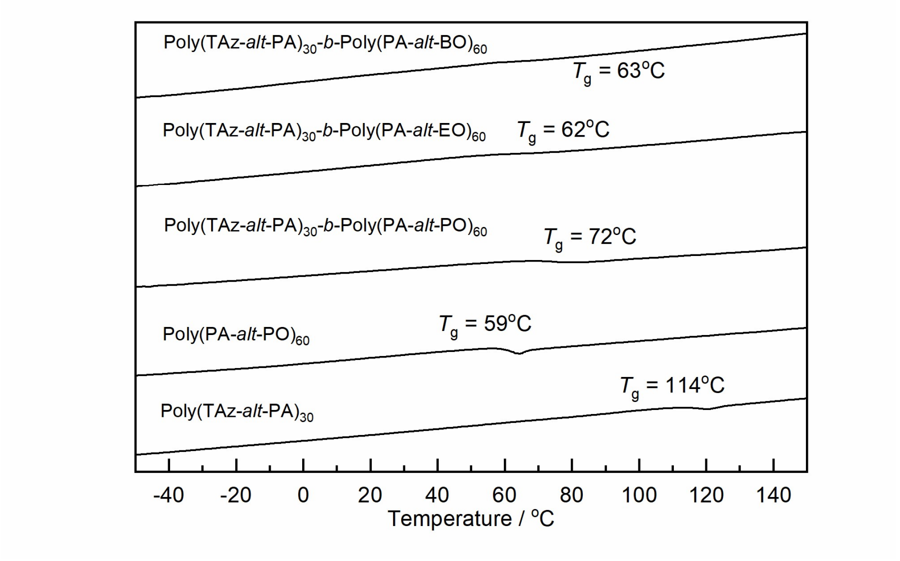

Supplementary Figure 64. DSC thermogram for poly(TAz-*alt*-PA)$_{30}$, poly(PA-*alt*-PO)$_{60}$, poly(TAz-*alt*-PA)$_{30}$-*b*-poly(PA-*alt*-PO)$_{60}$, poly(TAz-*alt*-PA)$_{30}$-*b*-poly(PA-*alt*-EO)$_{60}$, and poly(TAz-*alt*-PA)$_{30}$-*b*-poly(PA-*alt*-BO)$_{60}$ under N_2.

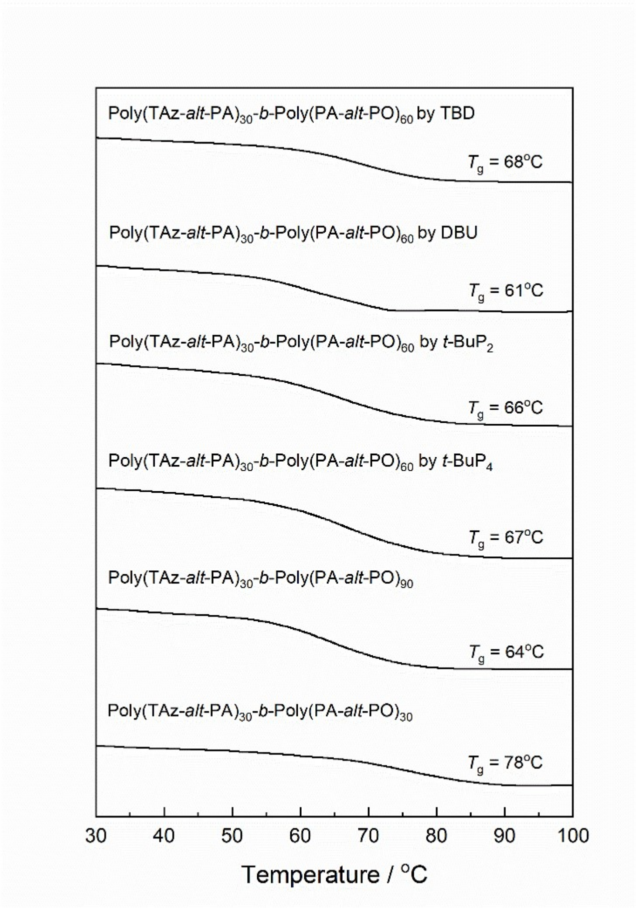

Supplementary Figure 65. DSC thermogram for poly(TAz-*alt*-PA)$_{30}$-*b*-poly(PA-*alt*-PO)$_{30}$, poly(TAz-*alt*-PA)$_{30}$-*b*-poly(PA-*alt*-PO)$_{90}$, poly(TAz-*alt*-PA)$_{30}$-*b*-poly(PA-*alt*-PO)$_{60}$ by *t*-BuP$_4$, poly(TAz-*alt*-PA)$_{30}$-*b*-poly(PA-*alt*-PO)$_{60}$ by *t*-BuP$_2$, poly(TAz-*alt*-PA)$_{30}$-*b*-poly(PA-*alt*-PO)$_{60}$ by DBU, and poly(TAz-*alt*-PA)$_{30}$-*b*-poly(PA-*alt*-PO)$_{60}$ by TBD.

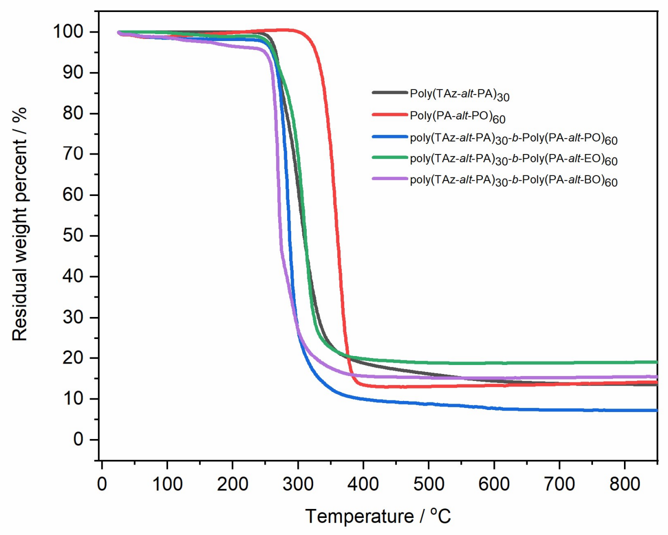

Supplementary Figure 66. TGA thermogram for poly(TAz-*alt*-PA)$_{30}$, poly(PA-*alt*-PO)$_{60}$, poly(TAz-*alt*-PA)$_{30}$-*b*-poly(PA-*alt*-PO)$_{60}$, poly(TAz-*alt*-PA)$_{30}$-*b*-poly(PA-*alt*-EO)$_{60}$, and poly(TAz-*alt*-PA)$_{30}$-*b*-poly(PA-*alt*-BO)$_{60}$.

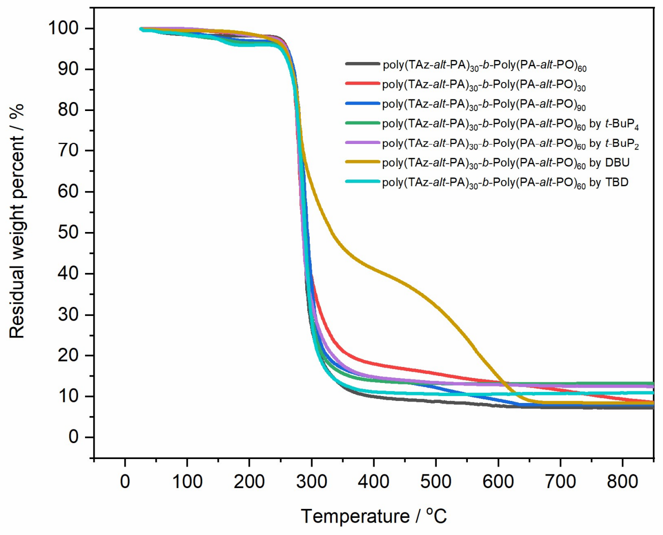

Supplementary Figure 67. TGA thermogram for poly(TAz-*alt*-PA)$_{30}$-*b*-poly(PA-*alt*-PO)$_{30}$, poly(TAz-*alt*-PA)$_{30}$-*b*-poly(PA-*alt*-PO)$_{90}$, poly(TAz-*alt*-PA)$_{30}$-*b*-poly(PA-*alt*-PO)$_{60}$ by *t*-BuP$_4$, poly(TAz-*alt*-PA)$_{30}$-*b*-poly(PA-*alt*-PO)$_{60}$ by *t*-BuP$_2$, poly(TAz-*alt*-PA)$_{30}$-*b*-poly(PA-*alt*-PO)$_{60}$ by DBU, and poly(TAz-*alt*-PA)$_{30}$-*b*-poly(PA-*alt*-PO)$_{60}$ by TBD.

Supplementary Note 14. Reactivity ratio calculation

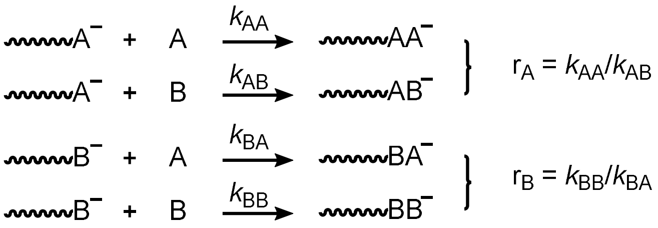

A: TAz B: PA

Definitions Mayo-Lewis equation using a steady-state assumption

$$F_A = \frac{r_A {f_A}^2 + f_A f_B}{r_A {f_A}^2 + 2 f_A f_B + r_B {f_B}^2}$$

$$F = \frac{F_A}{F_B}, \quad f = \frac{f_A}{f_B}$$

Fineman-Ross

$$(F-1)\frac{f}{F} = r_A \frac{f^2}{F} - r_B$$

Mayo Lewis

$$r_A = \frac{F}{f^2} r_B + \frac{F-1}{f}$$

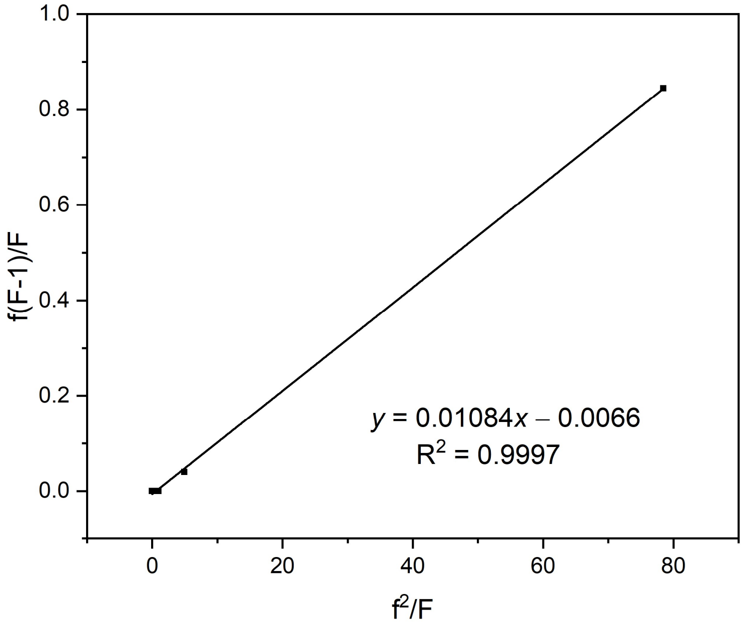

Supplementary Figure 68. Fineman-Ross evaluation of the TAz/PA ROCOP (r_{TAz} = 0.01084 and r_{PA} = 0.0066)

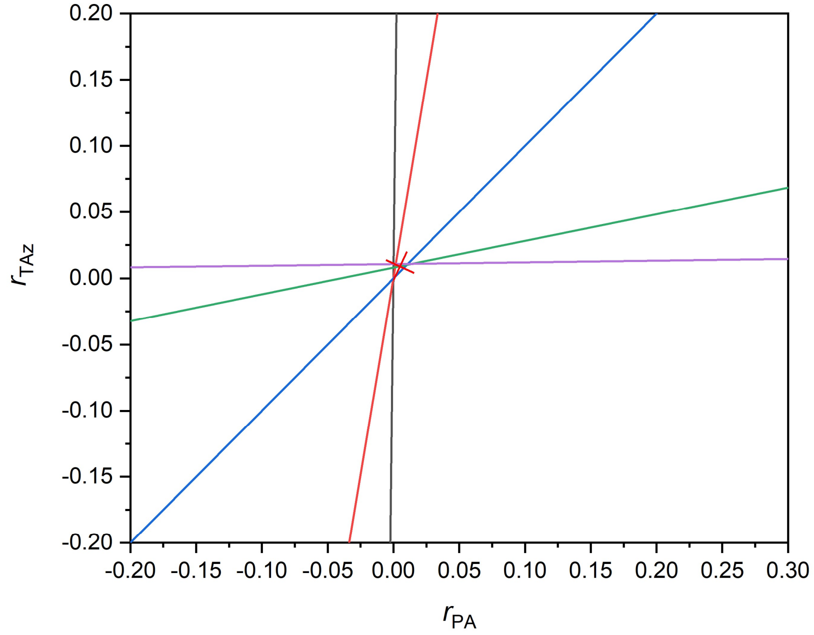

Supplementary Figure 69. Mayo-Lewis evaluation of the TAz/PA ROCOP (r_{TAz} = 0.006972 and r_{PA} = 0.003902)
